# Squaramide‐Based Heteroditopic [2]Rotaxanes for Sodium Halide Ion‐Pair Recognition

**DOI:** 10.1002/chem.202301446

**Published:** 2023-07-26

**Authors:** Arya Arun, Andrew Docker, Hui Min Tay, Paul D. Beer

**Affiliations:** ^1^ Department of Chemistry University of Oxford Chemistry Research Laboratory Mansfield Road OX1 3TA Oxford UK

**Keywords:** heteroditopic receptors, NaCl ion-pair, rotaxane, sodium cation template, squaramide

## Abstract

A series of squaramide‐based heteroditopic [2]rotaxanes consisting of isophthalamide macrocycle and squaramide axle components are synthesized using an alkali metal cation template‐directed stoppering methodology. This work highlights the unprecedented sodium cation template coordination of the Lewis basic squaramide carbonyls for interlocked structure synthesis. Extensive quantitative ^1^H NMR spectroscopic anion and ion‐pair recognition studies reveal the [2]rotaxane hosts are capable of cooperative sodium halide ion‐pair mechanical bond axle‐macrocycle component recognition, eliciting up to 20‐fold enhancements in binding strengths for bromide and iodide, wherein the Lewis basic carbonyls and Lewis acidic NH hydrogen bond donors of the squaramide axle motif operate as cation and anion receptive sites simultaneously in an ambidentate fashion. Notably, varying the length and nature of the polyether cation binding unit of the macrocycle component dramatically influences the ion‐pair binding affinities of the [2]rotaxanes, even overcoming direct contact NaCl ion‐pair binding modes in polar organic solvents. Furthermore, the cooperative ion‐pair binding properties of the squaramide‐based heteroditopic [2]rotaxanes are exploited to successfully extract solid sodium halide salts into organic media.

## Graphical abstract

**Rotaxane**! Utilizing the Lewis basic carbonyl moieties of the squaramide motif, a series of squaramide‐containing heteroditopic [2]rotaxanes are synthesized *via* sodium‐cation templation. ^1^H NMR binding studies reveal the bromide and iodide recognition capabilities of these heteroditopic receptors are significantly enhanced by up to ca. 20‐fold in the presence of co‐bound sodium cations, highlighting the unique ambidentate ion‐pair binding nature of squaramides.

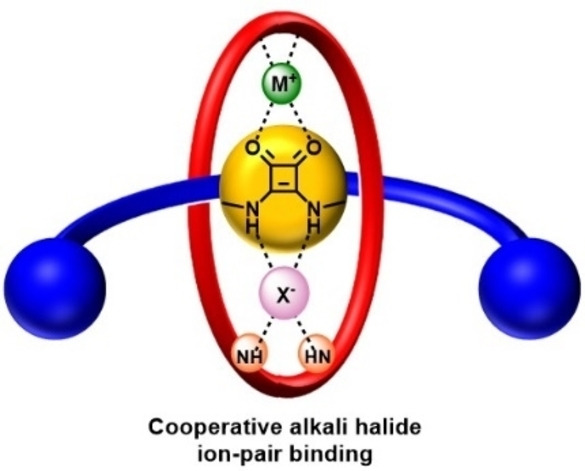

## Introduction

The numerous and fundamental roles ions play in a plethora of industrial, environmental and medical processes has continued to stimulate the development of molecular host systems for their strong and selective recognition.[^1^, ^2^, ^3^, ^4^, ^5^] Indeed, to this end heteroditopic, or ion‐pair receptors, capable of simultaneously binding cations and anions, have been the subject of continued and recent research activity.[^6^, ^7^, ^8^, ^9^, ^10^] A principal reason for the enduring interest of ion‐pair receptors is their inherent tunable cooperative enhancement of binding strength resulting from proximal co‐bound oppositely charged species and improved selectivity behaviours relative to their cation/anion binding monotopic receptor analogues.[^11^, ^12^, ^13^, ^14^, ^15^] The versatility of these receptors has prompted considerable research finding applications in salt extraction and solubilization,[^16^, ^17^, ^18^] membrane transport[^19^, ^20^, ^21^] and sensing.^[22]^ Exploiting the unique recognition environments afforded by mechanically interlocked molecule (MIM) host topologies, we[^23^, ^24^, ^25^, ^26^, ^27^] and others[^28^, ^29^, ^30^] have demonstrated notable enhancements conferred by the mechanical bond effect for charged guest species recognition relative to non‐interlocked acyclic or macrocyclic counterparts. Whilst in general, this has been directed towards anion recognition[^31^, ^32^, ^33^] recent advances have demonstrated equal promise in the first examples of heteroditopic MIMs designed to recognize ion‐pairs.[^34^, ^35^, ^36^, ^37^, ^38^]

**Figure 1:**
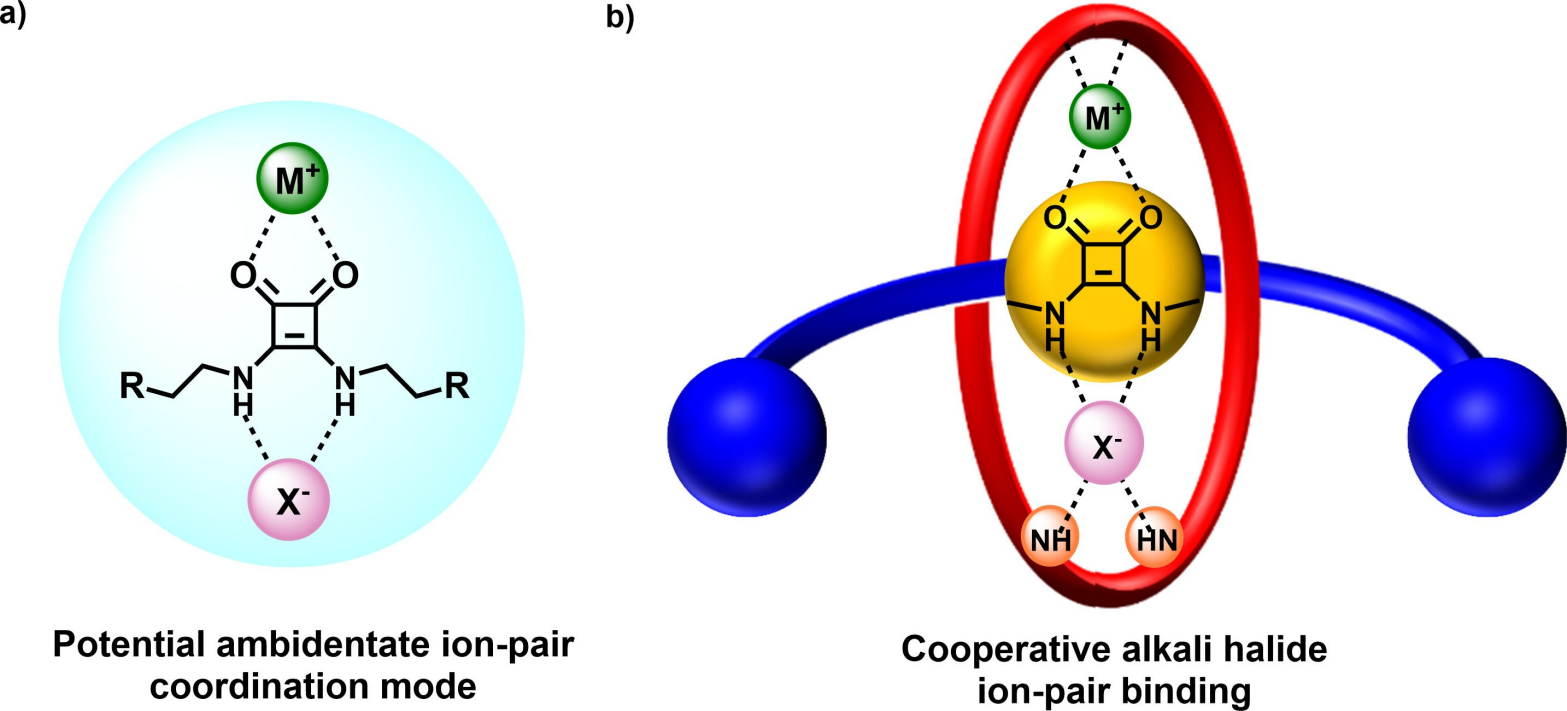
a) Potential of the squaramide motif for metal cation and ion‐pair recognition highlighting its ambidentate coordination mode utilised in this work. b) Cartoon showing ion‐pair recognition in target squaramide axle containing [2]rotaxane host systems.

A consistent challenge in the design of heteroditopic receptors is to simultaneously integrate binding motifs which can function as cation *and* anion recognition motifs. Although well known as strong, bi‐directional hydrogen bonding donor motifs for anion binding[^39^, ^40^, ^41^] surprisingly the Lewis basicity of the two carbonyl groups of squaramides has not to our knowledge been exploited for potential concomitant metal cation coordination (Figure 1a). The squaramide's combination of a potent hydrogen bond (HB) donor and carbonyl group Lewis basicity encouraged us to incorporate the motif into a heteroditopic MIM host structural framework for the purposes of ion‐pair recognition.

As a receptor for anion recognition, integration of squaramides into acyclic and macrocyclic host frameworks has previously demonstrated enormous promise with prodigious anion binding strengths frequently surpassing those of traditionally employed HB donors such as urea and thiourea analogues, and impressively in highly competitive aqueous media[^42^, ^43^, ^44^, ^45^, ^46^, ^47^] Reports of squaramide containing MIMs however are rare, with only a couple of examples described.[^48^, ^49^] Herein, we report a series of novel squaramide‐based heteroditopic [2]rotaxanes capable of cooperative sodium halide salt ion pair recognition and solid‐liquid extraction. The squaramide functionalized axle component is shown to be crucial in the ion‐pair recognition properties, simultaneously binding a sodium cation *via* bidentate chelation of the squaramide carbonyl groups and anion binding through NH hydrogen bond donors supplemented by the rotaxane's isophthalamide macrocycle component (Figure 1b).

## Results and Discussion

### Design and synthesis

**Figure 2:**
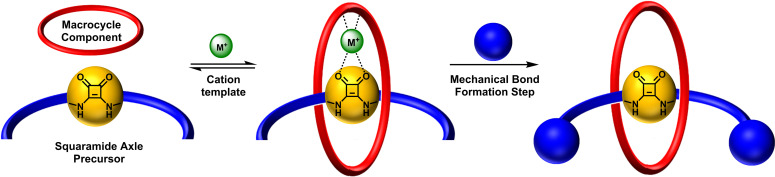
Schematic showing synthesis of the squaramide‐based [2]rotaxane host systems.

**Figure 3:**
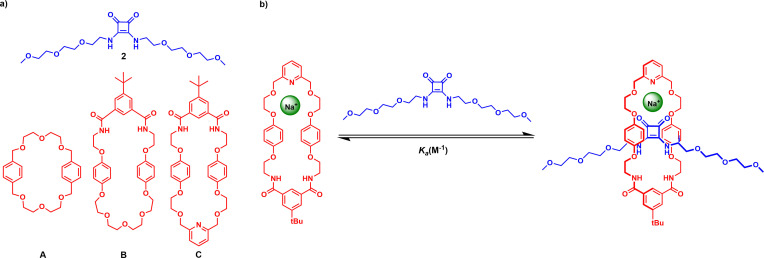
a) Axle precursor and macrocycles used in the pseudo‐[2]rotaxane studies. b) Schematic showing the proposed formation of sodium complexed pseudo‐[2]rotaxanes.

Extending the inspired approach developed by Chiu for alkali metal template MIM construction,^[50]^ the target heteroditopic squaramide containing [2]rotaxanes were synthesized *via* an unprecedented sodium cation template‐directed coordination of a squaramide axle precursor‐macrocycle polyether pseudo[2]rotaxane assembly. Wherein it was envisaged the target [2]rotaxanes could be prepared *via* a double stoppering methodology (Figure 2). To investigate this strategy, we identified three potentially suitable macrocycles which contain either crown ether‐like arrangements of oxygen donor atoms as in **A**,^[51]^ or mixed polyether and pyridyl isophthalamide cyclic linkages, **B** ^[52]^ and **C**,^[53]^ respectively (Figure 3a).

Quantitative sodium cation template interpenetrative assembly ^1^H NMR spectroscopic investigations between a model ethylene glycol‐appended squaramide axle precursor **2** and all three macrocycles were initially undertaken to establish requisite pseudo[2]rotaxane formation. In a typical ^1^H NMR titration procedure, aliquots of squaramide **2** were added to a CDCl_3_ solution of the respective macrocycle in the presence of one equivalent of NaBAr^F^_4_ (Figure 3b). In general, the addition of increasing amounts of **2** to the Na^+^ complexed macrocycles induced considerable shifts in the aromatic spacer proton signals of the macrocycle and the methylene groups of the squaramide threading component, indicative of interpenetration. Bindfit^[54]^ analysis of the respective binding isotherms determined 1 : 1 stoichiometric host–guest association constants (*K*_a_) which are summarized in Table 1.

**Table 1:** Apparent association constants *K*_a_ (M^−1^) of macrocycles **A**, **B** and **C** with **2** in absence or presence of one equiv. of NaBAr^F^_4_ in CDCl_3_ at 298 K.^[a]^

	Template	Mac. **A**	Mac. **B**	Mac. **C**
SQ thread **2**	–^[b]^	–^[c]^	36	16
	NaBAr^F^_4_	189	1278(14)	1849(21)

[a] *K_a_* values calculated using Bindfit software using a 1 : 1 host–guest binding model. Error percentages less than 10 % unless specified. Global fit performed for all systems. [b] No template. [c] No binding.

Inspection of the determined *K*_a_ values revealed that crown‐ether like macrocycle **A** exhibits a modest association, whilst the isophthalamide containing macrocycles, **B** and **C** displayed considerably larger *K*_a_ values of 1278 M^−1^ and 1849 M^−1^ respectively. It is important to note that in the absence of a templating sodium cation no ^1^H NMR evidence for pseudorotaxane assembly was observed for macrocycle **A** and only very weak association constant *K*_a_ (<50 M^−1^) values were determined for macrocycles **B** and **C**, which presumably arises as a result of HB interactions between the macrocycle isophthalamide HB donor and the Lewis basic carbonyls of the squaramide threading component.

**Scheme 1:**
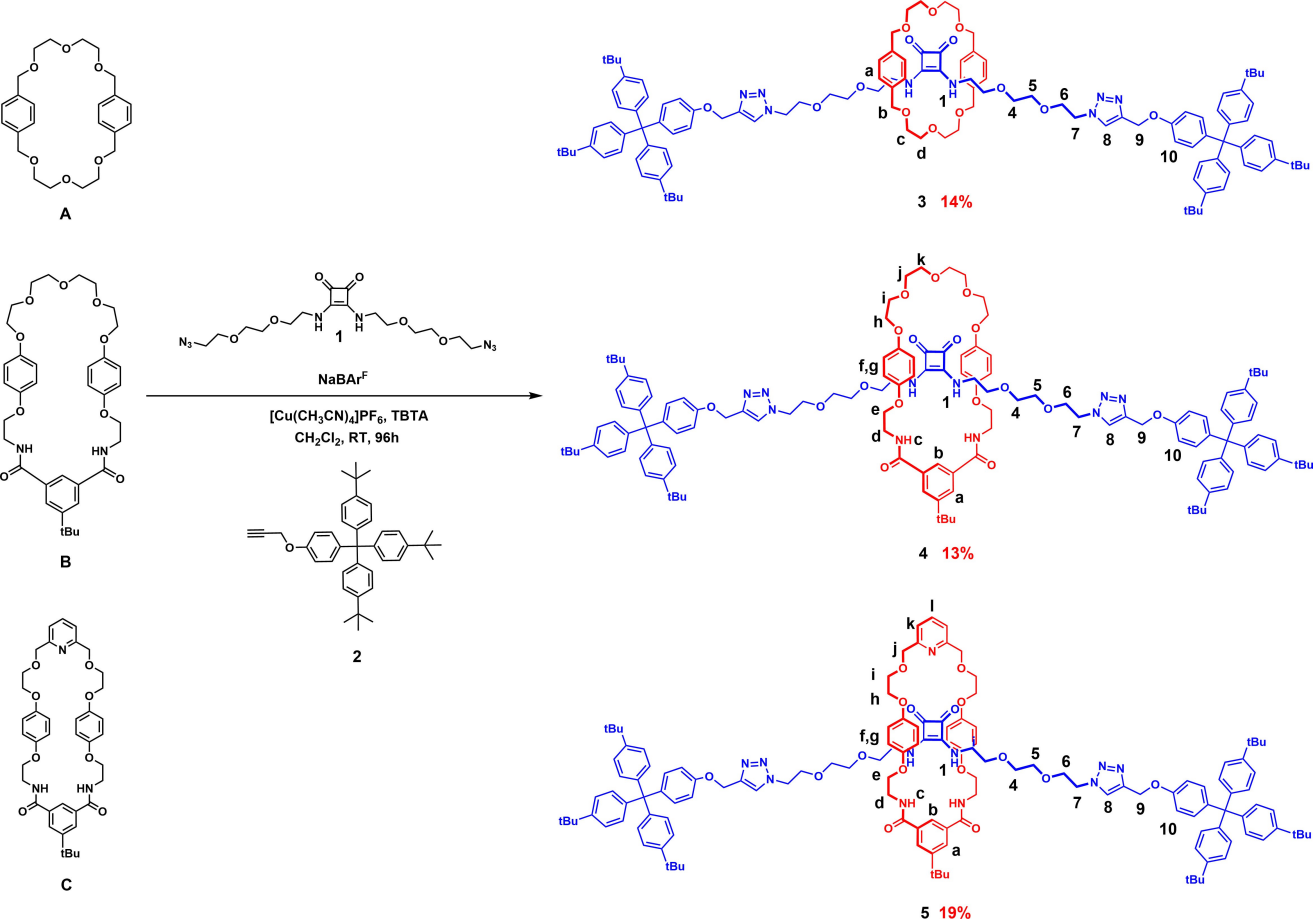
Synthesis of heteroditopic [2]rotaxanes **6**–**8**.

Having established ^1^H NMR evidence for pseudo[2]rotaxane formation, the Na^+^ template directed synthesis of the target squaramide [2]rotaxanes were undertaken *via* a copper(I) catalysed alkyne‐azide cycloaddition (CuAAC)‐mediated double stoppering reaction strategy (Scheme 1). In a typical reaction, a macrocycle (**A**, **B** or **C**) and bis‐azide appended squaramide **4** were pre‐complexed with one equivalent of NaBAr^F^_4_ in CH_2_Cl_2_ for 30 min to form the pseudo[2]rotaxane assembly, to which was added a mixture of terphenyl stopper alkyne **5** ^[55]^ and catalytic amounts of Cu[(CH_3_CN)_4_]PF_6_ and the rate accelerating agent TBTA.^[56]^ The reaction mixture was left stirring for four days at room temperature. After an aqueous workup procedure and purification by preparative TLC, the target [2]rotaxanes **6**, **7** and **8** were isolated in 14, 13 and 19 % respective yields.

**Figure 4:**
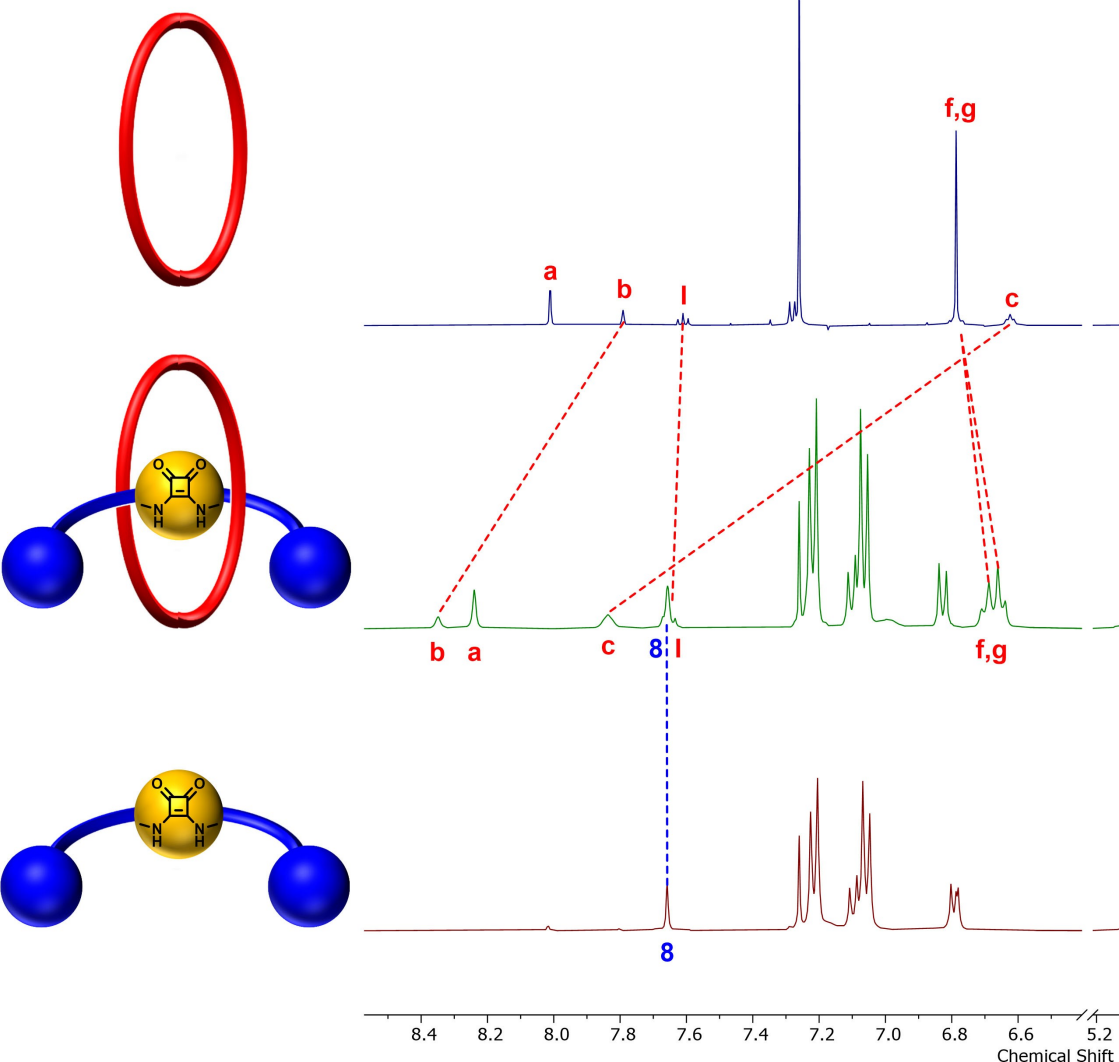
Truncated stacked ^1^H NMR spectra of i) macrocycle **C**, ii) [2]rotaxane **8**, and iii) axle component **8 a** (CDCl_3_, 500 MHz, 298 K).

Successful formation of the target [2]rotaxanes was evidenced by their ^1^H NMR spectra, compared to that of their respective free macrocycle and axle components. A representative example is shown for **8** which reveals significant downfield shifts of the internal cavity protons of the [2]rotaxane such as protons H_b_ and H_c_ upon mechanical bond formation (Figure 4). Furthermore, the hydroquinone protons of the macrocycle split and shift upfield owing to donor‐acceptor interactions between the electron‐rich macrocycle component's hydroquinone groups and electron‐deficient squaramide axle component. Similar diagnostic shifts were observed for the other rotaxanes **6** and **7**.

**Figure 5:**
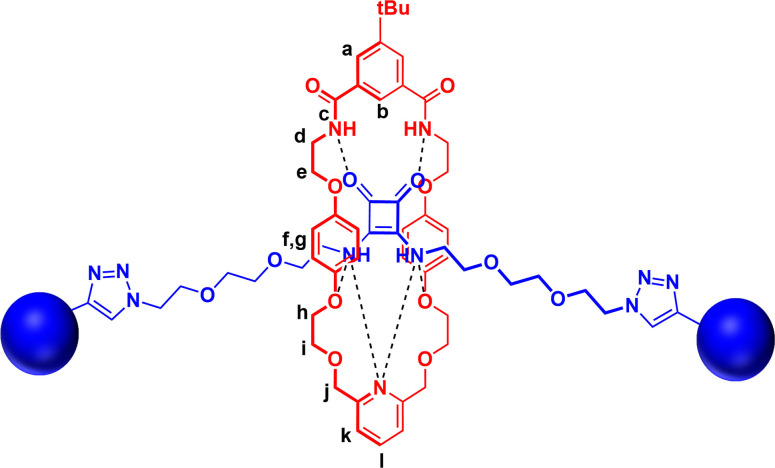
Possible co‐conformation of [2]rotaxane **8** as conferred by ^1^H‐^1^H ROESY NMR study results.

All three novel [2]rotaxanes were characterised *via* ^1^H, ^13^C NMR, ^1^H‐^1^H ROESY NMR and HRMS (see Supporting Information). Interestingly, the ^1^H‐^1^H ROESY NMR study in CDCl_3_ revealed through space coupling between the axle squaramide NH protons and the macrocycle protons (H_b_, H_h_, H_i_ and H_j_) while none was observed between the axle triazole protons and the protons of the macrocycle (Figure S19). This suggests the macrocycle resides at the squaramide centre of the axle, which is likely due to favourable intercomponent hydrogen bonding interactions between the axle‐squaramide HB NH donors and polyether or pyridyl linking groups of the macrocycles (Figure 5).

### Cation, anion and ion‐pair binding studies

With the target [2]rotaxanes in hand, attention was directed towards investigating the cation, anion and ion‐pair recognition properties of the interlocked hosts. To firstly establish the cation binding properties of the [2]rotaxanes, ^1^H NMR titration studies were conducted by adding aliquots of NaBAr^F^_4_ to a 1 mM solution of each rotaxane in 3 : 7 CD_3_CN/CDCl_3_ solvent media.

**Figure 6:**
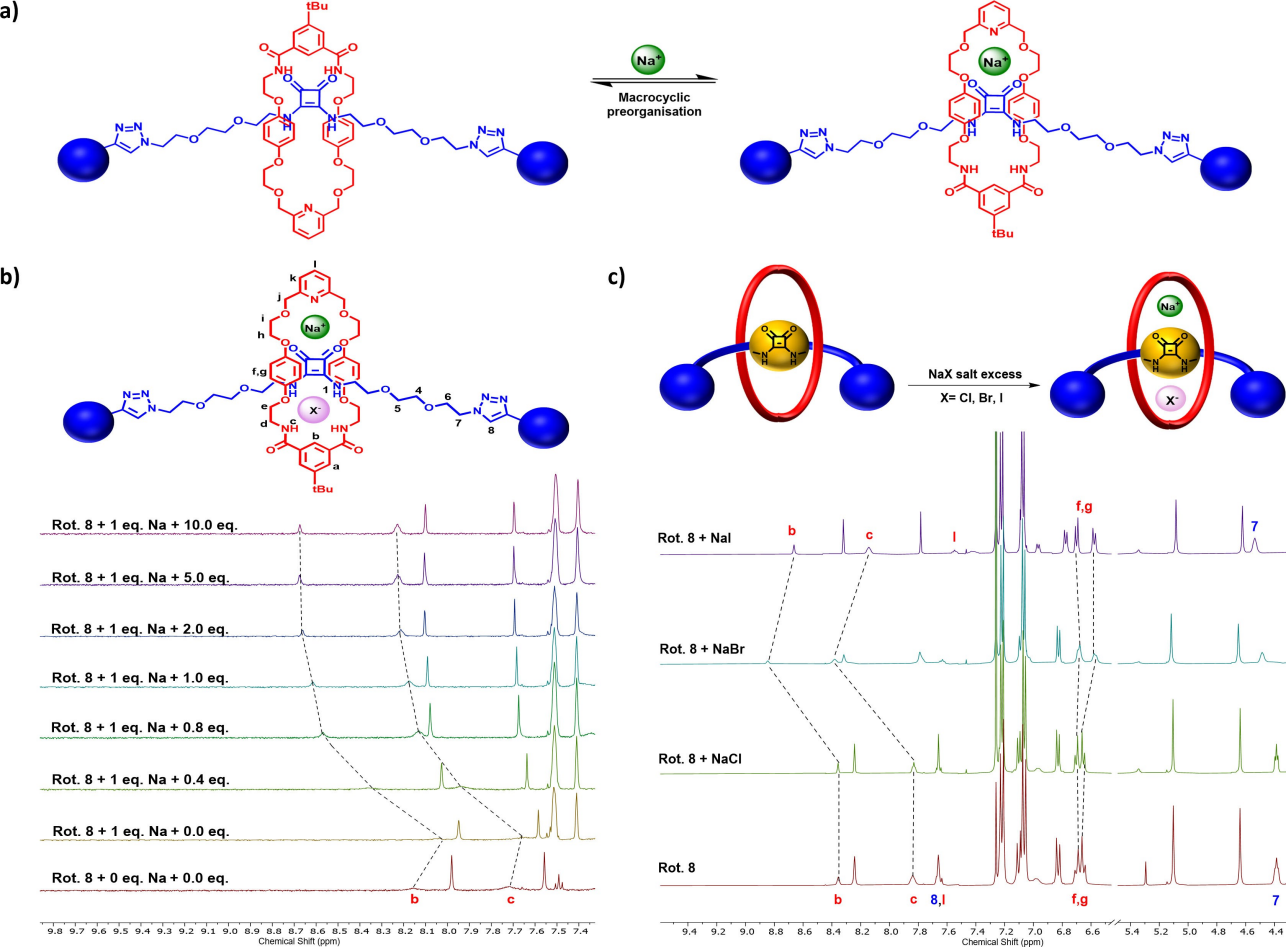
a) Proposed macrocyclic preorganization occurring in [2]rotaxane **8** on addition of one equivalent of NaBAr^F^_4_ b) Truncated ^1^H NMR spectra of rotaxane [2]rotaxane **8** in the presence of 1 equiv. of NaBAr^F^_4_ with increasing equivalents of TBABr in 3 : 7 CD_3_CN‐CDCl_3_ (500 MHz, 298 K) c) Pre‐ and post‐extraction spectra of [2]rotaxane **8** with excess NaCl, NaBr and NaI (CDCl_3_, 500 MHz, 298 K).

Upon increasing sodium cation concentration, perturbations in the resonances corresponding to the ethylene glycol protons of the axle and macrocycles were observed suggesting that alkali metal cation binding occurs *via* the polyether oxygens in the macrocycle in concert with those in the axle. As for [2]rotaxane **8**, the pyridyl group proton signals of the macrocycle were also observed to undergo significant perturbations. Additionally, the upfield movement of the internal benzene proton H_b_ and amide protons H_c_ of the respective rotaxane macrocycle components **B** and **C** indicated that the binding of the metal cation to the receptor presumably serves to disrupt the intermolecular HB interactions between the squaramide NH protons and the macrocycles (Figure 6a). Further evidence of the postulated sodium cation binding mode was obtained *via* qualitative ^13^C NMR titration studies conducted in CDCl_3_. The addition of one equivalent of NaBAr^F^_4_ to [2]rotaxane **8** resulted in the characteristic axle squaramide cyclobutenedione peaks at 183 ppm and 168 ppm undergoing significant upfield shifts, suggesting that the squaramide carbonyls of the rotaxane participate in sodium cation binding (Figure S40). Importantly, this thereby serves to preorganize the anion binding cavity such that all amide donors can concertedly bind guest anions. Bindfit analysis of the titration isotherm data determined 1 : 1 stoichiometric rotaxane host‐sodium cation guest binding constants summarized in Table 2.

**Table 2:** Cation and anion association constants *K*_a_ (M^−1^) for [2]rotaxanes 6–8 [1.0 mM] in 3 : 7 CD_3_CN/CDCl_3_ at 298 K.^[a]^

Cation	Anion	[2]Rotaxane **6**	[2]Rotaxane **7**	[2]Rotaxane **8**
NaBAr^F^_4_	–	>10^4^	1193	1506
–	Cl^−^	163	476	543
	Br^−^	204	580	494
	I^−^	–^[b]^	154	67

[a] *K*_a_ values calculated using Bindfit software using a 1 : 1 host–guest binding model. All anions added as their TBA salts. Error percentages less than 10 % unless specified. Global fit performed for all systems. [b] No binding.

Notably Na^+^ binding with [2]rotaxane **6** was too strong to be quantified, which is ascribed to a more complementary size fit between Na^+^ and the smaller interlocked cavity binding site of **6**. Interestingly, the integration of a pyridyl dimethoxy motif in the macrocycle component of rotaxane **8** is accompanied with an increase in *K*_a_ (Na^+^) relative to its polyether containing rotaxane analogue **7**.

The halide anion binding properties of the [2]rotaxanes were also investigated by carrying out analogous titration experiments with tetratbutylammonium (TBA) halide salts in the same 3 : 7 CD_3_CN/CDCl_3_ solvent media. The addition of the lighter halides, chloride or bromide to [2]rotaxane **6** induced significant downfield shifts of the axle squaramide NH protons H_1_ and upfield shifts of the macrocycle xylene protons H_a_, indicating that anion binding occurs in the interlocked cavity of the [2]rotaxane. By contrast no evidence of iodide binding was observed. With [2]rotaxanes **7** and **8**, upon increasing halide anion concentration, the respective macrocycle amide protons H_c_ and axle squaramide NH protons H_1_ moved progressively downfield, indicating that binding occurs in the rotaxane host HB donor cavity. Notably, in the case of all [2]rotaxanes, no significant perturbations in the axle triazole protons were seen suggesting it does not participate in anion binding. Bindfit analysis of the titration isotherms generated anion binding constants using a 1 : 1 stoichiometric host–guest binding model summarized in Table 2. Interestingly, inspection of the determined *K*_a_ values reveals [2]rotaxanes **6** and **7** to exhibit a preference for bromide whereas [2]rotaxane **8** bound chloride the strongest. Predictably, the anion binding affinities of [2]rotaxane **6** were lower than those of [2]rotaxanes **7** and **8** for all three halides, consistent with the lack of additional HB donor motifs in the macrocycle component of **6**.

With the individual sodium cation and halide anion recognition behaviour determined, we next sought to explore the sodium halide ion‐pair binding properties of the [2]rotaxanes which were investigated by adding aliquots of TBA halide salts to a 1 mM solution of each rotaxane in the presence of 1 equivalent of NaBAr^F^_4_ in 3 : 7 CD_3_CN/CDCl_3_. Considering first the ion‐pair binding behaviour of NaBr and NaI, upon the addition of bromide and iodide to the [2]rotaxanes, significant downfield perturbations in the protons involved in the anion binding cavity were observed, namely the internal benzene proton H_b_ and amide protons H_c_ in the case of [2]rotaxanes **7** and **8** (Figure 6b) and the squaramide NH protons H_1_ in the case of [2]rotaxane **6**, which became non‐visible in the case of the other rotaxanes, presumably due to strong hydrogen bonding induced broadening. Furthermore, perturbations were also observed in the cation binding regions of the [2]rotaxanes, confirming that the anion binding event enhanced the binding of the cation. Monitoring the perturbations of the proton signals proximal to the anion binding site by Bindfit analysis determined 1 : 1 host: guest stoichiometric apparent association constants summarized in Table 3.

**Table 3:** Apparent anion association constants *K*_a_ (M−1) for [2]rotaxanes **6**–**8** [1.0 mM] in the presence of 1 equiv. NaBAr^F^_4_ in 3 : 7 CD_3_CN/CDCl_3_ at 298 K.^[a]^

Host	Cation	%bound	Anion	*K*_a_ [M^−1^]
[2]Rotaxane **6**	NaBAr^F^_4_ (1 equiv.)	>75 %	Cl^−^	–^[b]^
			Br^−^	1307
			I^−^	367
[2]Rotaxane **7**	NaBAr^F^_4_ (1 equiv.)	41 %	Cl^−^	–^[b]^
			Br^−^	>10^4^
			I^−^	1476
[2]Rotaxane **8**	NaBAr^F^_4_ (1 equiv.)	45 %	Cl^−^	646
			Br^−^	>10^4^
			I^−^	1298

[a] *K*_a_ values calculated using Bindfit software using a 1 : 1 host–guest binding model. Sodium cation added as NaBAr^F^_4_. All anions added as their TBA salts. Global fit performed for all systems. Errors (±) are all <10 % unless specified. [b] Salt recombination.

Importantly, the introduction of one equivalent of NaBAr^F^_4_ salt to the [2]rotaxanes resulted in a significant enhancement of the bromide and iodide anion binding strength ca. 20‐fold for [2]rotaxanes **7** and **8**. This ion‐pair binding cooperativity effect can be attributed to favourable axle separated cation‐anion electrostatic interactions and macrocyclic preorganization induced in the presence of a co‐bound sodium cation.

In the case of the NaCl ion‐pair binding experiments the situation is more complex. During the course of ion‐pair binding experiments with salts possessing high lattice enthalpies, such as NaCl, a strongly competing exogenous ion‐pairing salt recombination equilibrium is present. In order to rationalize the differing behaviours of **6**,**7** and **8** it is useful to consider the % bound sodium to the [2]rotaxane hosts under these titration conditions which can be calculated though the previously determined *K*_a_(Na^+^) values. Since, [2]rotaxane **6** possesses the highest sodium cation association constant it has the highest % bound Na^+^, >75 % of the rotaxane series. However, the addition of TBACl to a Na^+^ pre‐complexed solution of [2]rotaxane **6** resulted in salt recombination as evidenced by spectral comparison to the free rotaxane. Rotaxanes **7** and **8** exhibit similar *K_a_* (Na^+^) values and therefore, comparable Na^+^ bound percentages of 41 % and 45 % respectively. The addition of TBACl to these solutions resulted in NaCl precipitation for [2]rotaxane **7** whilst careful spectral analysis confirmed the concomitant binding of axle‐separated NaCl ion‐pair to [2]rotaxane **8**. Although displaying a strong affinity for sodium, the inability of rotaxane **6** to bind the NaCl ion‐pair may be rationalized by weaker host‐anion interactions owing to lack of Lewis acidic HB NH donors in its macrocycle component. On the other hand, [2]rotaxane **7**, despite possessing additional NH donors courtesy of the isophthalamide motif in the macrocycle, still failed to concertedly bind a NaCl ion‐pair, which is presumably due to insufficient Na^+^ binding. Interestingly, [2]rotaxane **8**, which displays only a ca. 4 % increase in Na^+^‐bound‐rotaxane relative to **7** was able to simultaneously, and importantly cooperatively, bind a NaCl ion‐pair, impressively overcoming the high lattice enthalpy of the NaCl salt. However, in contrast to the heavier halides which exhibited up to a 20‐fold enhancement in the presence of sodium, only a modest enhancement in the chloride association constant from 543 M^−1^ to 646 M^−1^ was observed for [2]rotaxane **8**. This could be accredited to the higher lattice enthalpy of NaCl which competes with the receptor's ion‐pair complexation mode, attenuating binding enhancement behaviour. Crucially, this highlights the delicate interplay between the efficacy of both cation and anion binding properties in the ion‐pair binding capabilities of heteroditopic receptor systems.

### Preliminary solid‐liquid extraction studies

Following the promising ion‐pair binding results, the ability of the heteroditopic [2]rotaxanes to extract solid sodium halide salts into organic solvent media was also investigated. In a typical experiment, a 1 mM solution of each [2]rotaxane in CDCl_3_ was exposed to an excess (ca. 5‐fold) microcrystalline sample of solid NaX salt and sonicated for 20 min, after which the solution was filtered and its ^1^H NMR spectrum recorded.

Comparison of the pre‐ and post‐extraction ^1^H NMR spectra indicated all three [2]rotaxanes were able to successfully extract NaBr and NaI, with [2]rotaxanes **7** and **8** showing significant perturbations as compared to [2]rotaxane **6** (Figure 6c). Despite it not being possible to calculate % extraction efficiencies, comparison of the pre‐ and the post‐extraction ^1^H NMR spectra indicates the complexation induced shifts (CIS) of the macrocycle component amide signals H_b_ and H_c_ of [2]rotaxanes **7** and **8** is consistent with the enhanced NaBr over NaI affinity as reflected in the larger perturbations in chemical shift signals (Figure 6c). Taking account of the ^1^H NMR ion‐pair binding studies, as expected [2]rotaxanes **6** and **7** were incapable of extracting NaCl owing to the high lattice enthalpy of the alkali halide salt. However, despite ^1^H NMR evidence of solution NaCl ion‐pair binding by [2]rotaxane **8**, the MIM host was found to be unable to extract solid NaCl which may be rationalized by the relatively modest solution phase enhancement in chloride binding strength observed in the presence of co‐bound sodium cation.

## Conclusions

In conclusion, a series of novel squaramide‐based heteroditopic [2]rotaxanes were synthesized for ion‐pair recognition investigation *via* an unprecedented sodium cation template coordination of the Lewis basic squaramide carbonyls for MIM construction. Extensive cation, anion and ion‐pair ^1^H NMR titration studies showed that the bromide and iodide recognition capabilities of these heteroditopic MIM receptors were significantly enhanced in the presence of rotaxane axle squaramide‐macrocycle intercomponent co‐bound sodium cations, ca. 20‐fold with [2]rotaxanes **7** and **8**, highlighting the advantages of mechanical bond positive cooperativity in ion‐pair recognition. A relatively smaller enhancement in halide binding strength was observed with [2]rotaxane **6** which may be ascribed to lack of amide NH donors in the interlocked structure's macrocyclic component. Notably, all receptors exhibited impressive bromide selectivity in the presence of co‐bound sodium cation. Of the three rotaxanes studied, only [2]rotaxane **8** was capable of binding the NaCl ion‐pair, underpinning the importance of strong concomitant cation and anion association in overcoming competing lattice enthalpies to achieve ion‐pair recognition. Furthermore, preliminary solid sodium halide salt‐liquid extraction studies demonstrated that all three MIM receptor systems were able to successfully extract NaBr and NaI into organic media, consistent with their solution phase ion‐pair recognition properties. Importantly, this work highlights the untapped potential of exploiting the unique ambidentate nature of the squaramide motif, containing both preorganized Lewis acidic HB donors for anion binding and in particular Lewis basic carbonyl moieties for alkali metal cation template directed MIM synthesis, in future mechanical bond heteroditopic receptor design.

## Supporting Information

The authors have cited additional references within the Supporting Information.[^55^, ^56^, ^57^, ^58^, ^59^, ^60^]

## Conflict of interest

The authors declare no conflict of interest.

1

## Supporting information

As a service to our authors and readers, this journal provides supporting information supplied by the authors. Such materials are peer reviewed and may be re‐organized for online delivery, but are not copy‐edited or typeset. Technical support issues arising from supporting information (other than missing files) should be addressed to the authors.

Supporting Information

## Data Availability

The data that support the findings of this study are available in the supplementary material of this article.
