# Supplementary material for: Squaramide‐Based Heteroditopic [2]Rotaxanes for Sodium Halide Ion‐Pair Recognition
Source: Chemistry. 2023 Jul 26;29(49):e202301446. doi: 10.1002/chem.202301446 (PMC10946609; doi:10.1002/chem.202301446)
Supplement: Supplementary file 1 — Supporting Information [file CHEM-29-0-s001.pdf]

Chemistry–A European Journal

Supporting Information

Squaramide-Based Heteroditopic [2]Rotaxanes for Sodium Halide Ion-Pair Recognition

Arya Arun, Andrew Docker, Hui Min Tay, and Paul D. Beer*

Table of Contents

Synthesis and characterisation 3

Materials and methods 3

General Procedure 1 3

General Procedure 2 7

^{1}H NMR pseudorotaxane studies 22

^{1}H NMR binding studies 28

General procedure 28

Anion titrations 28

Cation titrations 33

Ion-pair titrations 35

Binding Isotherms 40

Solid-liquid extraction experiments 43

References 46

Synthesis and characterisation

Materials and methods

All solvents and reagents were purchased from commercial suppliers and used as received unless otherwise stated. Dry solvents were obtained by purging with nitrogen and then passing through an MBraun MPSP-800 column. H2O was de-ionized and micro filtered using a Milli-Q ® Millipore machine. Column chromatography was carried out on Merck® silica gel 60 under a positive pressure of nitrogen. Routine NMR spectra were recorded on either a Varian Mercury 300, a Bruker AVIII 400 or a Bruker AVIII 500 spectrometer with ^{1}H NMR titrations recorded on a Bruker AVIII 500 spectrometer. TBA salts were stored in a vacuum desiccator containing phosphorus pentoxide prior to use. Where mixtures of solvents were used, ratios are reported by volume. Chemical shifts are quoted in parts per million relative to the residual solvent peak. Mass spectra were recorded on a Bruker µTOF spectrometer. Triethylamine was distilled from and stored over potassium hydroxide. Stopper alkyne (**5**)[55], Tris[(1-benzyl-1H-1,2,3- triazol-4-yl)methyl]amine (TBTA),[56] di-ethylene glycol-based amines (**1,3**),[57] and Macrocycles (**A-C**)[58-60] were prepared according to previous literature reports.

General procedure 1*: Synthesis of axle precursors*

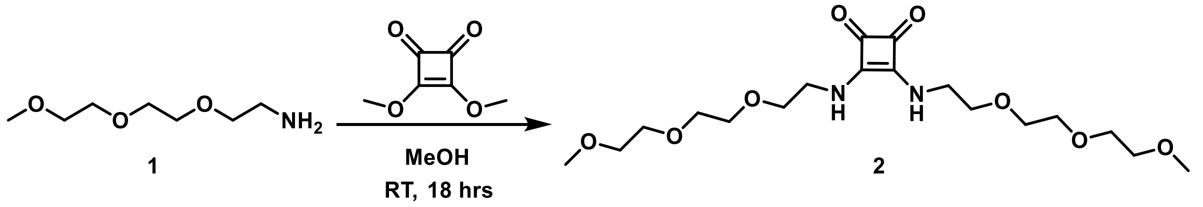

Squaramide tetraethylene glycol-based axle precursor (2). Squarate ester (30 mg, 0.211 mmol) was dissolved in methanol (1 mL). A solution of tri(ethyleneglycol) functionalised amine **1** (72.36 mg, 0.443 mmol)) in methanol (0.5 mL) was then added to it dropwise. The resulting mixture was left to stir at RT overnight. After removing the solvent under vacuum, purification by column chromatography afforded the target squaramide as a yellow oil (49 mgs,57%) yield.

^{1}H NMR (500 MHz, $CDCl_3$) δ = 6.70, 3.87, 3.86, 3.85, 3.83, 3.67, 3.66, 3.66, 3.65, 3.65, 3.65, 3.64, 3.64, 3.64, 3.63, 3.63, 3.62, 3.62, 3.62, 3.61, 3.61, 3.61, 3.60, 3.60, 3.59, 3.59, 3.58, 3.58, 3.58, 3.39.

^{13}C NMR (151 MHz, $CDCl_3$) δ = 206.9, 183.6, 168.1, 77.2, 77.0, 76.8, 71.8, 70.7, 70.4, 70.0, 69.9, 58.9, 43.9.

HRMS (ESI +ve) m/z: 419.1793 ($[M+Na]^+$, $C_{20}H_{24}O_3N_6Na$ requires 419.1802).

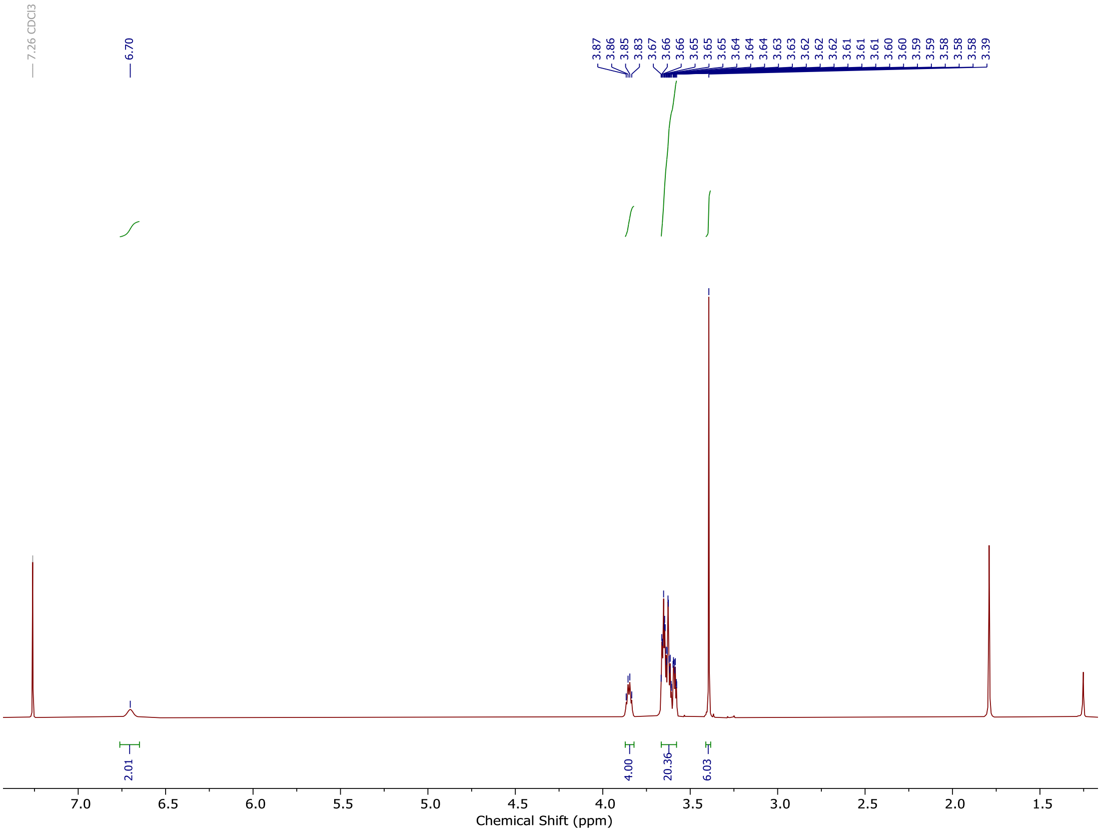

*Figure S1. ^{1}H-NMR spectrum of TEG axle precursor **2** (500 MHz, $CDCl_3$, 298 K)*

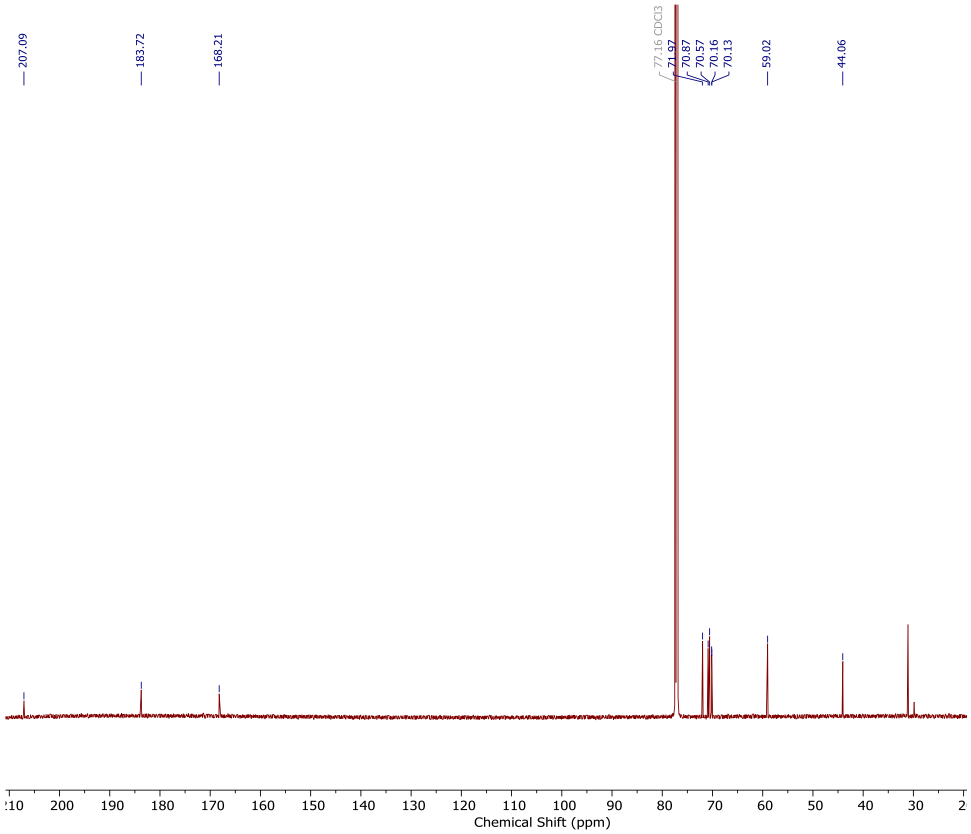

*Figure S2. ^{13}C-NMR spectrum of TEG axle precursor **2** (500 MHz, $CDCl_3$, 298 K)*

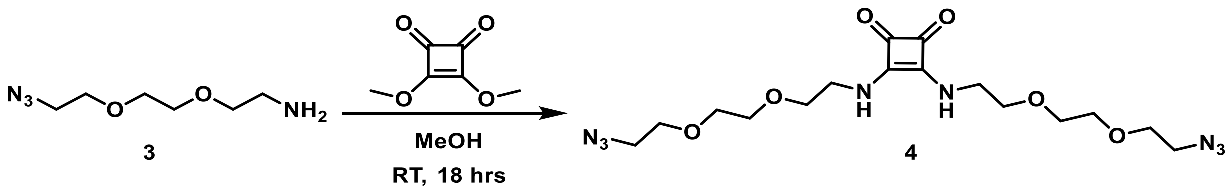

Squaramide diethylene glycol-based bis-azide (4). Squarate ester (75 mg, 0.53 mmol) was dissolved in methanol (1 mL). A solution of tri(ethyleneglycol) functionalised amine **3** (193.07 mg, 1.1083 mmol)) in methanol (1 mL) was then added to it dropwise. The resulting mixture was left to stir at RT overnight. After removing the solvent under vacuum, purification by column chromatography afforded the target squaramide as a yellow oil (211 mgs,94%).

^{1}H NMR (600 MHz, $CDCl_3$) δ = 6.43, 6.42, 6.41, 3.84, 3.84, 3.83, 3.82, 3.69, 3.68, 3.68, 3.67, 3.66, 3.65, 3.65, 3.41, 3.40, 3.40.

^{13}C NMR (151 MHz, $CDCl_3$) δ = 207.0, 183.4, 168.1, 77.2, 77.0, 76.8, 70.6, 70.5, 70.5, 70.2, 70.1, 70.0, 69.9, 50.7, 44.0, 40.9, 39.3.

HRMS (ESI +ve) m/z: 419.1793 ($[M+Na]^+$, $C_{20}H_{24}O_3N_6Na$ requires 419.1802).

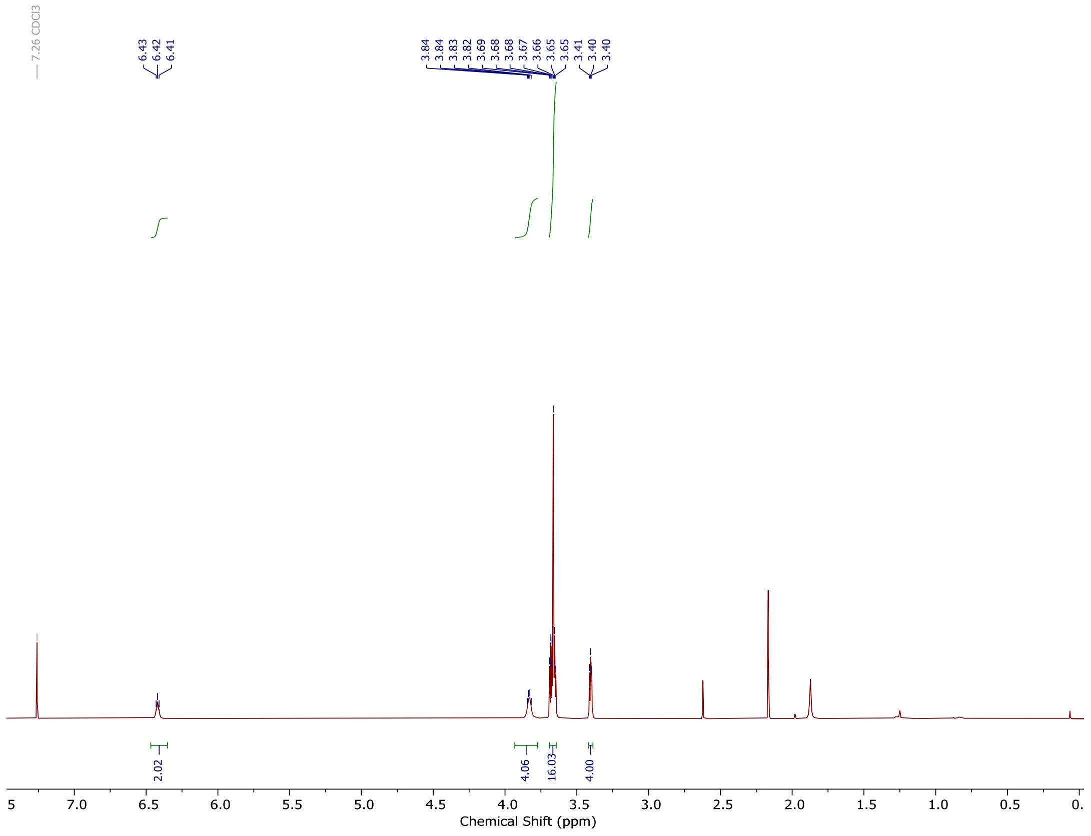

*Figure S3. ^{1}H-NMR spectrum of TEG-azide axle precursor **4** (500 MHz, $CDCl_3$, 298 K)*

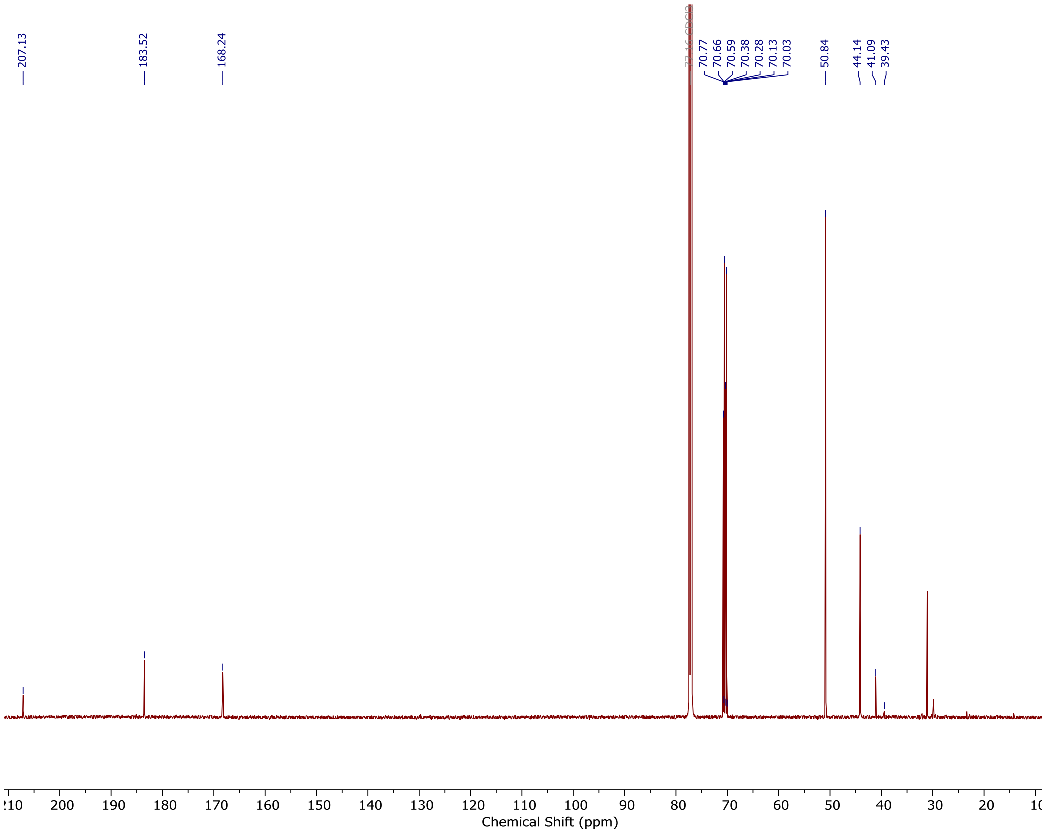

*Figure S4. ^{13}C-NMR spectrum of TEG-azide axle precursor **4** (500 MHz, $CDCl_3$, 298 K)*

General procedure 2*: Alkali metal-templated synthesis of [2]rotaxanes*

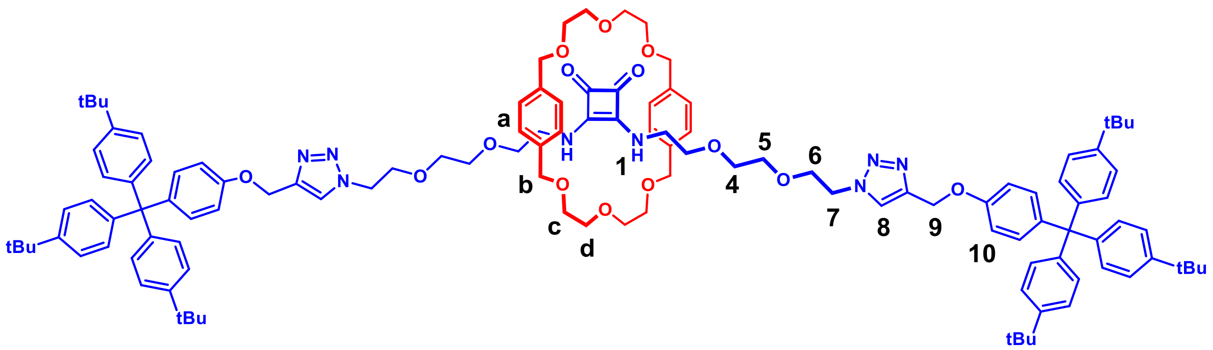

[2]rotaxane (6). Macrocycle **A** (20.0 mg, 0.048 mmol) and $NaBAr_4^F$ (42.56 mg, 0.048 mmol) were dissolved in dry, degassed CH_2Cl_2 (0.5 mL) and stirred for 30 minutes at room temperature. A solution of bis-azide **4** (46.54 mg, 0.048 mmol) in CH_2Cl_2 (0.5 mL) was added and the mixture stirred for a further 30 minutes. A solution of terphenyl stopper alkyne **5** (65.16 mg, 0.060 mmol) in CH_2Cl_2 (0.7 mL) was added, followed by a dropwise addition of a premixed solution of $[Cu(CH_3CN)_4]PF_6$ (4.47 mg, 0.012 mmol) and TBTA (6.37 mg, 0.012 mmol) in CH_2Cl_2 (0.7 mL). The reaction mixture was stirred at room temperature in for 48 hours, then was diluted with CH_2Cl_2 (40 mL). The organic layer was washed with EDTA/NH_4OH (2 × 25 mL) and H_2O (2 × 25 mL), dried over $MgSO_4$, filtered and concentrated under vacuum. The crude was purified by preparative TLC in 70:25:5 CH_2Cl_2/EtOAc/MeOH to afford [2]rotaxane **6** as a white solid (13 mg, 14%).

^{1}H NMR (400 MHz, $CDCl_3$) δ = 7.76, 7.23, 7.21, 7.13, 7.11, 7.09, 7.07, 7.05, 6.86, 6.84, 6.17, 5.15, 4.48, 4.32, 3.82, 3.57, 3.49, 3.38, 3.21, 3.08, 1.29.

HRMS (ESI +ve) m/z: 1951.1102 ($[M+Na]^+$, $C_{120}H_{150}N_8O_{14}$ requires 1951.1197).

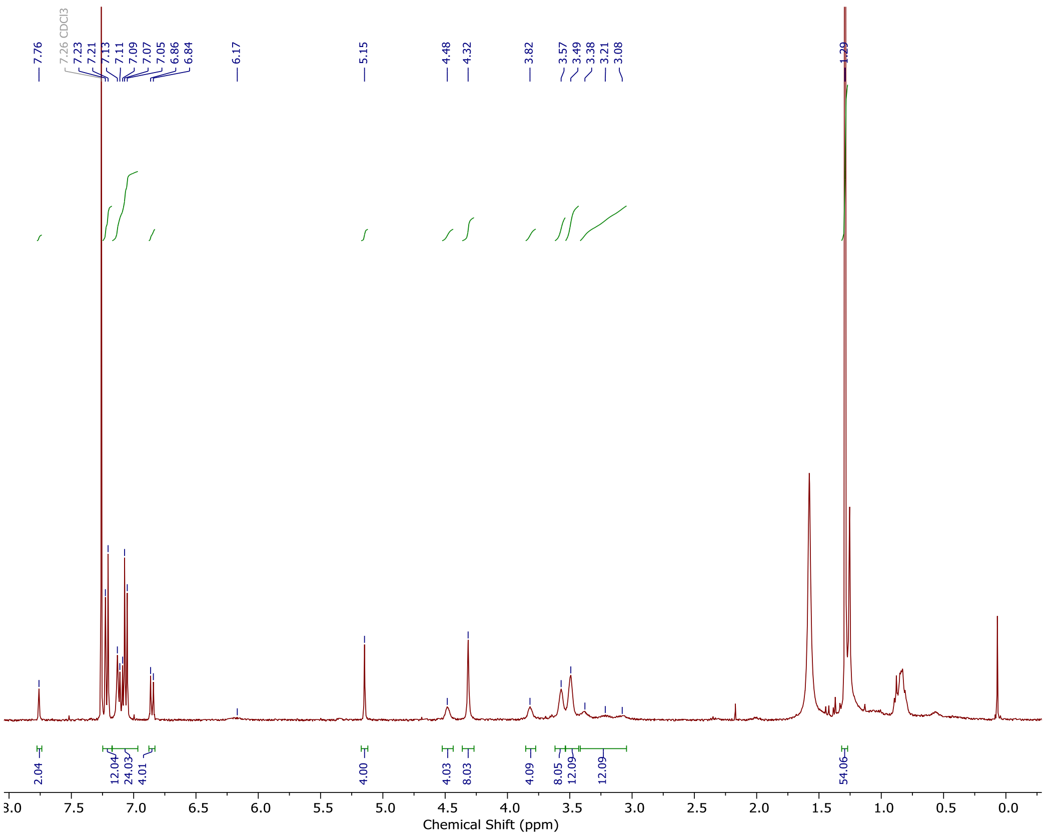

*Figure S5. ^{1}H-NMR spectrum of [2]rotaxane **6** (500 MHz, $CDCl_3$, 298 K)*

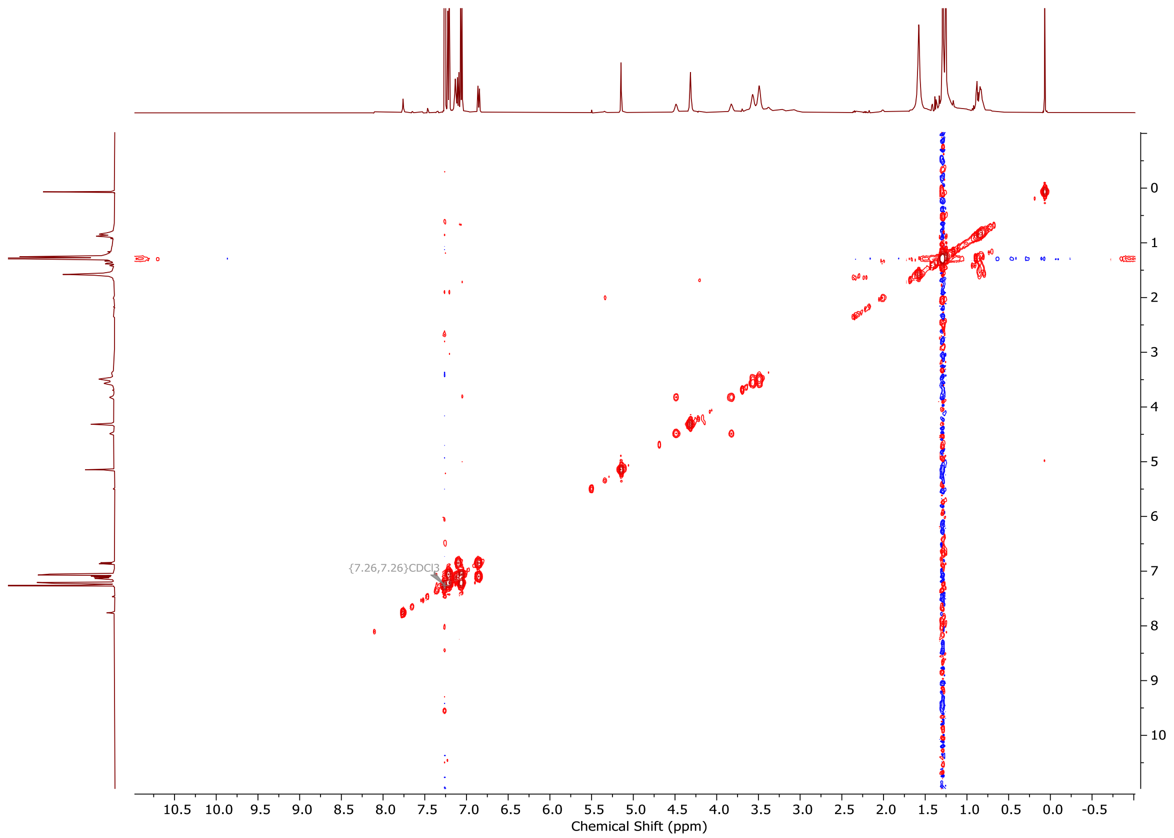

*Figure S6. ^{1}H-^{1}H COSY NMR spectrum of [2]rotaxane **6** (500 MHz, $CDCl_3$, 298 K)*

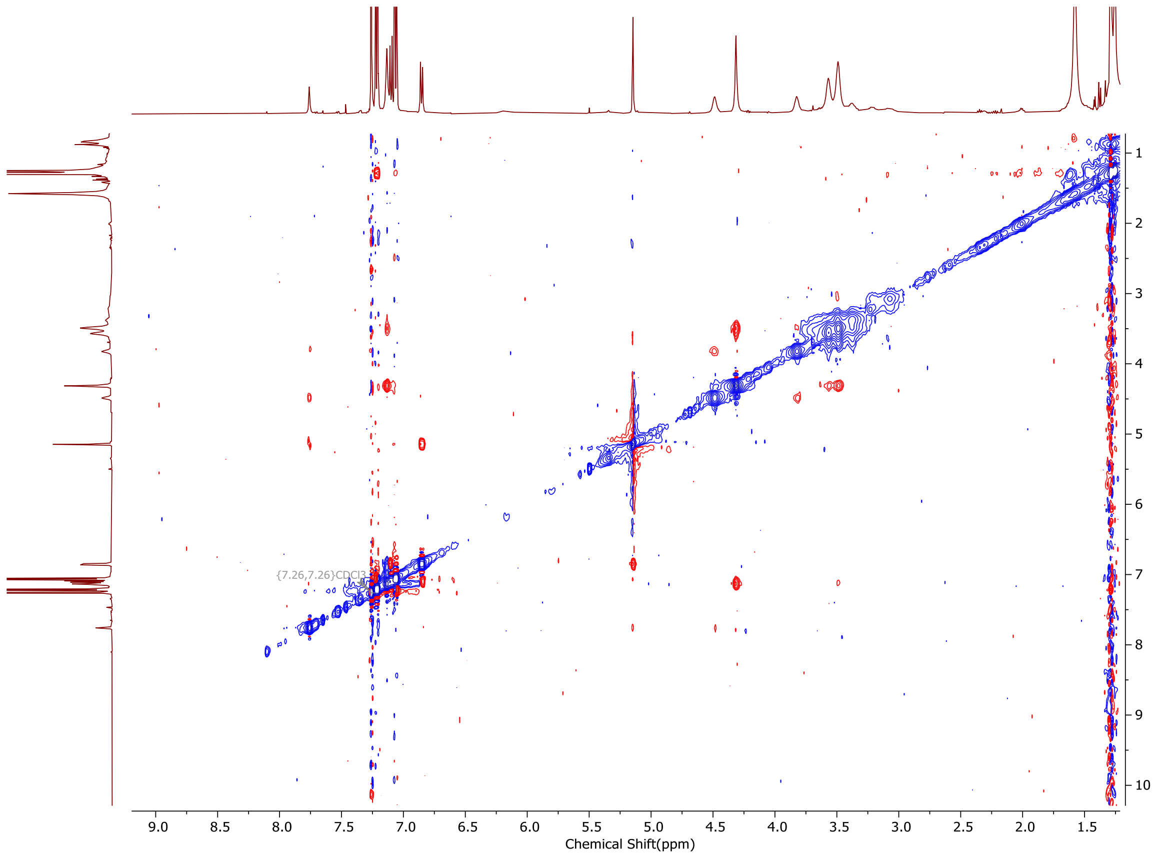

*Figure S7. ^{1}H-^{1}H ROESY NMR spectrum of [2]rotaxane **6** (500 MHz, $CDCl_3$, 298 K)*

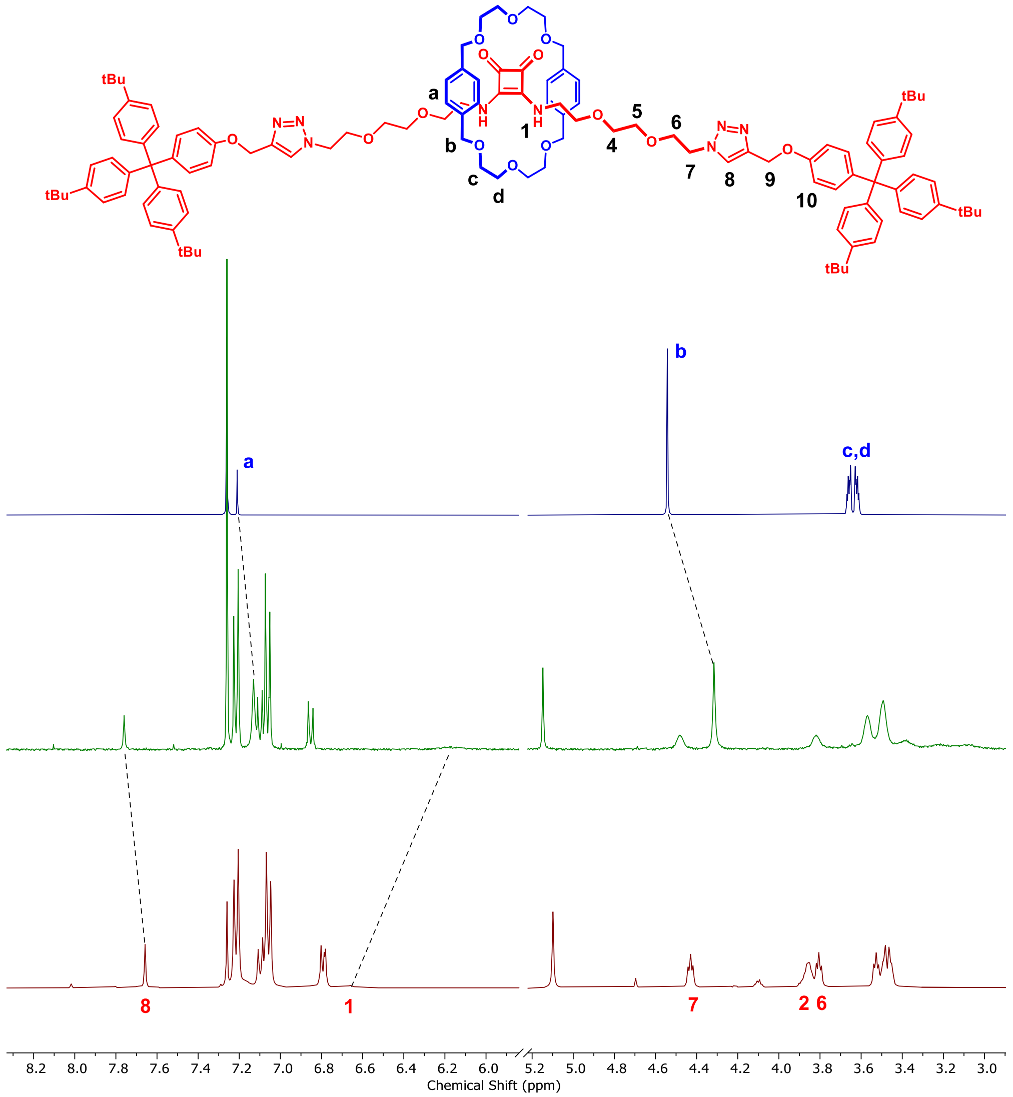

*Figure S8. Stacked ^{1}H NMR spectra of Macrocycle **A** (top) and [2]rotaxane **6** (middle) and axle **6a** (bottom) (500 MHz, $CDCl_3$, 298 K).*

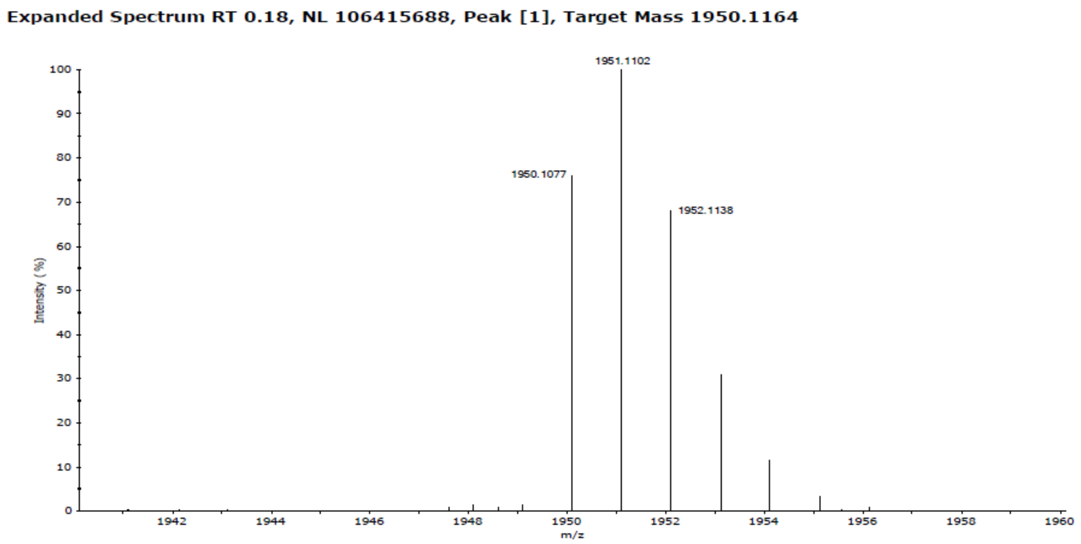

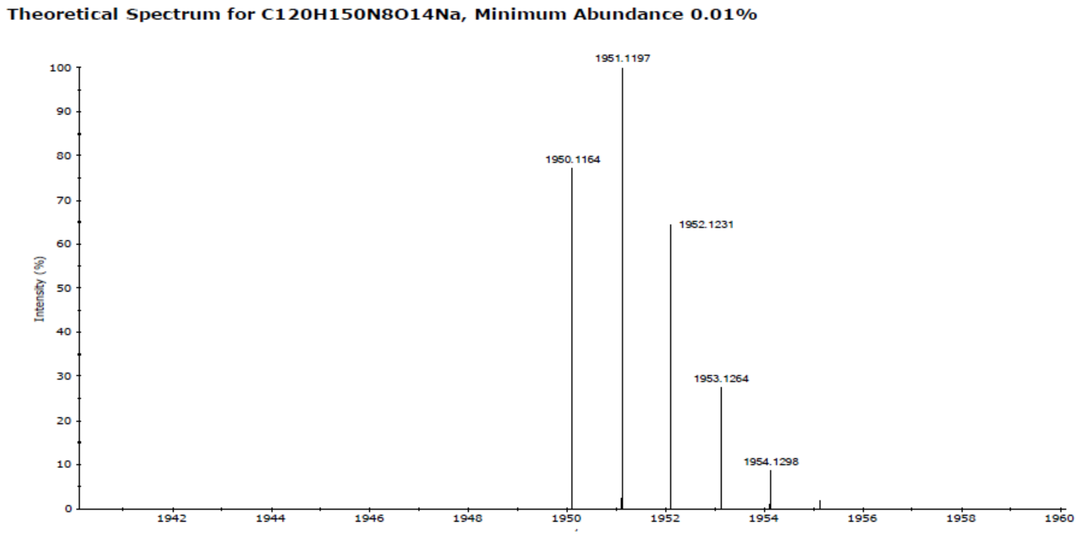

Figure S9. High-resolution mass spectrum (ESI +ve) of ***[2]rotaxane 6*** *(top: expanded experimental; bottom: theoretical).*

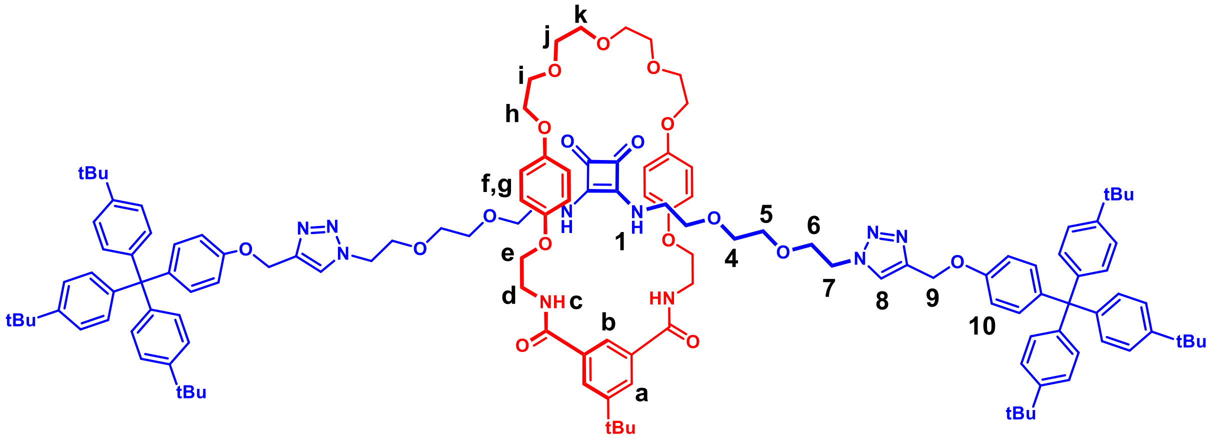

[2]rotaxane (7). Macrocycle **B** (13.0 mg, 0.019 mmol) and $NaBAr_4^F$ (17.70 mg, 0.019 mmol) were dissolved in dry, degassed CH_2Cl_2 (0.5 mL) and stirred for 30 minutes at room temperature. A solution of bis-azide **4** (8.52 mg, 0.019 mmol) in CH_2Cl_2 (0.5 mL) was added and the mixture stirred for a further 30 minutes. A solution of terphenyl stopper alkyne **5** (13.554 mg, 0.0497 mmol) in CH_2Cl_2 (0.5 mL) was added, followed by a dropwise addition of a premixed solution of $[Cu(CH_3CN)_4]PF_6$ (3.72 mg, 0.01 mmol) and TBTA (5.30 mg, 0.01 mmol) in CH_2Cl_2 (0.5 mL). The reaction mixture was stirred at room temperature in for 48 hours, then was diluted with CH_2Cl_2 (40 mL). The organic layer was washed with EDTA/NH_4OH (2 × 25 mL) and H_2O (2 × 25 mL), dried over $MgSO_4$, filtered and concentrated under vacuum. The crude was purified by preparative TLC in 70:25:5 CH_2Cl_2/EtOAc/MeOH to afford [2]rotaxane **7** as a white solid (5.6 mg, 13%).

^{1}H NMR (500 MHz, $CDCl_3$) δ = 8.30, 8.26, 7.85, 7.84, 7.83, 7.70, 7.26, 7.23, 7.21, 7.11, 7.10, 7.10, 7.07, 7.06, 7.06, 6.85, 6.83, 6.68, 6.66, 6.64, 6.63, 5.12, 4.49, 4.48, 4.47, 4.14, 4.13, 4.13, 3.91, 3.90, 3.90, 3.89, 3.85, 3.84, 3.83, 3.72, 3.72, 3.71, 3.65, 3.64, 3.63, 3.60, 3.59, 3.58, 3.56, 3.55, 3.54, 3.53, 3.46, 3.45, 3.45, 3.43, 2.58, 1.29, 1.25.

^{13}C NMR (151 MHz, $CDCl_3$) δ = 182.8, 167.8, 167.6, 156.3, 152.5, 152.5, 152.4, 148.5, 144.3, 144.2, 140.5, 133.9, 132.5, 130.9, 128.9, 124.4, 124.2, 124.0, 123.9, 121.3, 115.4, 115.2, 113.3, 77.4, 77.2, 76.9, 70.8, 70.7, 70.6, 70.3, 70.3, 70.2, 69.5, 67.5, 66.3, 63.2, 62.1, 50.4, 43.8, 39.5, 35.2, 34.4, 31.5, 31.4.

HRMS (ESI +ve) m/z: 2163.2295 ($[M+H]^+$, $C_{132}H_{164}N_{10}O_{17}$ requires 2163.2382).

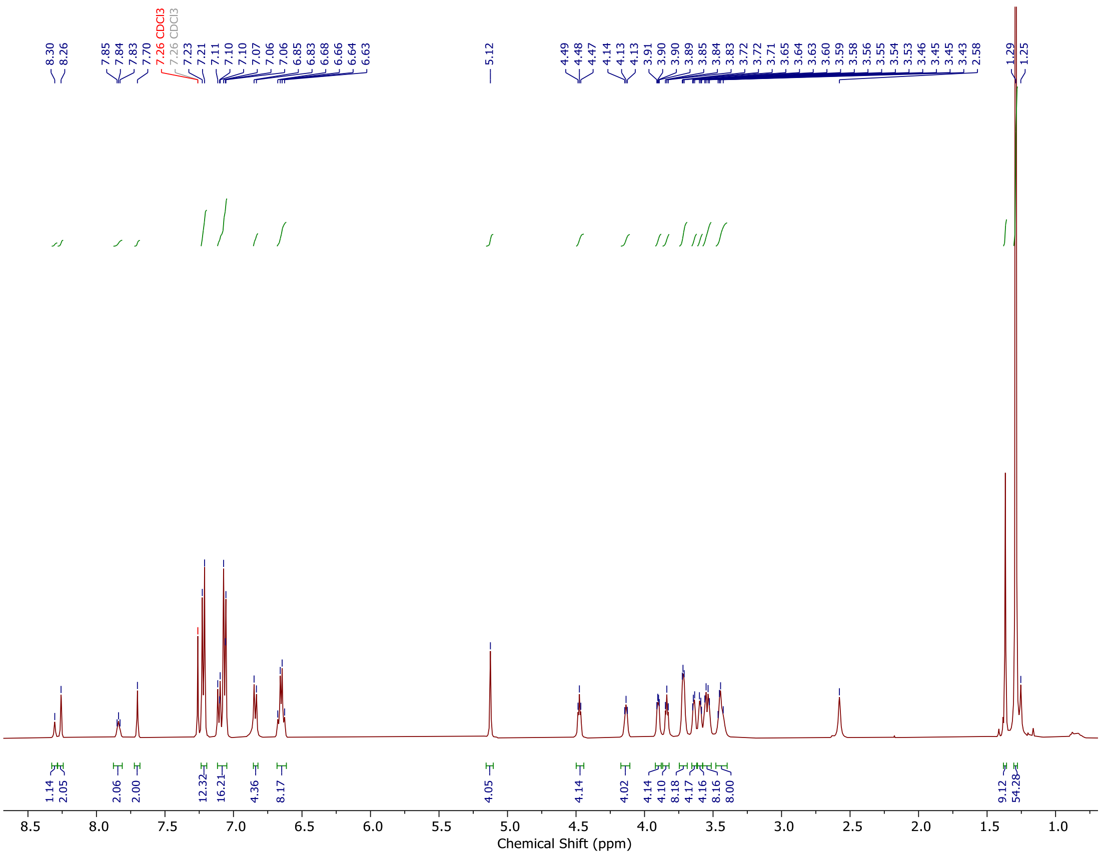

*Figure S10. ^{1}H-NMR spectrum of [2]rotaxane **7** (500 MHz, $CDCl_3$, 298 K)*

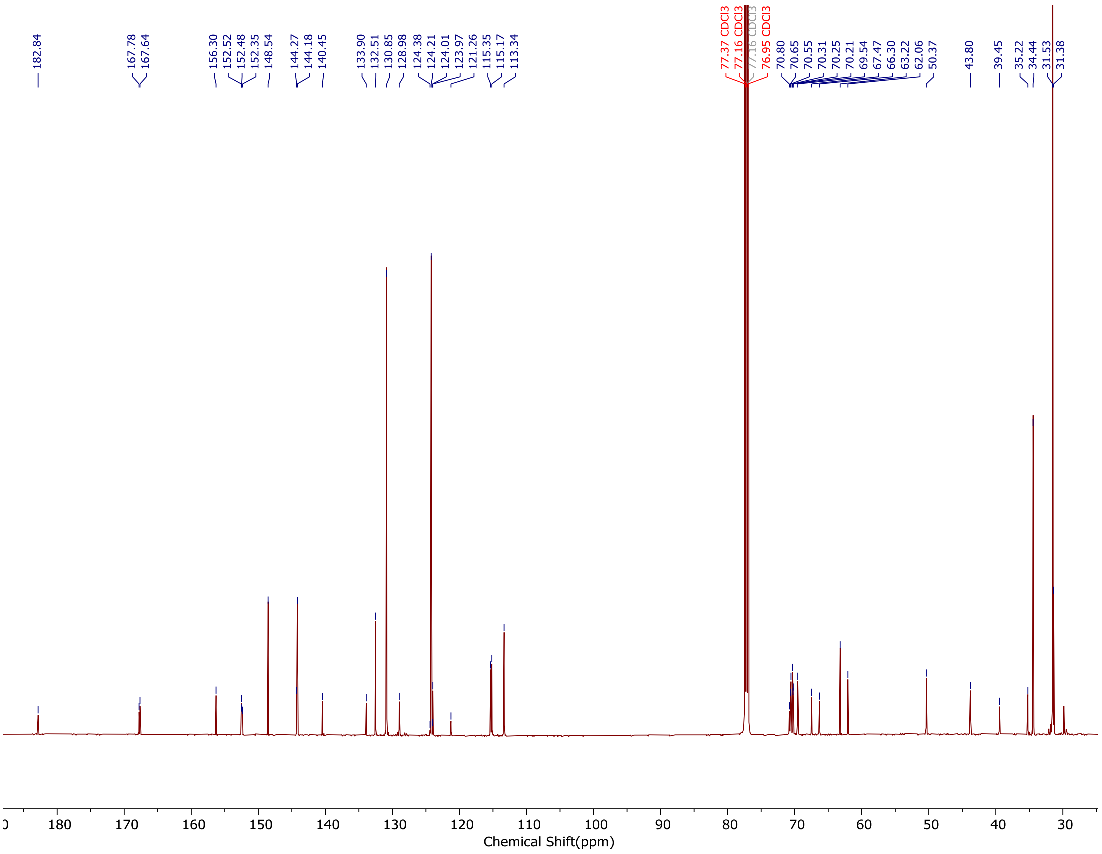

*Figure S11. ^{13}C-NMR spectrum of [2]rotaxane **7** (600 MHz, $CDCl_3$, 298 K)*

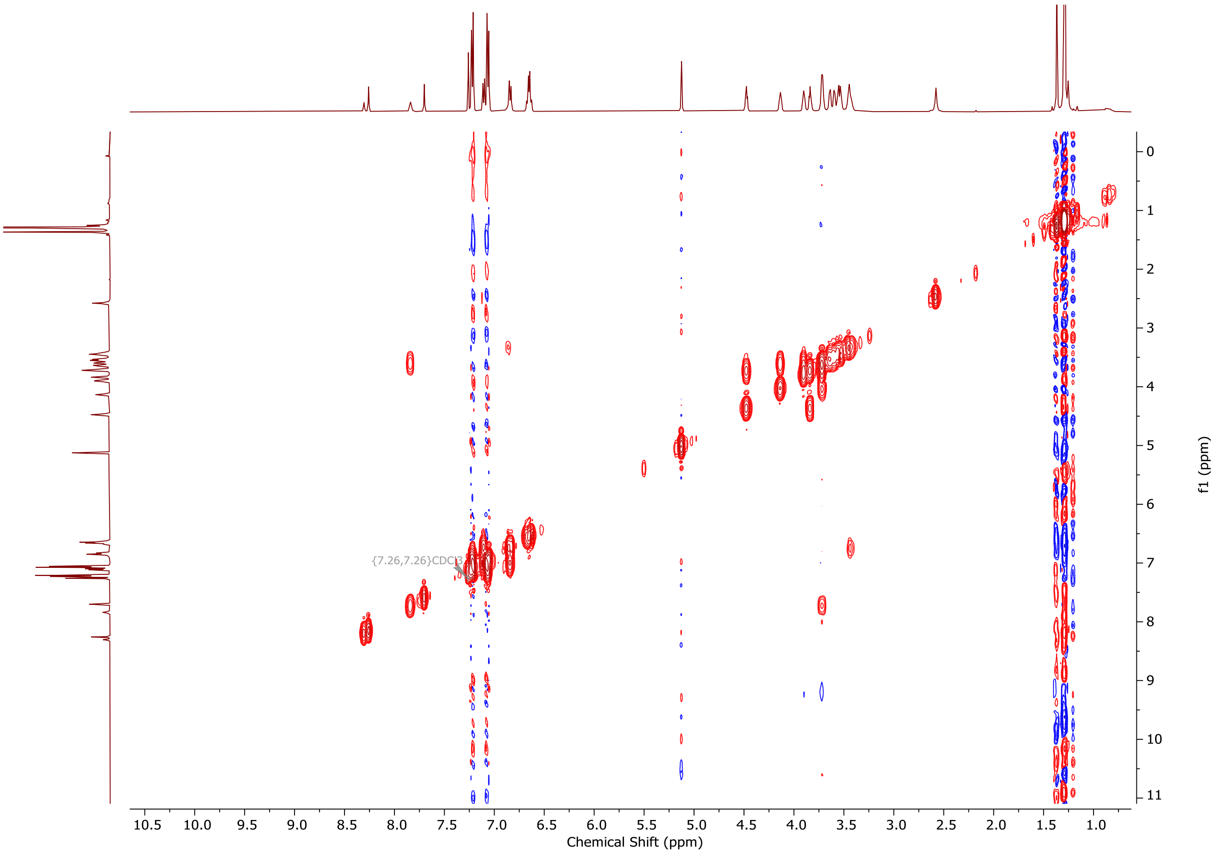

*Figure S12. ^{1}H-^{1}H COSY NMR spectrum of [2]rotaxane **7** (500 MHz, $CDCl_3$, 298 K)*

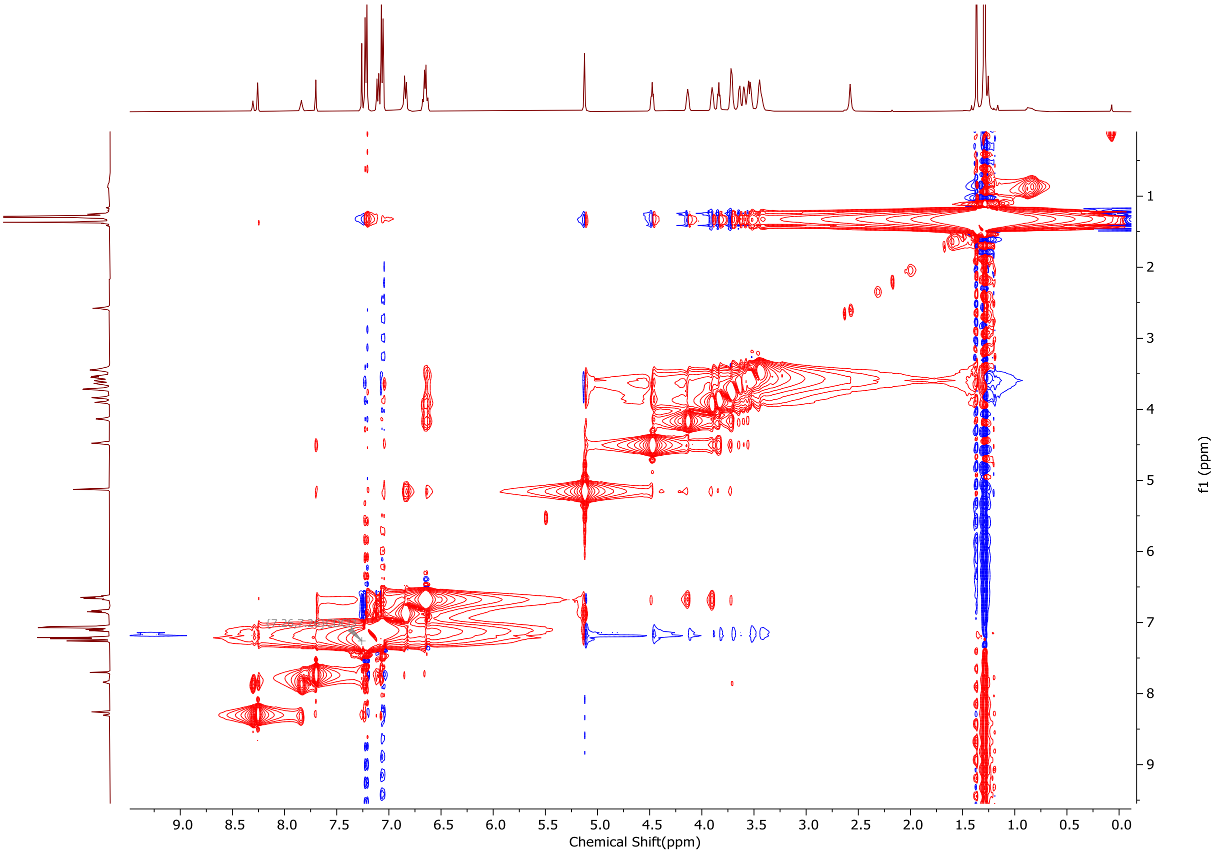

*Figure S13. ^{1}H-^{1}H ROESY NMR spectrum of [2]rotaxane **7** (500 MHz, $CDCl_3$, 298 K)*

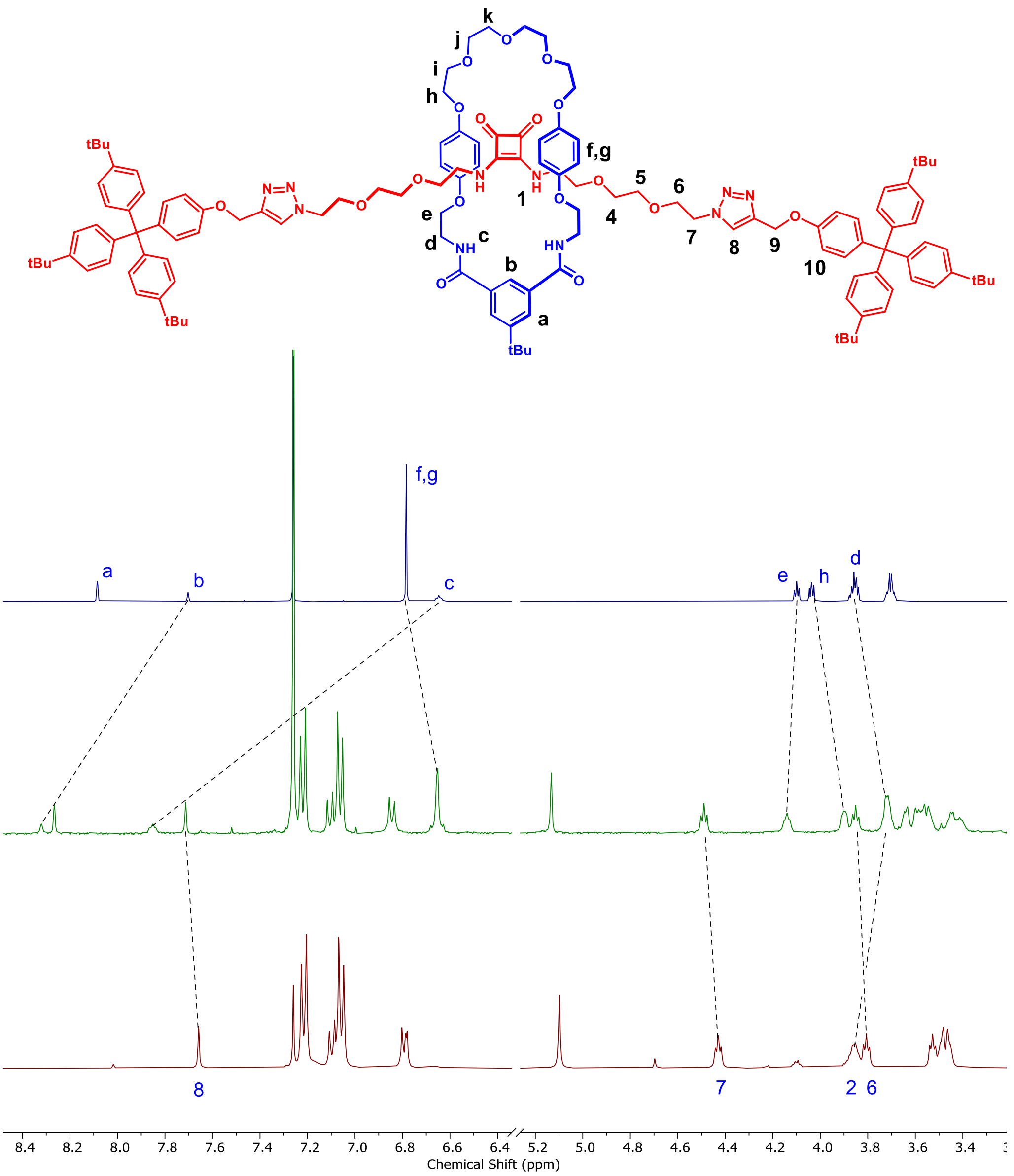

*Figure S14. Stacked ^{1}H NMR spectra of Macrocycle **B** (top) and [2]rotaxane **7** (middle) and axle **7a** (bottom) (500 MHz, $CDCl_3$, 298 K).*

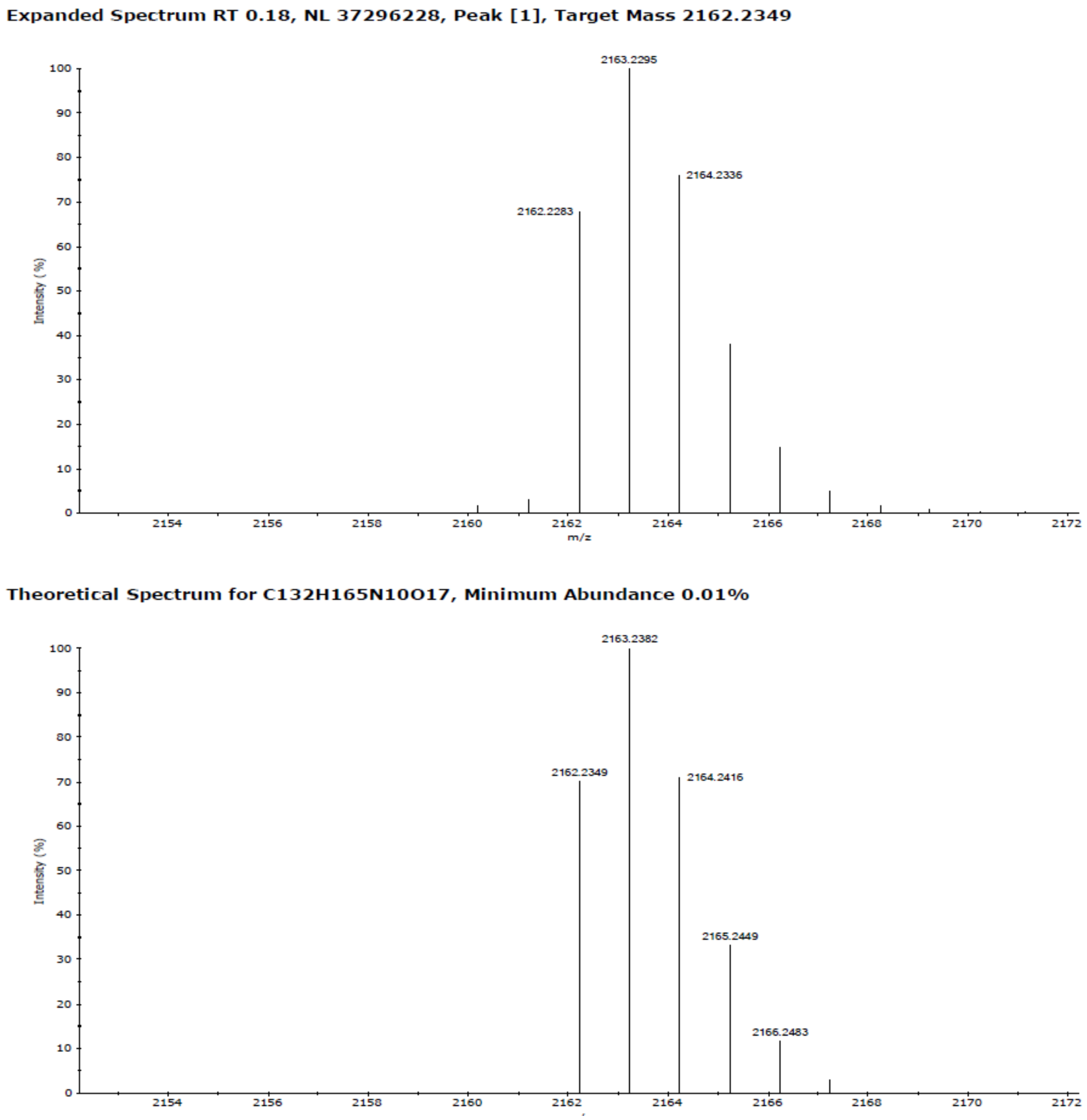

*Figure S15. High-resolution mass spectrum (ESI +ve) of **[2]rotaxane 7** (top: expanded experimental; bottom: theoretical).*

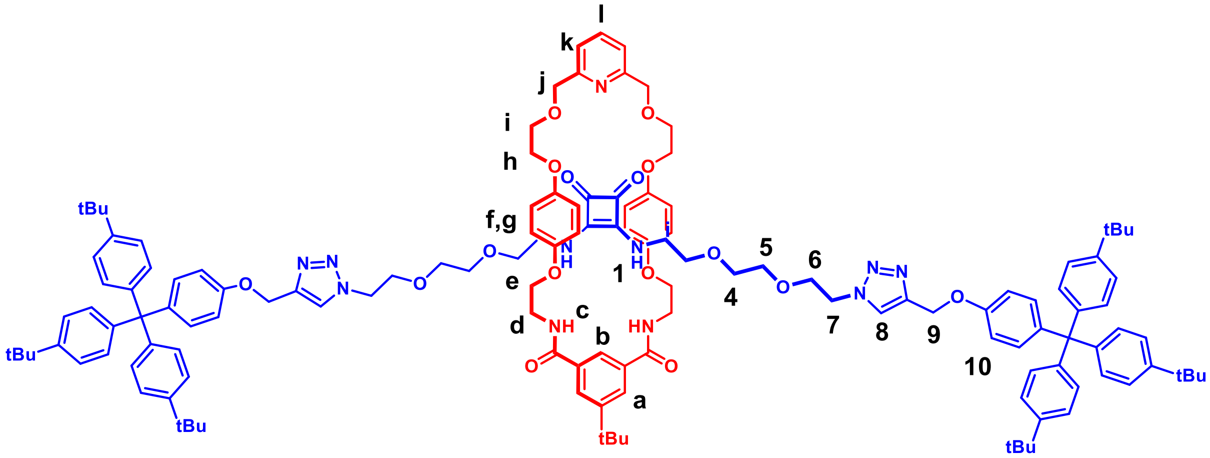

[2]rotaxane (8). Macrocycle **C** (13.5 mg, 0.019 mmol) and $NaBAr_4^F$ (17.50mg, 0.019 mmol) were dissolved in dry, degassed CH_2Cl_2 (0.5 mL) and stirred for 30 minutes at room temperature. A solution of bis-azide **4** (8.42 mg, 0.019 mmol) in CH_2Cl_2 (0.5 mL) was added and the mixture stirred for a further 30 minutes. A solution of terphenyl stopper alkyne **5** (26.79 mg, 0.019 mmol) in CH_2Cl_2 (0.5 mL) was added, followed by a dropwise addition of a premixed solution of $[Cu(CH_3CN)_4]PF_6$ (3.68 mg, 0.010 mmol) and TBTA (5.24 mg, 0.010 mmol) in CH_2Cl_2 (0.5 mL). The reaction mixture was stirred at room temperature in for 48 hours, then was diluted with CH_2Cl_2 (40 mL). The organic layer was washed with EDTA/NH_4OH (2 × 25 mL) and H_2O (2 × 25 mL), dried over $MgSO_4$, filtered and concentrated under vacuum. The crude was purified by preparative TLC in 70:25:5 CH_2Cl_2/EtOAc/MeOH to afford [2]rotaxane **8** as a white solid (8.2 mg, 19%).

^{1}H NMR (400 MHz, $CDCl_3$) δ = 8.35, 8.24, 7.84, 7.67, 7.66, 7.63, 7.23, 7.21, 7.11, 7.09, 7.08, 7.05, 6.99, 6.84, 6.82, 6.71, 6.69, 6.66, 6.64, 5.10, 4.64, 4.38, 4.09, 4.02, 3.87, 3.74, 3.73, 3.69, 3.48, 3.44, 3.40, 3.38, 1.36, 1.29.

^{13}C NMR (151 MHz, $CDCl_3$) δ = 182.9, 167.8, 167.7, 157.1, 156.3, 152.9, 152.3, 152.3, 148.5, 144.2, 144.2, 140.4, 138.1, 133.9, 132.5, 131.0, 130.8, 130.6, 128.9, 124.4, 124.2, 124.0, 123.9, 121.8, 121.4, 115.5, 115.5, 113.3, 77.4, 77.2, 76.9, 73.9, 70.7, 70.4, 70.3, 70.0, 69.4, 68.3, 66.6, 63.2, 62.0, 50.3, 43.8, 39.6, 35.2, 34.4, 31.5, 31.4, 29.8, 23.3, 22.8, 14.3, 14.3, 1.2.

HRMS (ESI +ve) m/z: 2218.2129 ($[M+Na]^+$, $C_{135}H_{163}N_{11}O_{16}$ requires 2218.2205).

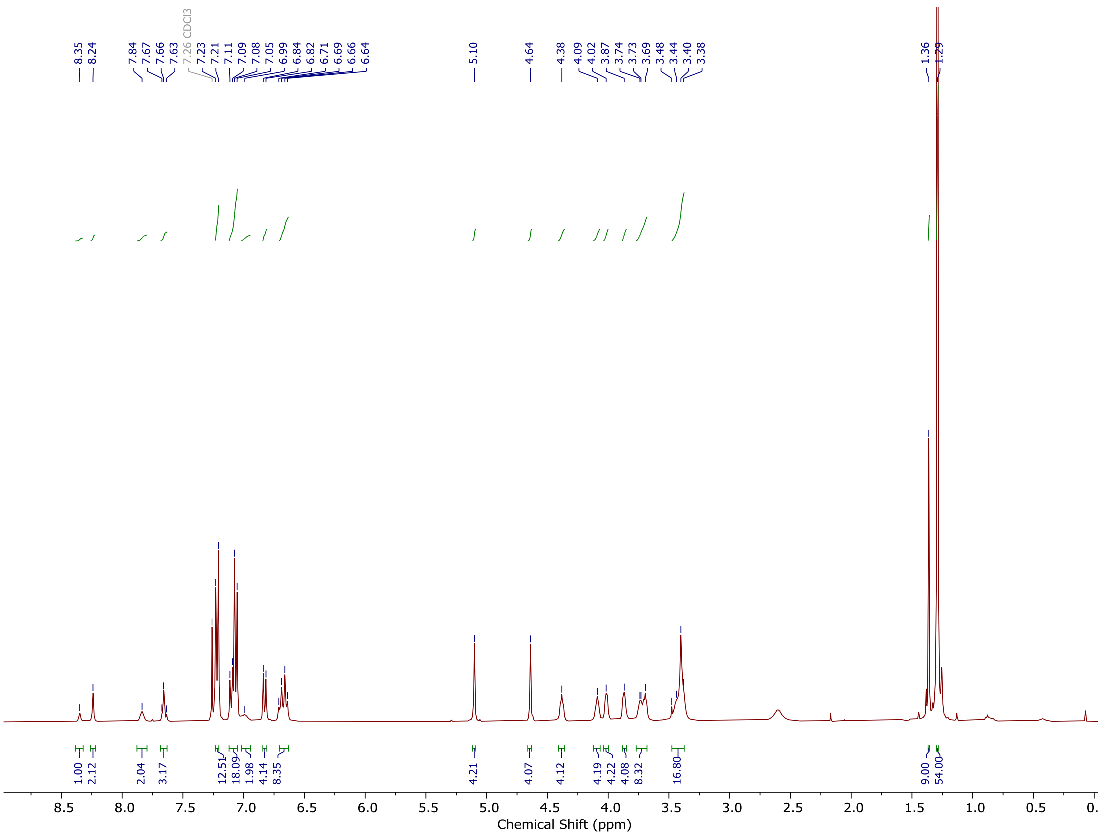

*Figure S16. ^{1}H-NMR spectrum of [2]rotaxane **8** (500 MHz, $CDCl_3$, 298 K)*

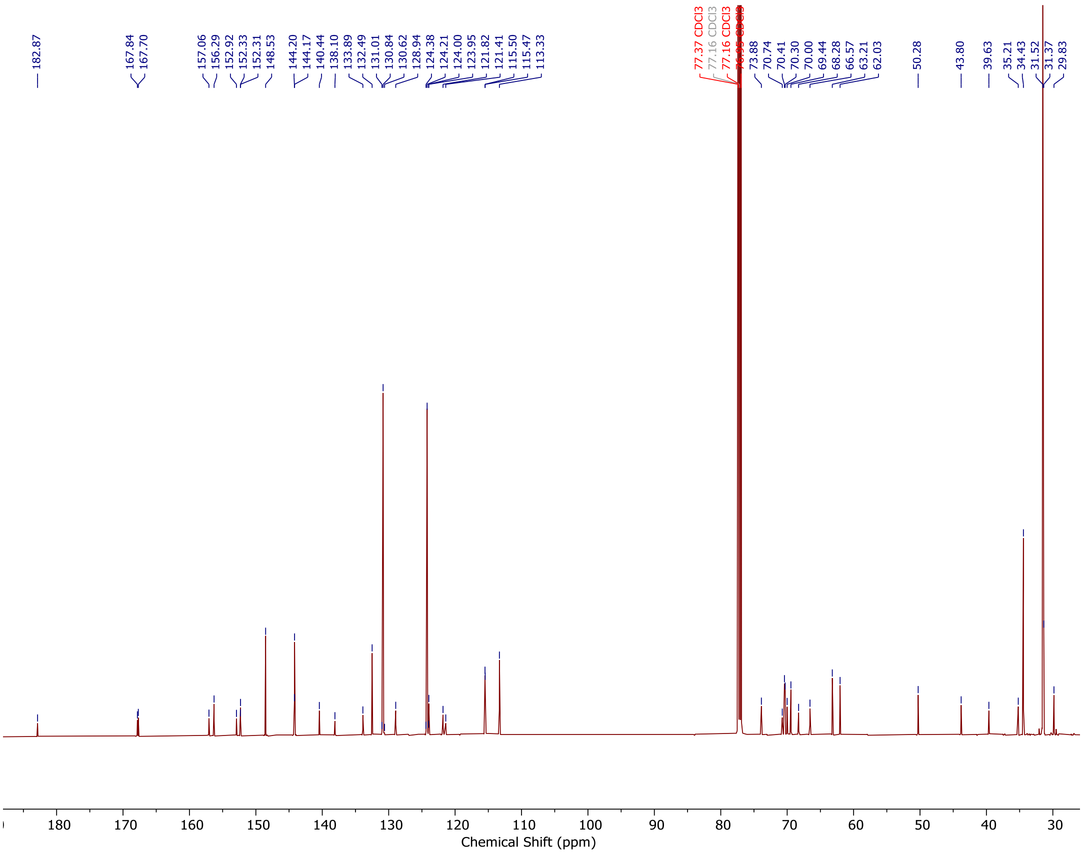

*Figure S17. ^{13}C-NMR spectrum of [2]rotaxane **8** (600 MHz, $CDCl_3$, 298 K)*

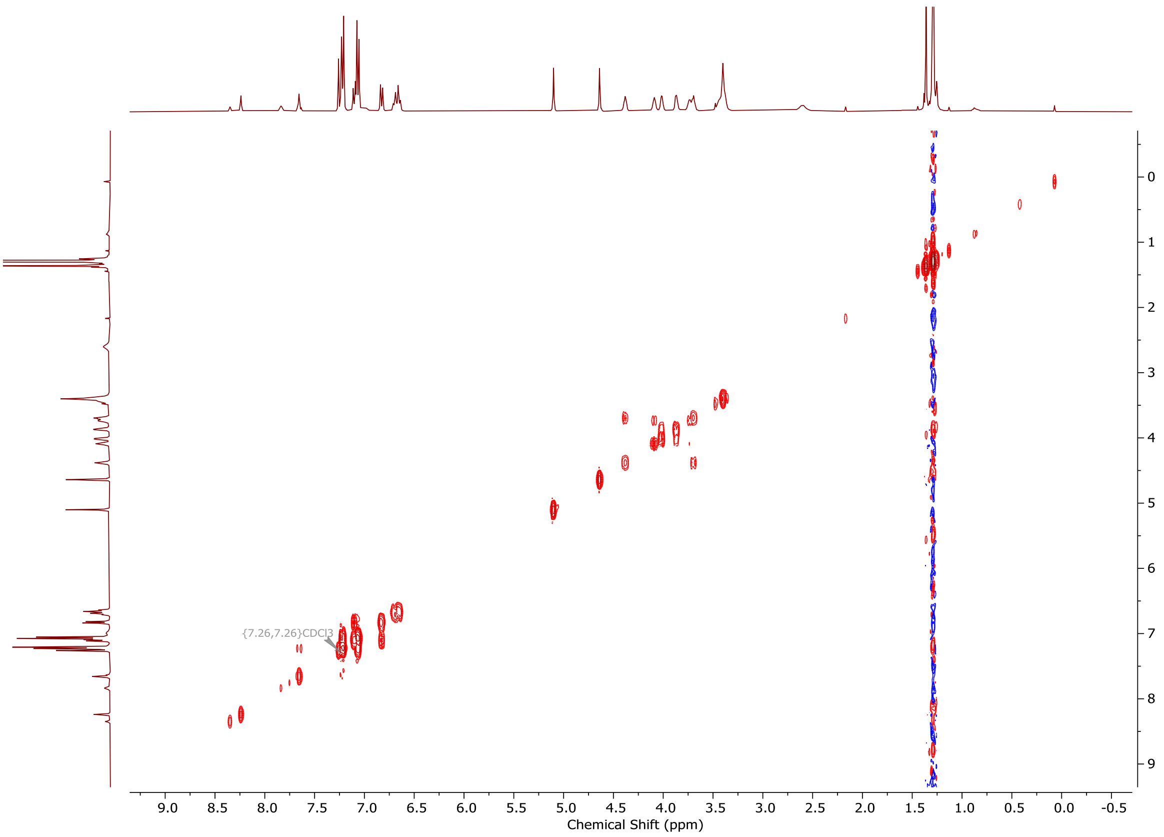

*Figure S18. ^{1}H-^{1}H COSY NMR spectrum of [2]rotaxane **8** (500 MHz, $CDCl_3$, 298 K)*

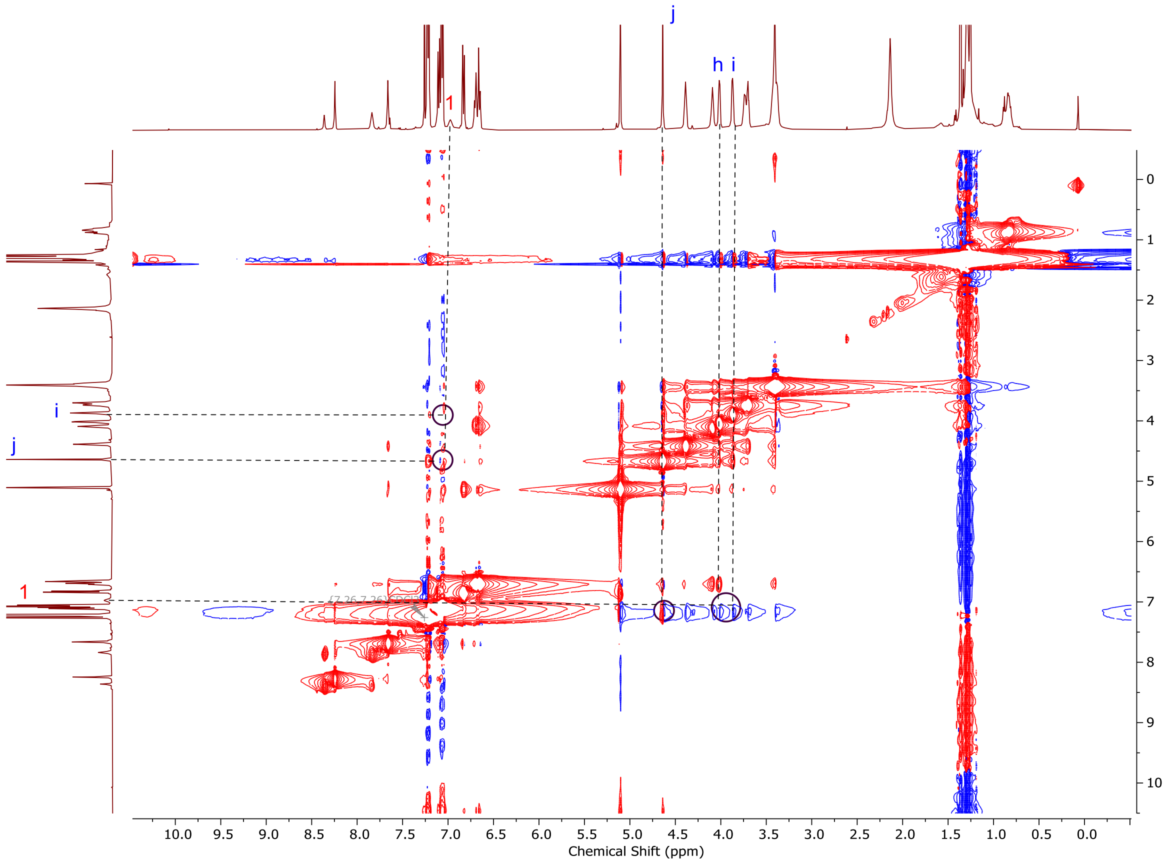

*Figure S19. ^{1}H-^{1}H ROESY NMR spectrum of [2]rotaxane **8** (500 MHz, $CDCl_3$, 298 K)*

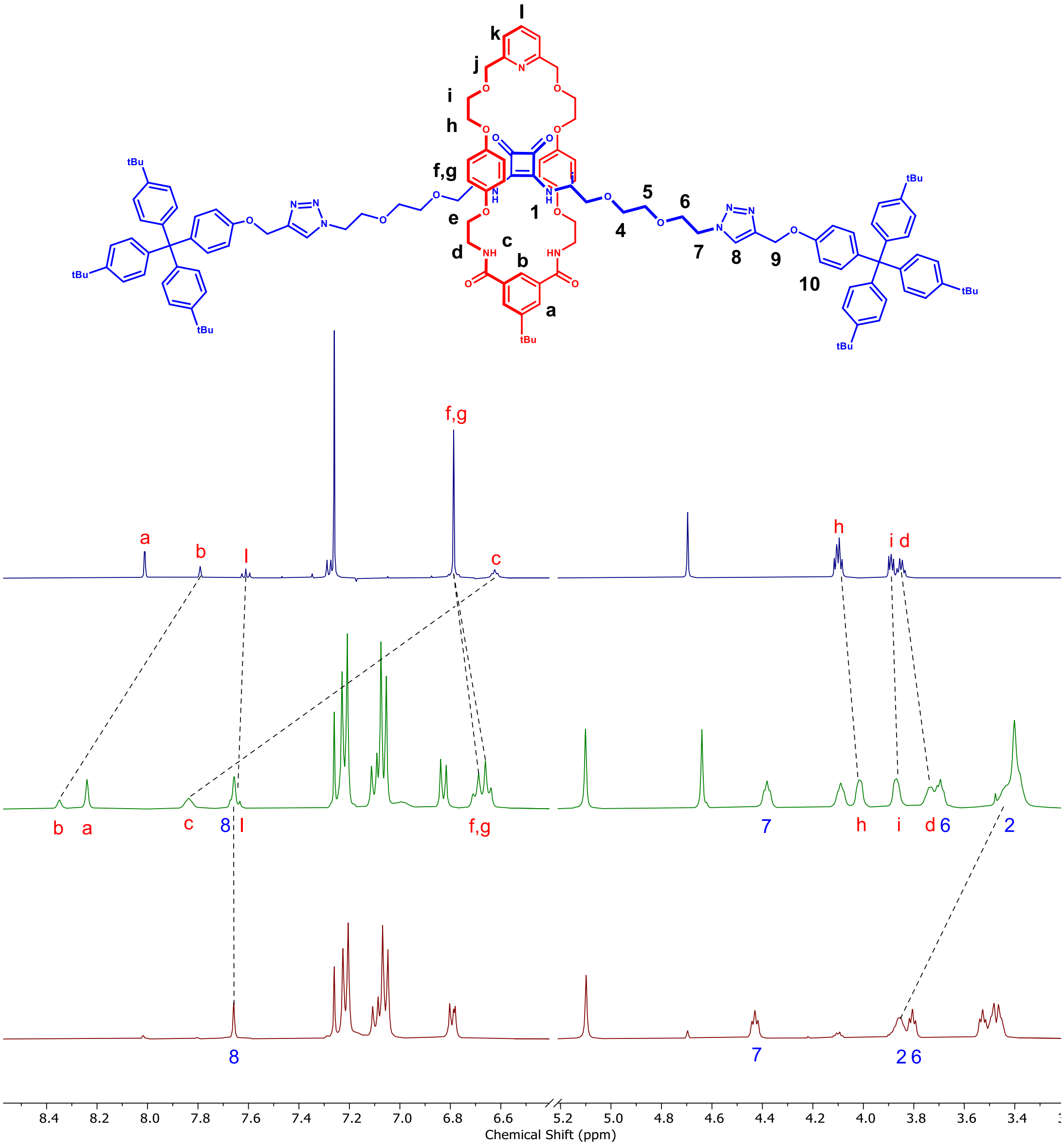

*Figure S20. Stacked ^{1}H NMR spectra of Macrocycle **C** (top) and [2]rotaxane **8** (middle) and axle **8a** (bottom) (500 MHz, $CDCl_3$, 298 K).*

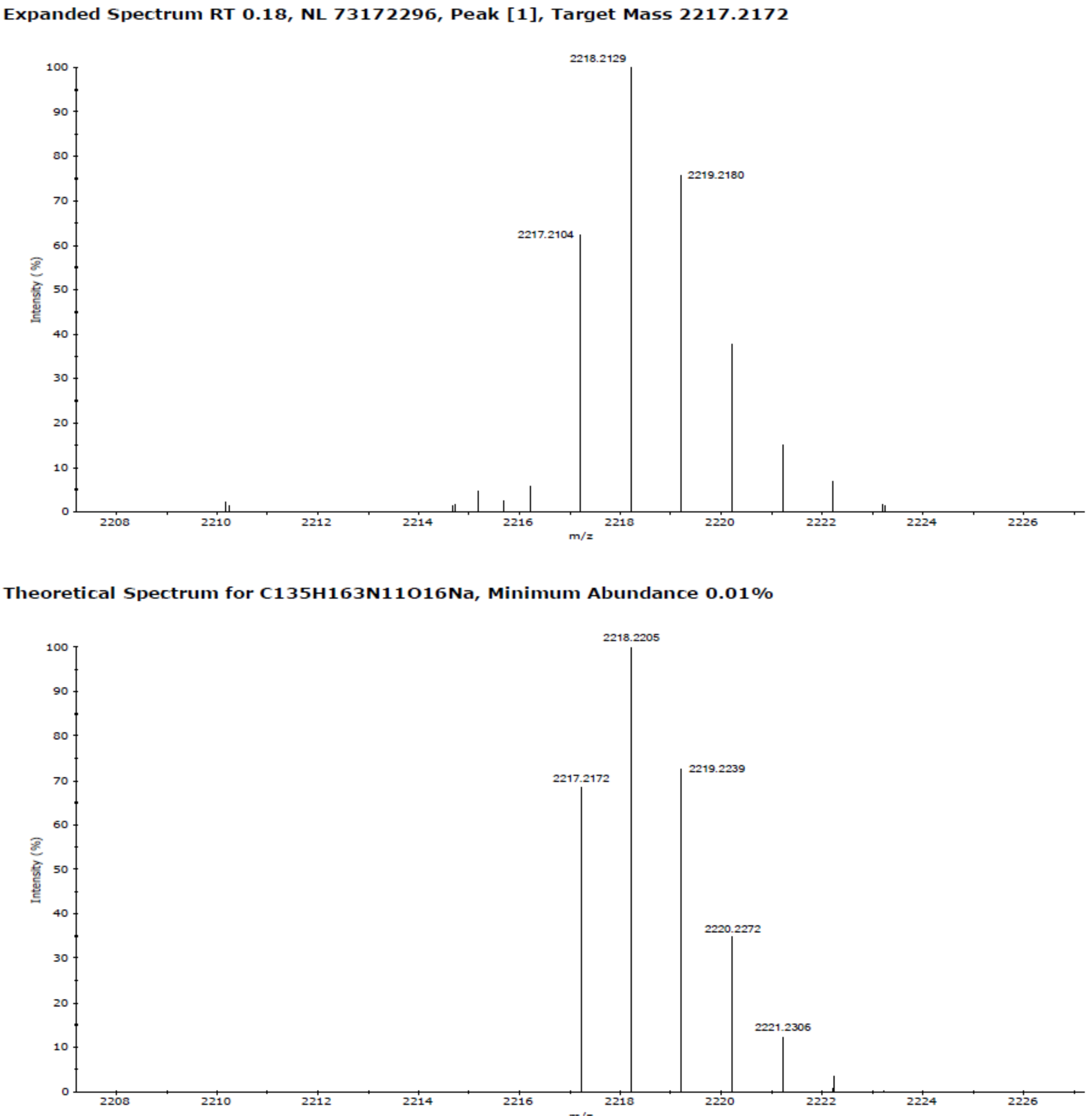

*Figure S21. High-resolution mass spectrum (ESI +ve) of **[2]rotaxane 8** (top: expanded experimental; bottom: theoretical).*

^{1}H NMR pseudorotaxane studies

Pseudo-[2]rotaxane studies were undertaken to investigate which interpenetrative assemblies were of sufficient stability for subsequent [2]rotaxane preparation. In a typical ^{1}H NMR titration procedure, aliquots of **2** were sequentially added to 2mM $CDCl_3$ solutions of macrocycles **B** and **C**. In the case of macrocycle **A**, qualitative pseudo-[2]rotaxane studies were conducted wherein three separate $CDCl_3$ solutions were prepared, containing: the macrocycle, the bis(azide), the macrocycle with an equimolar amount of **2**. All species were present at a concentration of 5.0 mM. Conversely, in the presence of a sodium template, in a typical 1H NMR titration procedure, aliquots of **2** were sequentially added to 2mM $CDCl_3$ solutions of macrocycles **A**, **B** and **C** pre-complexed with an equivalent of $NaBAr^{F}_{4}$.The ^{1}H NMR spectra of the solutions were recorded on a Bruker AVIII 500 MHz spectrometer at 298 K.

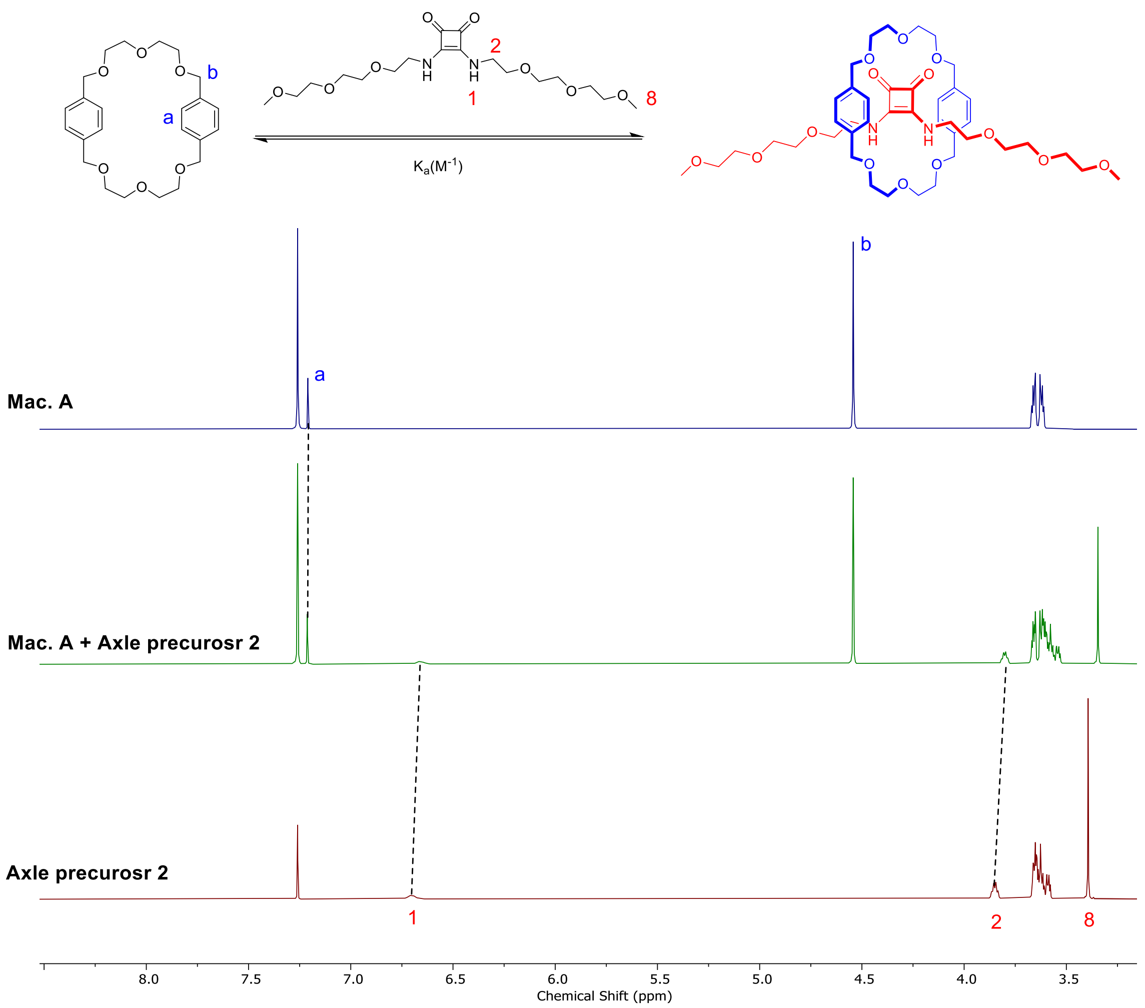

*Figure S22. Stacked ^{1}H NMR spectra of Macrocycle **A** (top), equivalent amounts of Macrocycle **A** and Axle precursor **2** (middle) and Axle precursor **2** (bottom) (500 MHz. acetone-d_6, 298 K).*

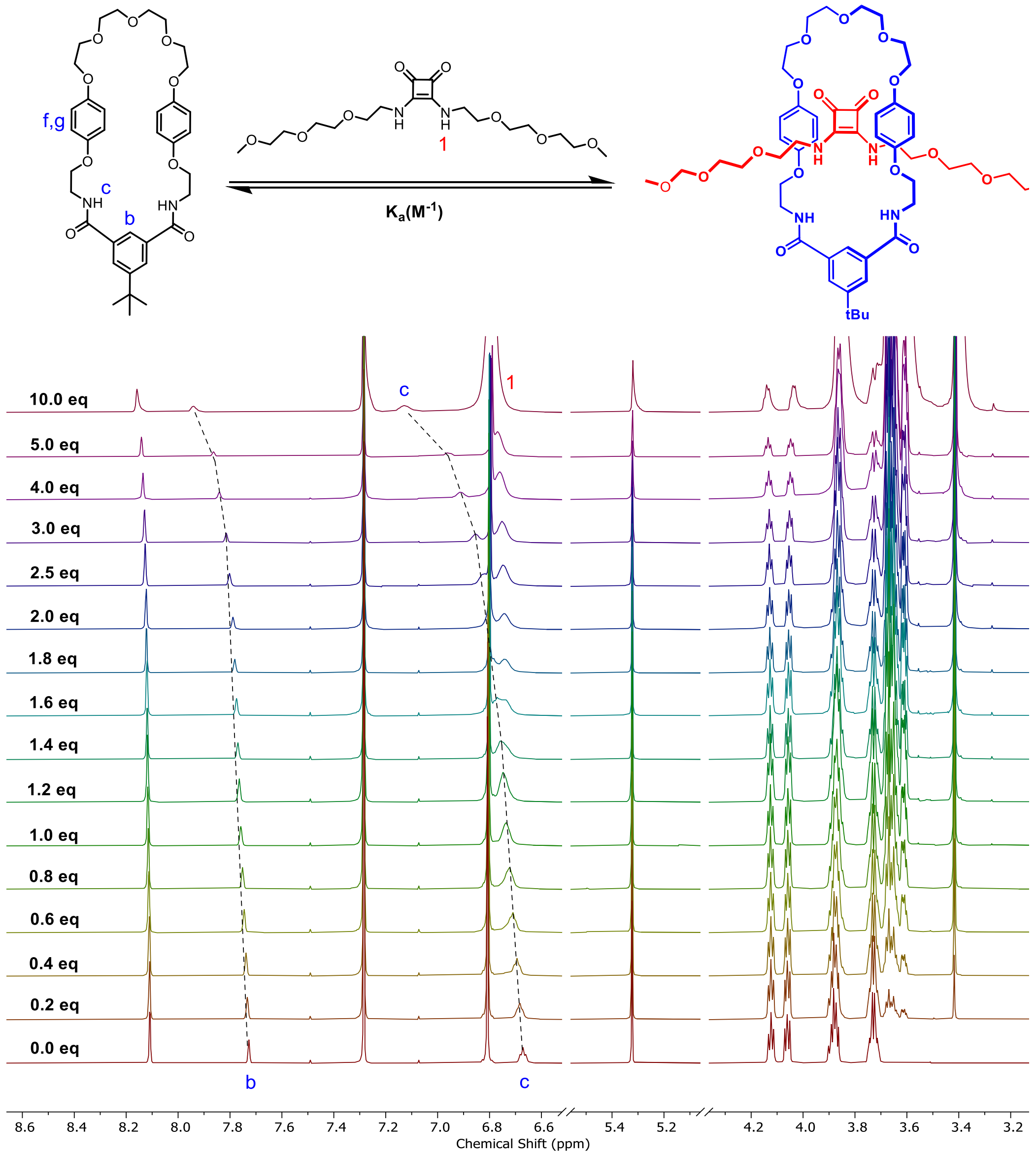

*Figure S23. Truncated ^{1}H NMR titration spectra of Macrocycle **B** upon progressive addition of 10 equivalents axle precursor **2** (500 MHz, 298 K, 7:3 $CDCl_3/CD_3CN$, [Receptor] = 2.0 mM).*

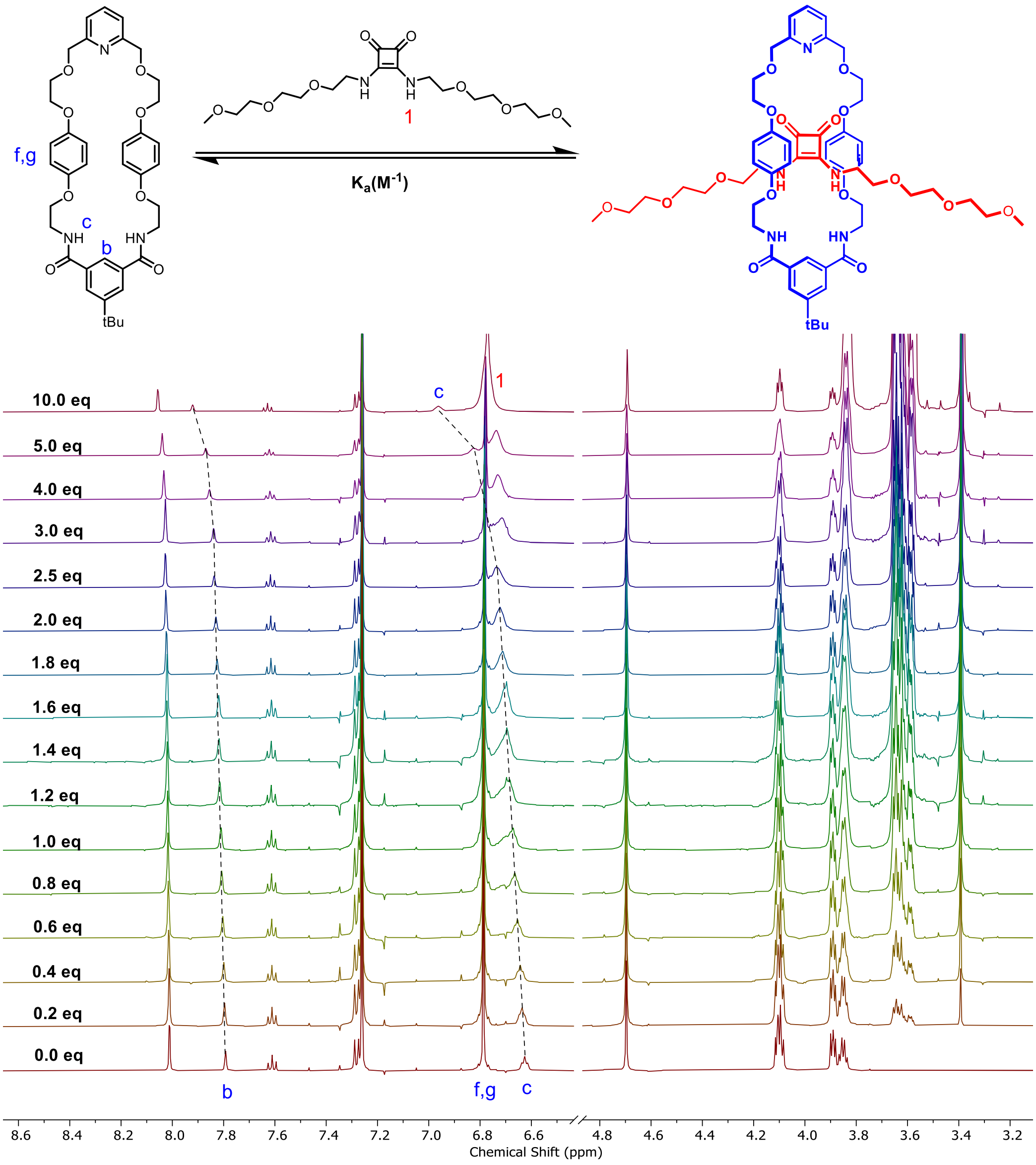

*Figure S24. Truncated ^{1}H NMR titration spectra of Macrocycle **C** upon progressive addition of 10 equivalents axle precursor **2** (500 MHz, 298 K, 7:3 $CDCl_3$/CD_3CN, [Receptor] = 2.0 mM).*

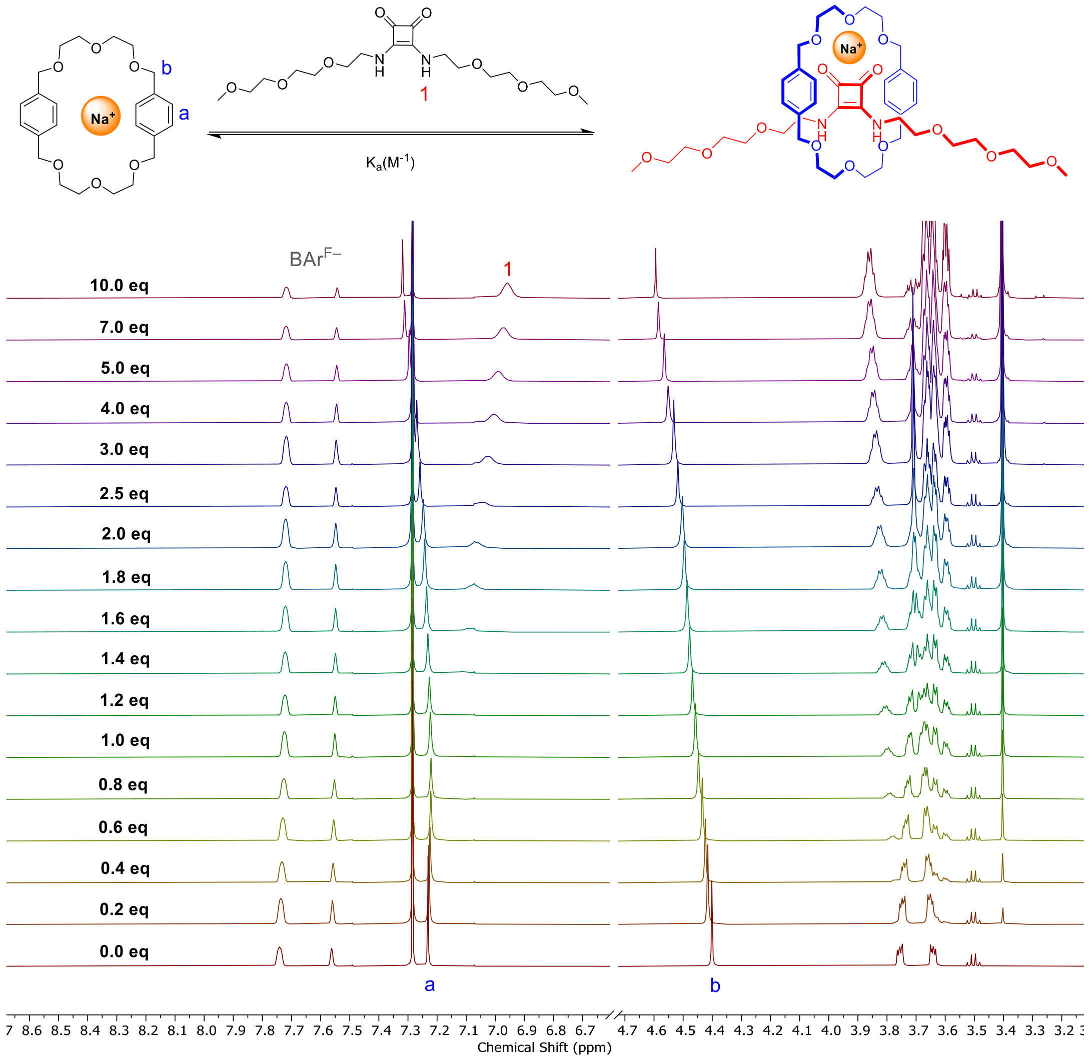

*Figure S25. Truncated ^{1}H NMR titration spectra of Macrocycle **A** + 1 eq. NaBAr$^{F}_{4}$ upon progressive addition of 10 equivalents axle (500 MHz, 298 K, 7:3 $CDCl_3$/CD_3CN, [Receptor] = 2.0 mM).*

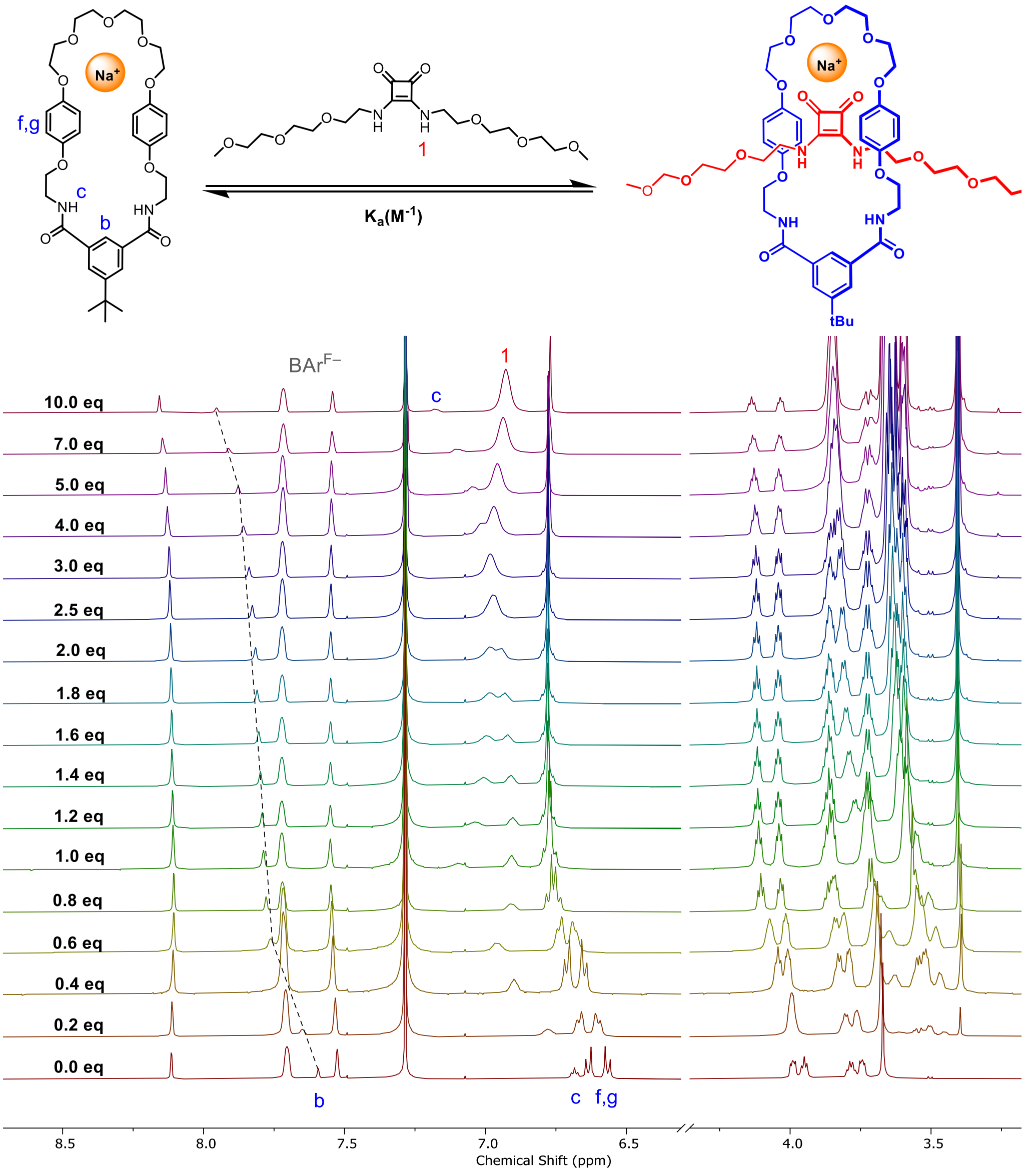

*Figure S26. Truncated ^{1}H NMR titration spectra of Macrocycle **B** + 1 eq. $NaBAr^{F}_{4}$ upon progressive addition of 10 equivalents axle (500 MHz, 298 K, 7:3 $CDCl_3/CD_3CN$, [Receptor] = 2.0 mM).*

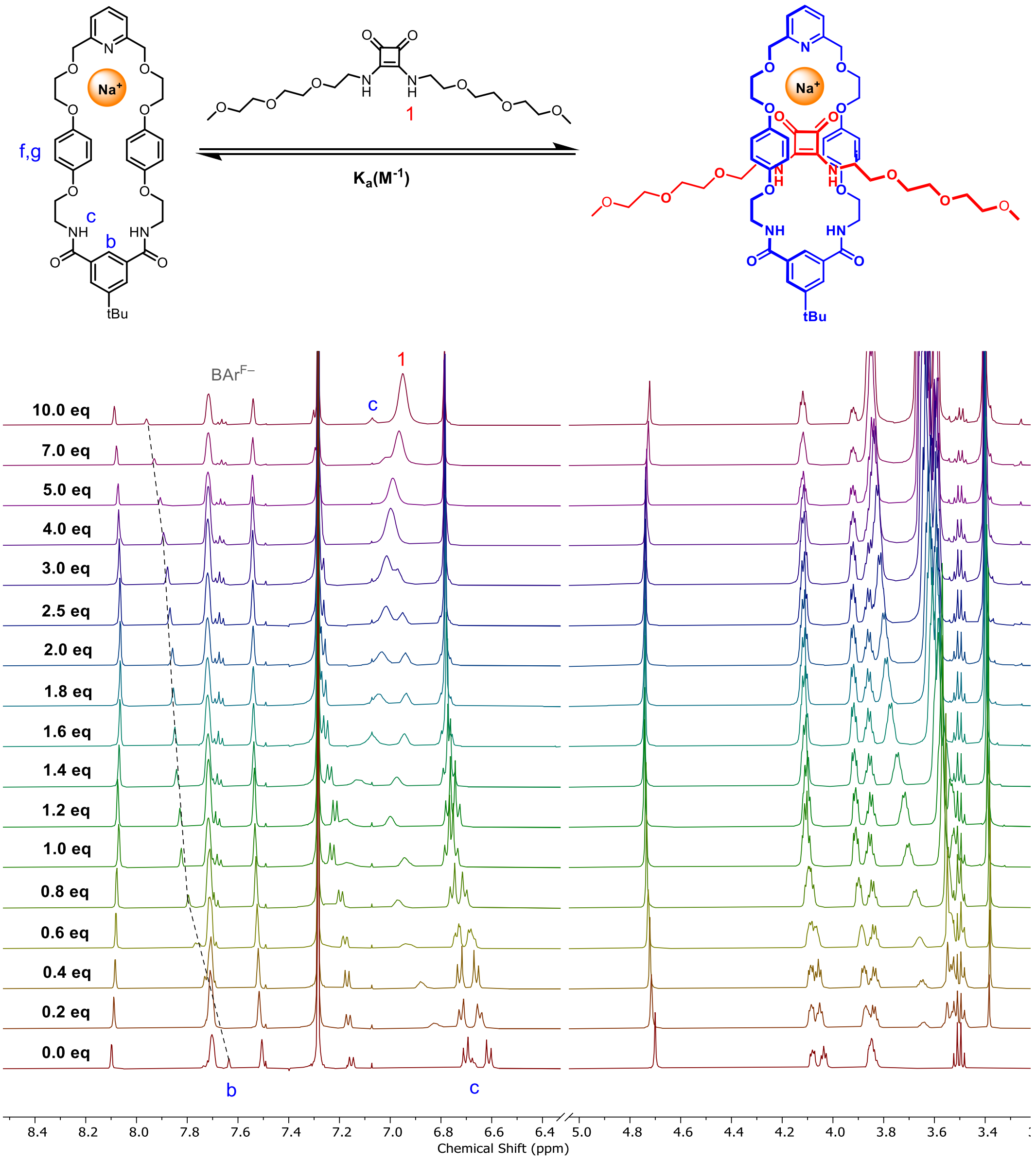

Figure S27. Truncated ^{1}H NMR titration spectra of Macrocycle ***C*** *+ 1 eq. NaBAr$^{F}_{4}$ upon progressive addition of 10 equivalents axle (500 MHz, 298 K, 7:3 $CDCl_3$/CD_3CN, [Receptor] = 2.0 mM).*

^{1}H NMR binding studies

General procedure

All ^{1}H NMR titration experiments were performed on a Bruker AVIII 500 MHz spectrometer at 298 K. In a typical cation or anion titration, a 1.0 mM solution of the neutral receptor was prepared in 7:3 $CDCl_3/CD_3CN$. In an ion-pair titration, an equimolar amount of the [2]rotaxane and $NaBAr^F_4$, each present at 1.0 mM concentration, was dissolved in 7:3 $CDCl_3/CD_3CN$. The solution was sonicated for 20 min to form the metal-rotaxane complex. A 50 mM solution of $NaBAr^F_4$ or TBAX (X = Cl, Br, I) was added in aliquots to the solution containing the receptor, where 1.0 equivalent of the salt added corresponds to 10.0 µL of the salt solution. 17 spectra were recorded, corresponding to 0.0, 0.2, 0.4, 0.6, 0.8, 1.0, 1.2, 1.4, 1.6, 1.8, 2.0, 2.5, 3.0, 4.0, 5.0, 7.0, 10.0 equivalents of the added guest ion. The binding of cations and anions to all receptors were found to be fast on the NMR timescale. For cation titrations, the chemical shifts of multiple peaks around the cation binding site (H_c, H_d, H_h, H_i, H_i) were monitored and used for subsequent fitting. For anion and ion-pair titrations, the chemical shift of the internal benzene proton H_b, amide protons H_c and squaramide protons H_1 were used. The values of the observed chemical shift(s) and concentration of guest at each titration point were entered into the Bindfit software alongside initial estimates of the binding constants and limiting chemical shifts. These parameters were refined using nonlinear least-squares analyses to obtain the best fit between empirical and calculated chemical shifts based on the 1:1 host-guest binding model. The input parameters were varied until convergence of the best fit values of the binding constants was attained.

Anion titrations: *^{1}H NMR titration spectra*

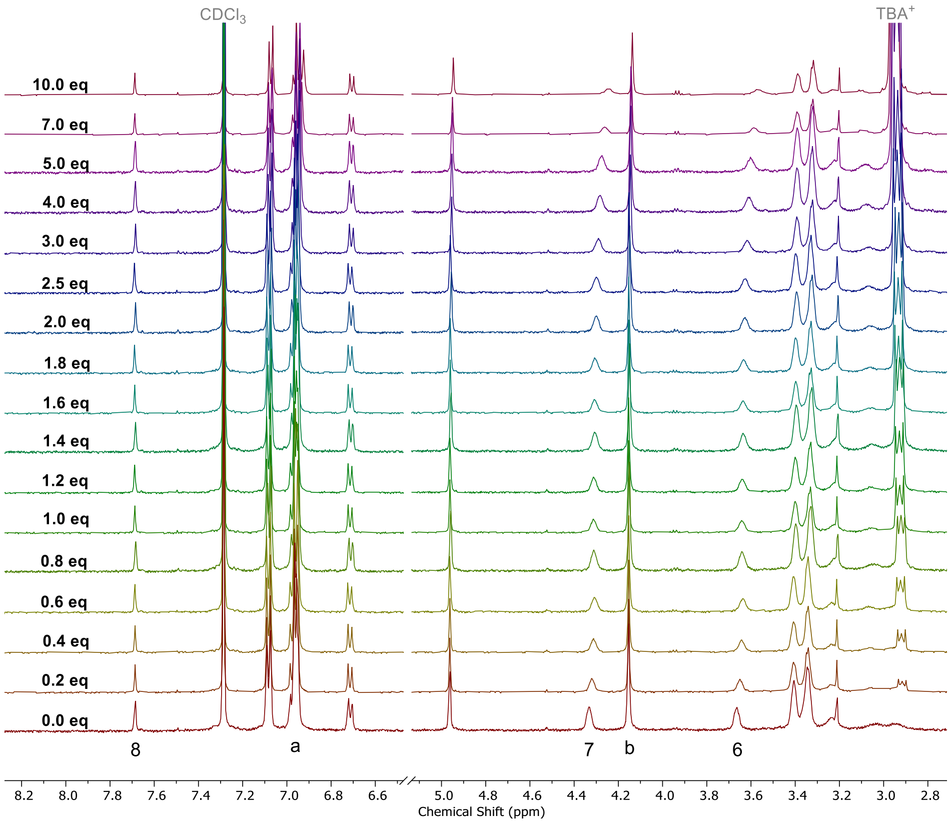

*Figure S28. Truncated ^{1}H NMR titration spectra of [2]rotaxane **6** upon progressive addition of 10 equivalents TBACl (500 MHz, 298 K, 7:3 $CDCl_3/CD_3CN$, [Receptor] = 1.0 mM).*

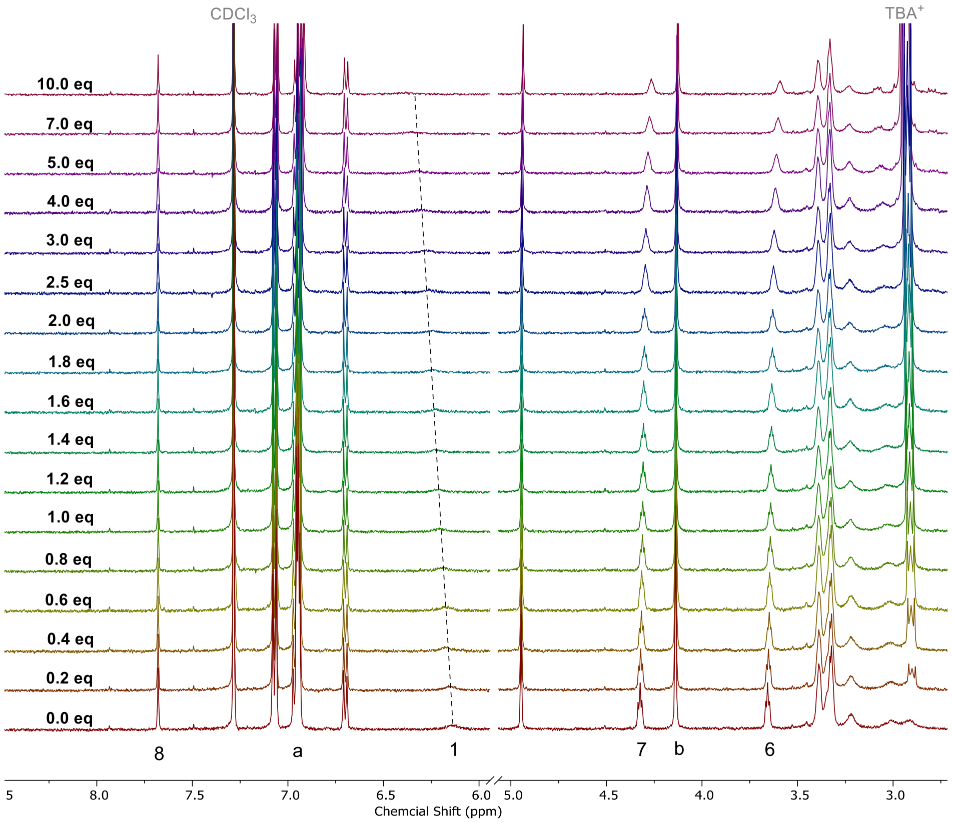

*Figure S29. Truncated ^{1}H NMR titration spectra of [2]rotaxane **6** upon progressive addition of 10 equivalents TBABr (500 MHz, 298 K, 7:3 $CDCl_3/CD_3CN$, [Receptor] = 1.0 mM).*

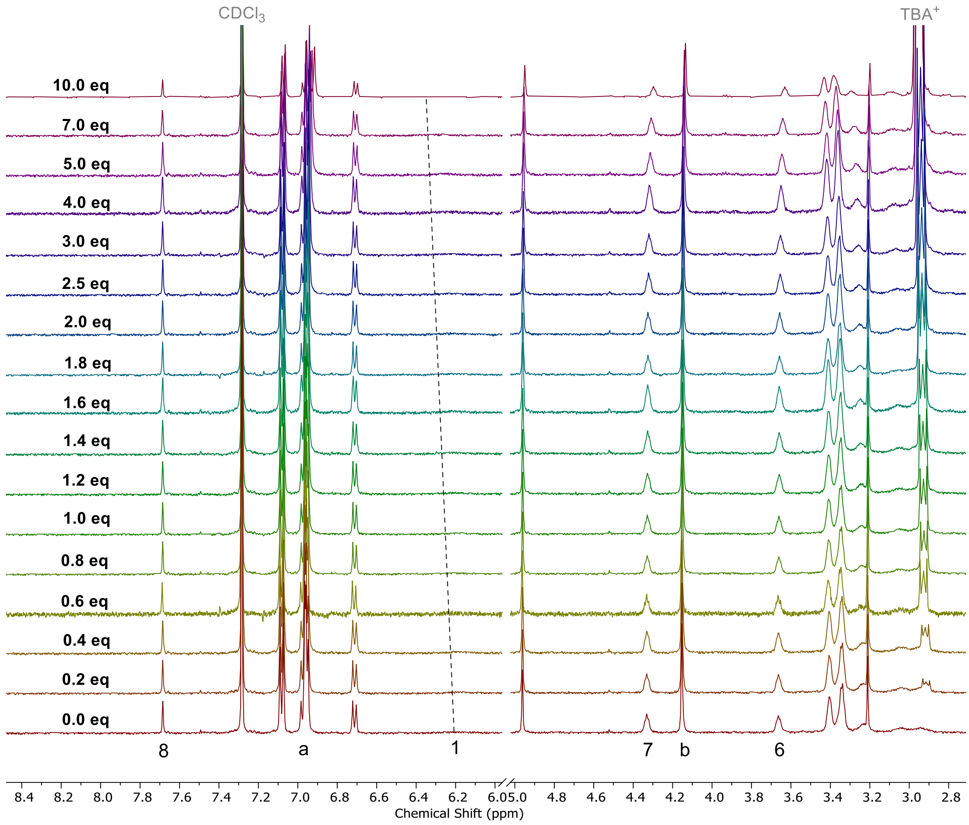

*Figure S30. Truncated ^{1}H NMR titration spectra of [2]rotaxane **6** upon progressive addition of 10 equivalents TBAI (500 MHz, 298 K, 7:3 $CDCl_3/CD_3CN$, [Receptor] = 1.0 mM).*

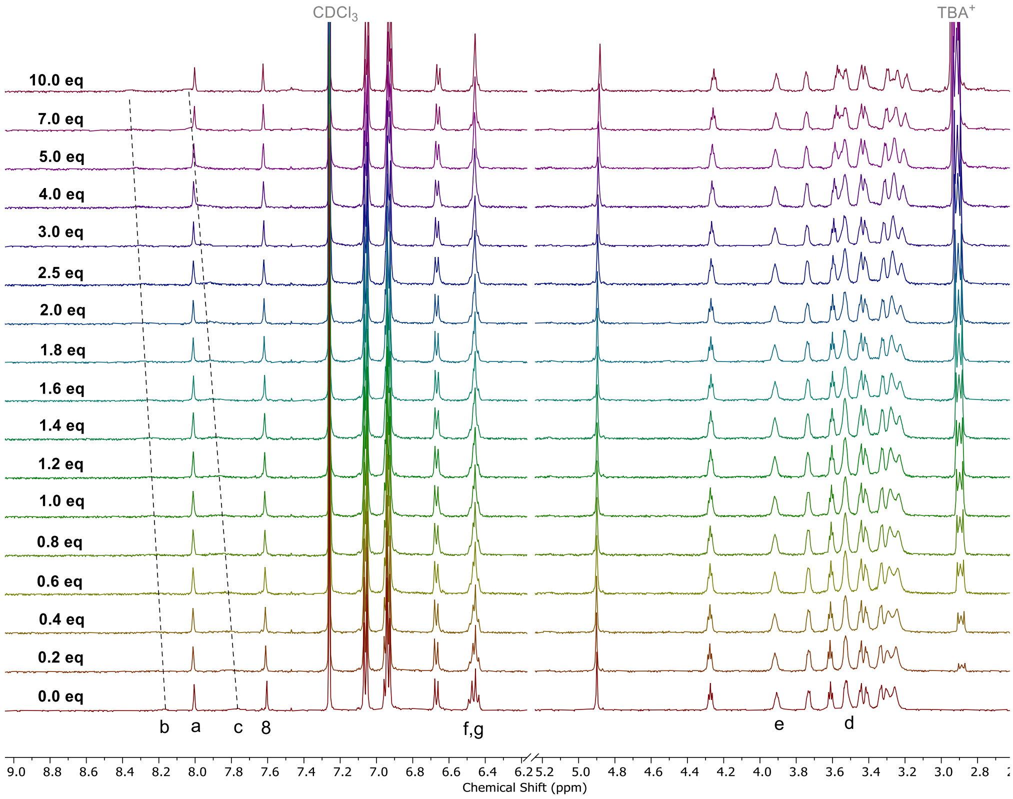

*Figure S31. Truncated ^{1}H NMR titration spectra of [2]rotaxane **7** upon progressive addition of 10 equivalents TBACl (500 MHz, 298 K, 7:3 $CDCl_3/CD_3CN$, [Receptor] = 1.0 mM).*

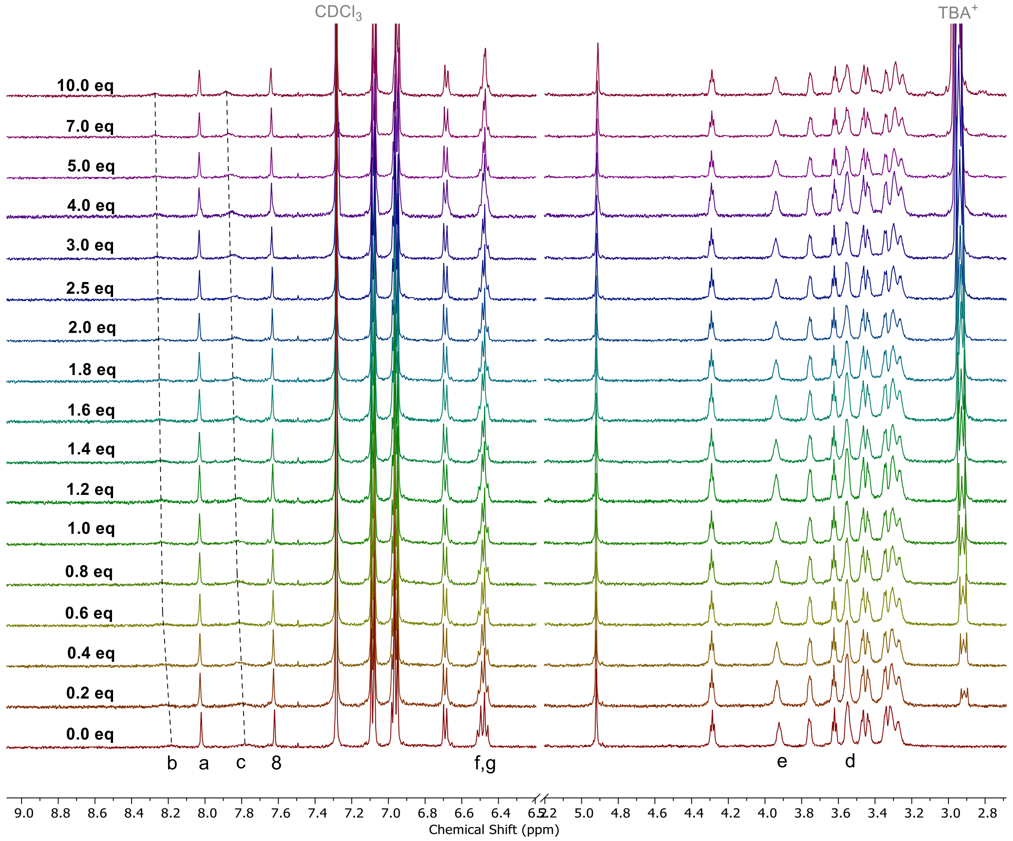

*Figure S32. Truncated ^{1}H NMR titration spectra of [2]rotaxane **7** upon progressive addition of 10 equivalents TBABr (500 MHz, 298 K, 7:3 $CDCl_3/CD_3CN$, [Receptor] = 1.0 mM).*

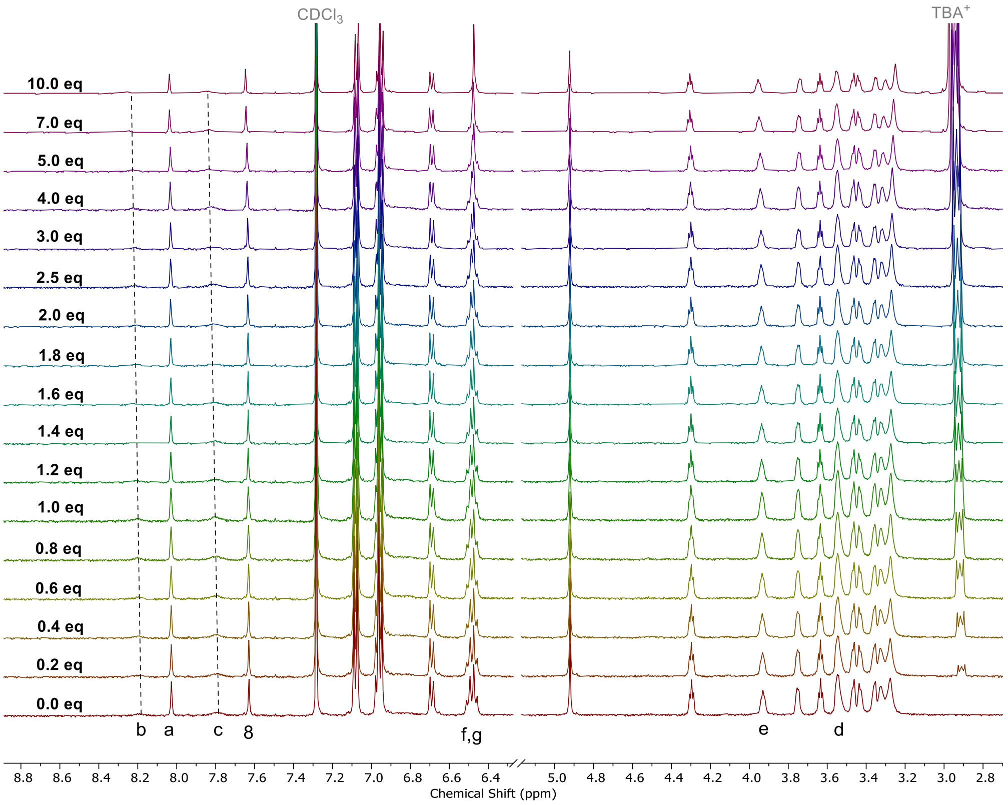

*Figure S33. Truncated ^{1}H NMR titration spectra of [2]rotaxane **7** upon progressive addition of 10 equivalents TBAI (500 MHz, 298 K, 7:3 $CDCl_3/CD_3CN$, [Receptor] = 1.0 mM).*

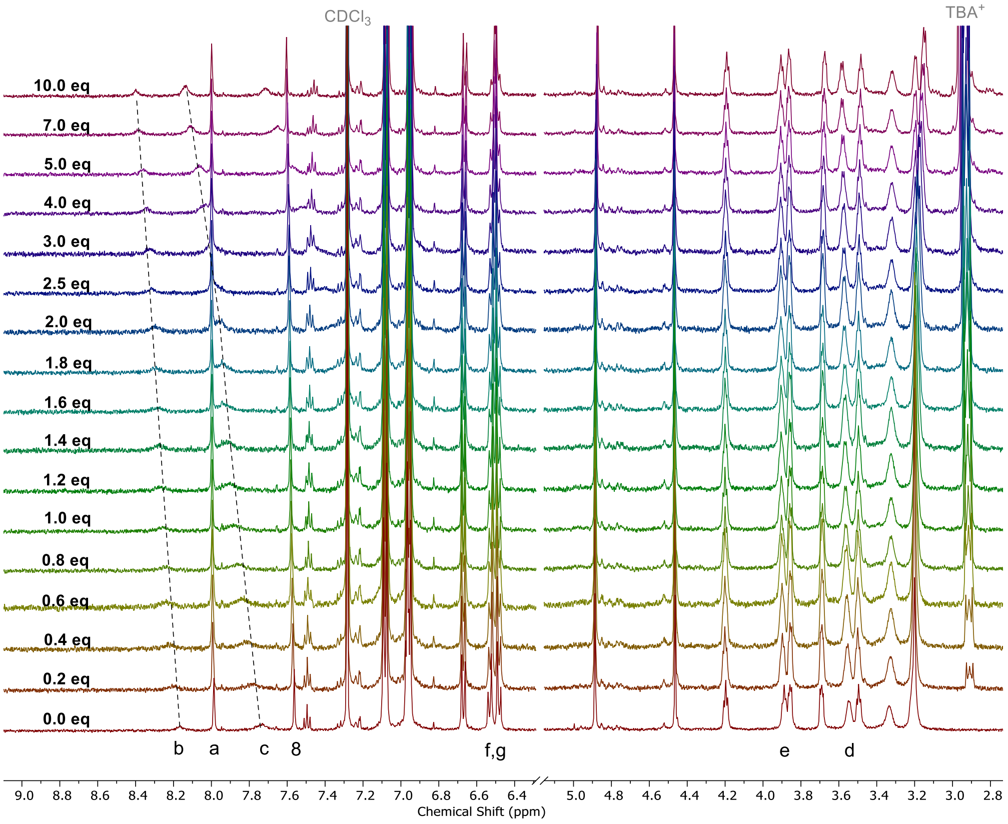

*Figure S34. Truncated ^{1}H NMR titration spectra of [2]rotaxane **8** upon progressive addition of 10 equivalents TBACl (500 MHz, 298 K, 7:3 $CDCl_3/CD_3CN$, [Receptor] = 1.0 mM).*

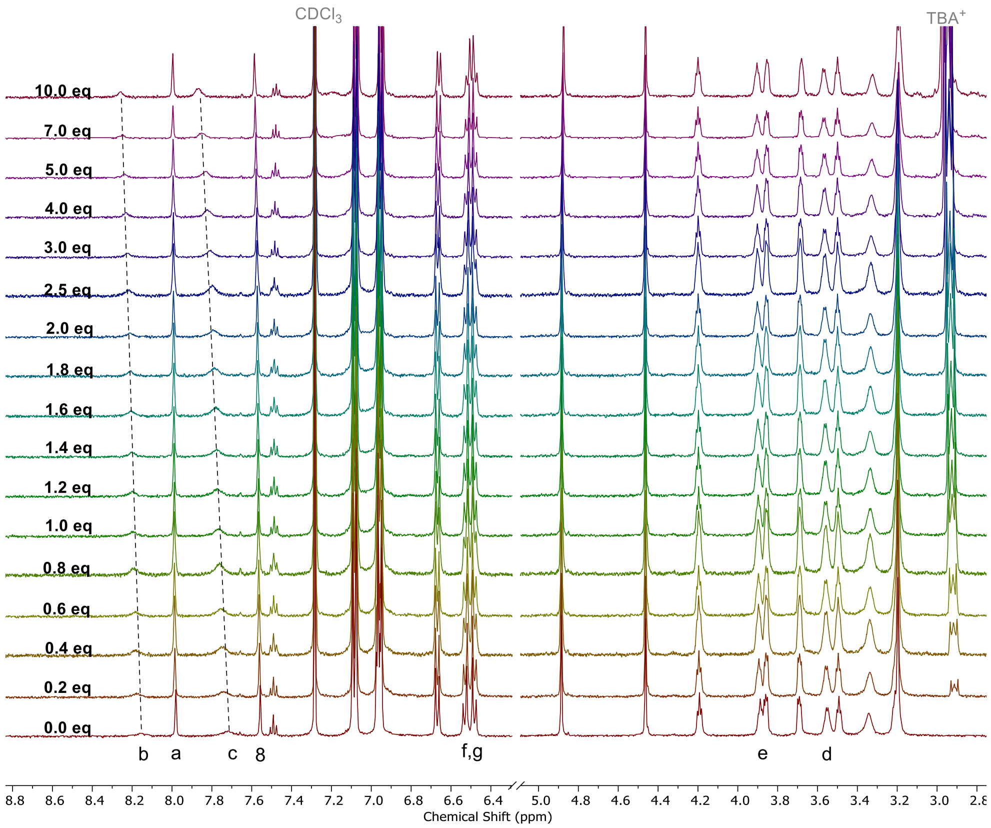

*Figure S35. Truncated ^{1}H NMR titration spectra of [2]rotaxane **8** upon progressive addition of 10 equivalents TBABr (500 MHz, 298 K, 7:3 $CDCl_3/CD_3CN$, [Receptor] = 1.0 mM).*

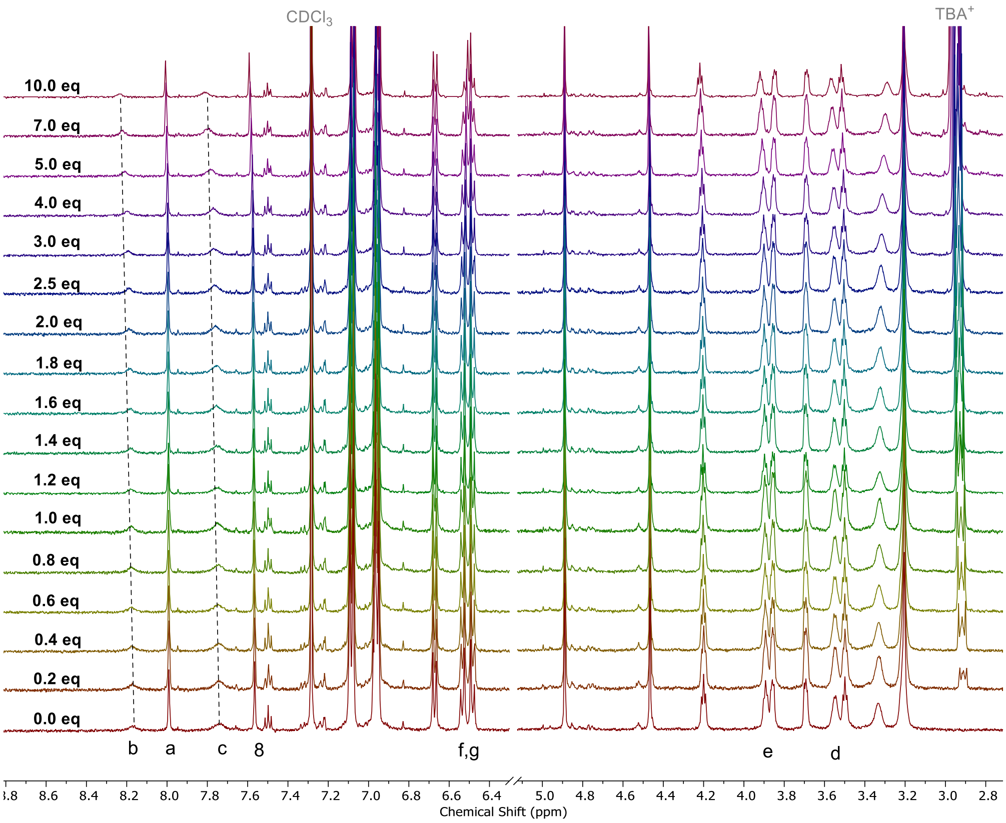

*Figure S36. Truncated ^{1}H NMR titration spectra of [2]rotaxane **8** upon progressive addition of 10 equivalents TBAI (500 MHz, 298 K, 7:3 $CDCl_3/CD_3CN$, [Receptor] = 1.0 mM).*

Cation titrations: ^{1}*H NMR titration spectra*

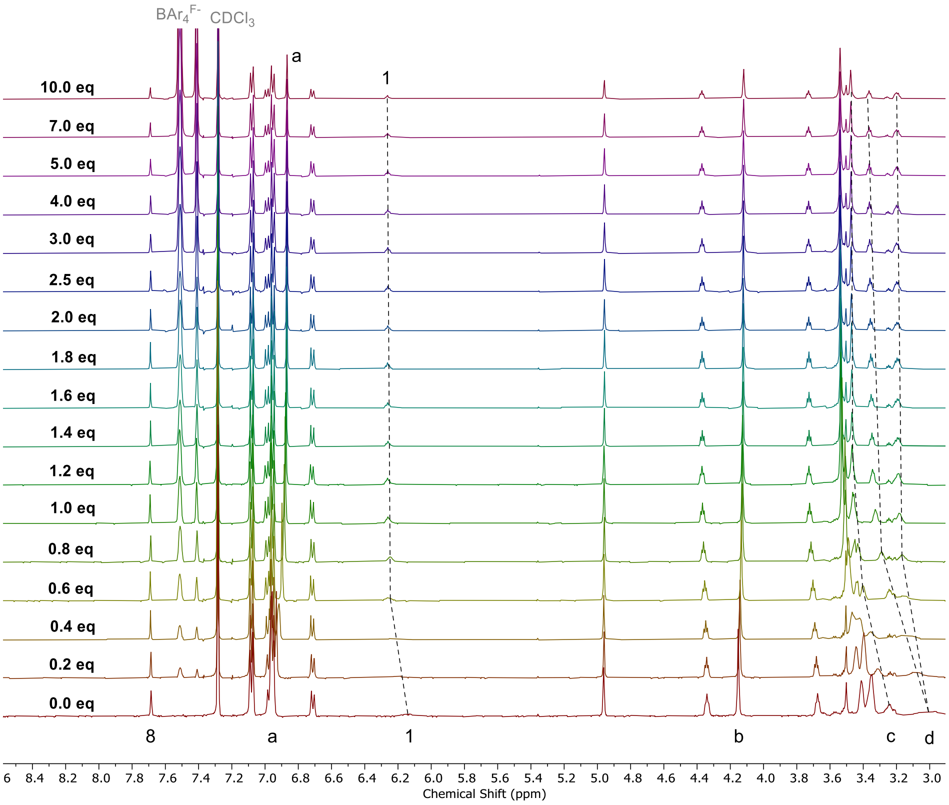

*Figure S37. Truncated ^{1}H NMR titration spectra of [2]rotaxane **6** upon progressive addition of 10 equivalents $NaBAr^{F}_{4}$ (500 MHz, 298 K, 7:3 $CDCl_3/CD_3CN$, [Receptor] = 1.0 mM).*

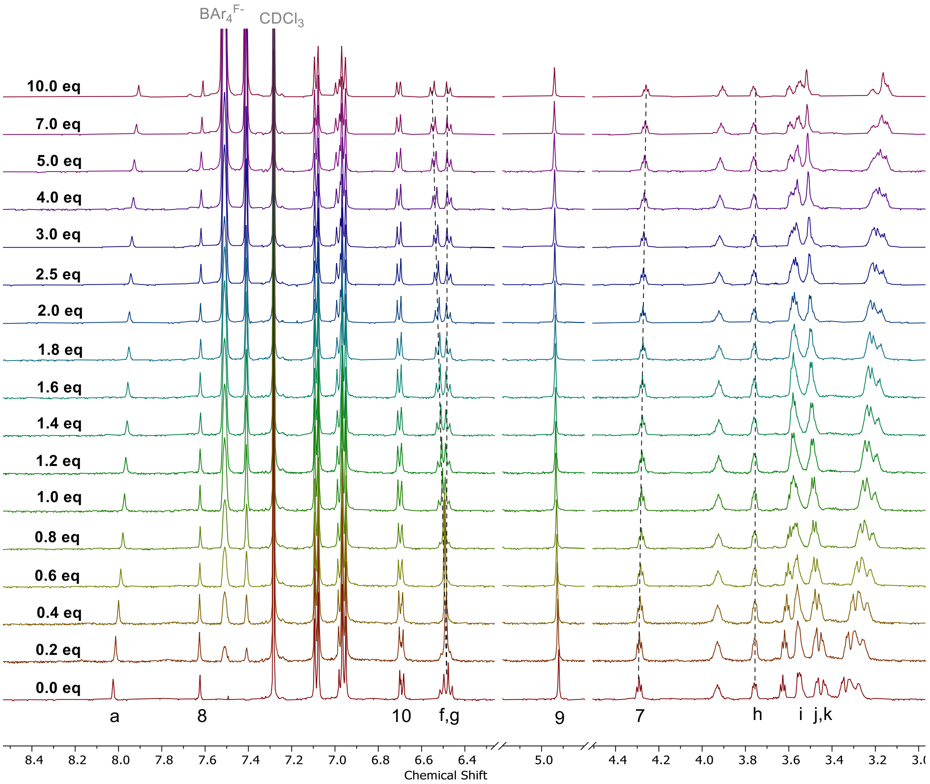

*Figure S38. Truncated ^{1}H NMR titration spectra of [2]rotaxane **7** upon progressive addition of 10 equivalents $NaBAr^{F}_{4}$ (500 MHz, 298 K, 7:3 $CDCl_3/CD_3CN$, [Receptor] = 1.0 mM).*

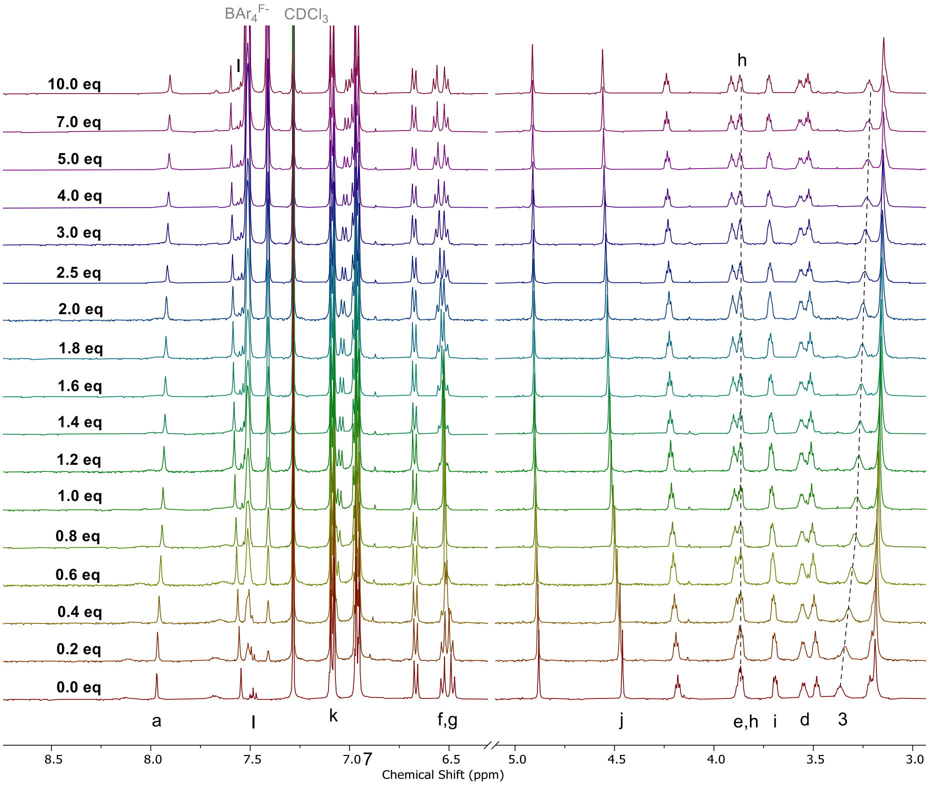

*Figure S39. Truncated ^{1}H NMR titration spectra of [2]rotaxane **8** upon progressive addition of 10 equivalents $NaBAr^{F}_{4}$ (500 MHz, 298 K, 7:3 $CDCl_3/CD_3CN$, [Receptor] = 1.0 mM).*

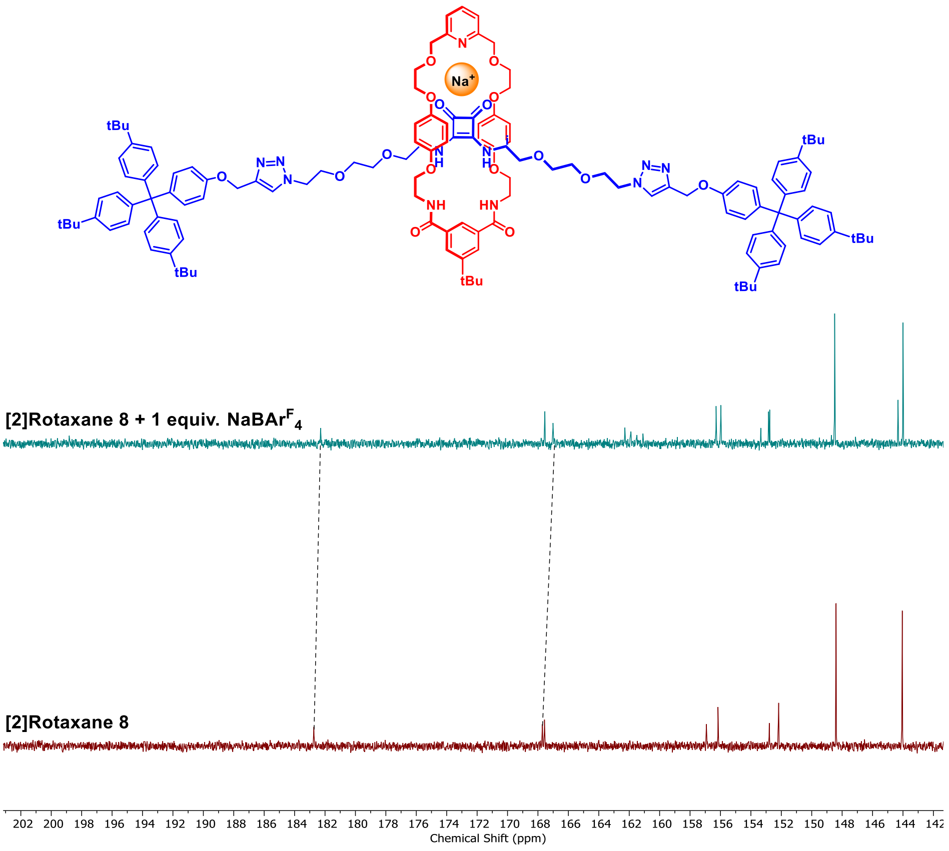

*Figure S40. Truncated ^{13}C NMR titration spectra of [2]rotaxane **8** upon addition of 1 equivalent $NaBAr^{F}_{4}$ (500 MHz, 298 K, $CDCl_3$, [Receptor] = 16.0 mM).*

Ion-pair titrations: *¹H NMR titration spectra*

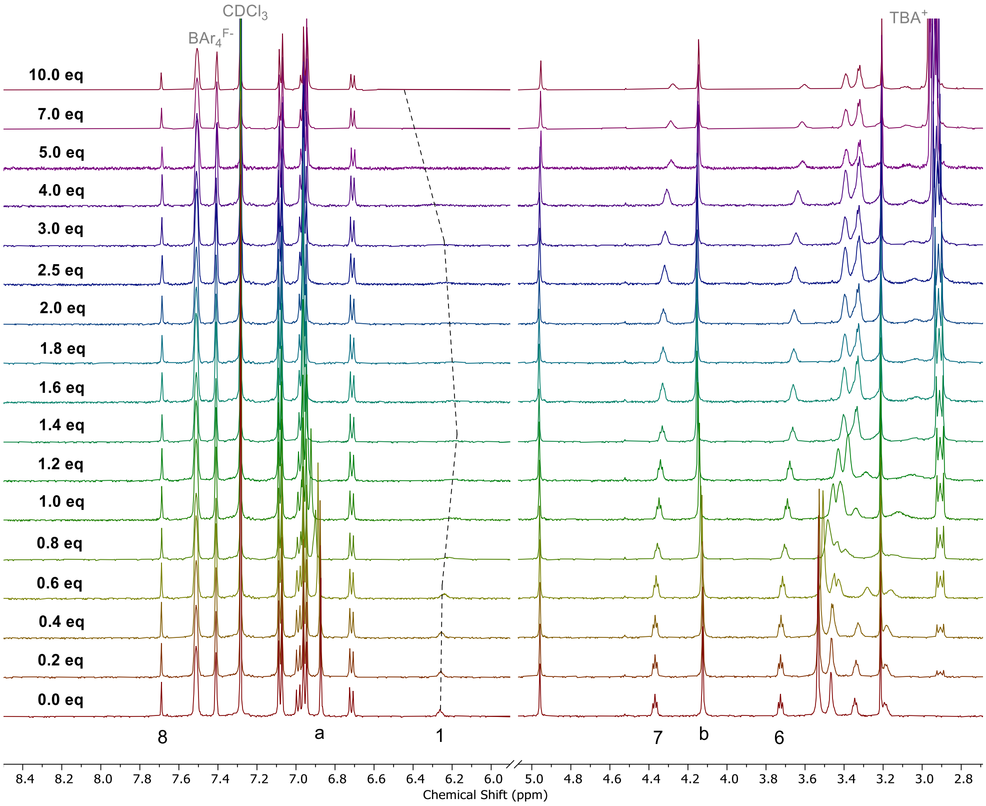

*Figure S41. Truncated ¹H NMR titration spectra of [2]rotaxane **6** upon progressive addition of 10 equivalents TBACl in the presence of 1 equivalent NaBAr$^F{}_4$ (500 MHz, 298 K, 7:3 $CDCl_3$/CD_3CN, [Receptor] = [NaBAr$_4{}^F$] = 1.0 mM).*

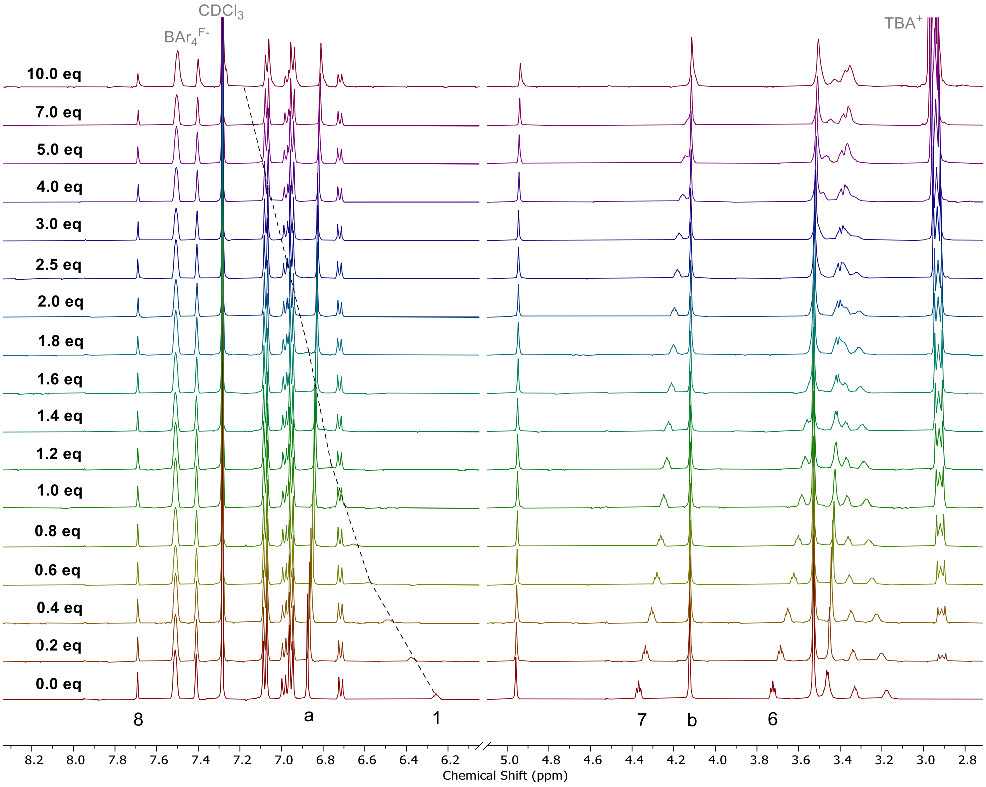

*Figure S42. Truncated ¹H NMR titration spectra of [2]rotaxane **6** upon progressive addition of 10 equivalents TBABr in the presence of 1 equivalent NaBAr$^F{}_4$ (500 MHz, 298 K, 7:3 $CDCl_3$/CD_3CN, [Receptor] = [NaBAr$_4{}^F$] = 1.0 mM).*

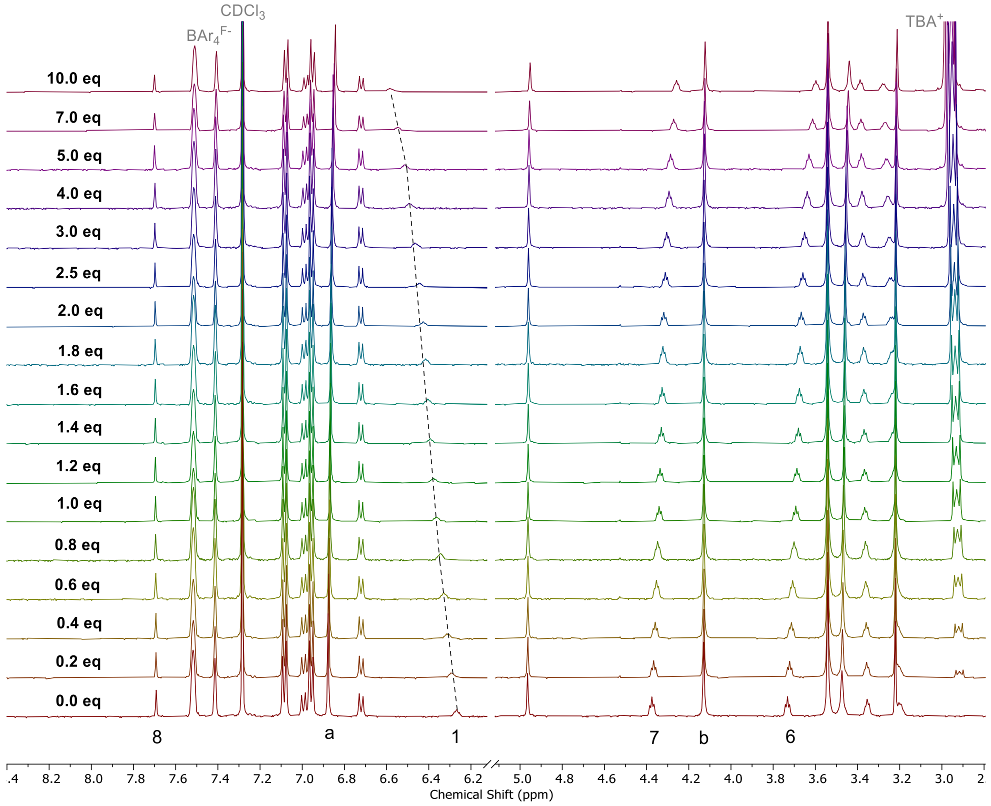

*Figure S43. Truncated ^{1}H NMR titration spectra of [2]rotaxane **6** upon progressive addition of 10 equivalents TBAI in the presence of 1 equivalent NaBAr$^{F}_{4}$ (500 MHz, 298 K, 7:3 $CDCl_3$/CD_3CN, [Receptor] = [NaBAr$_4^F$] = 1.0 mM).*

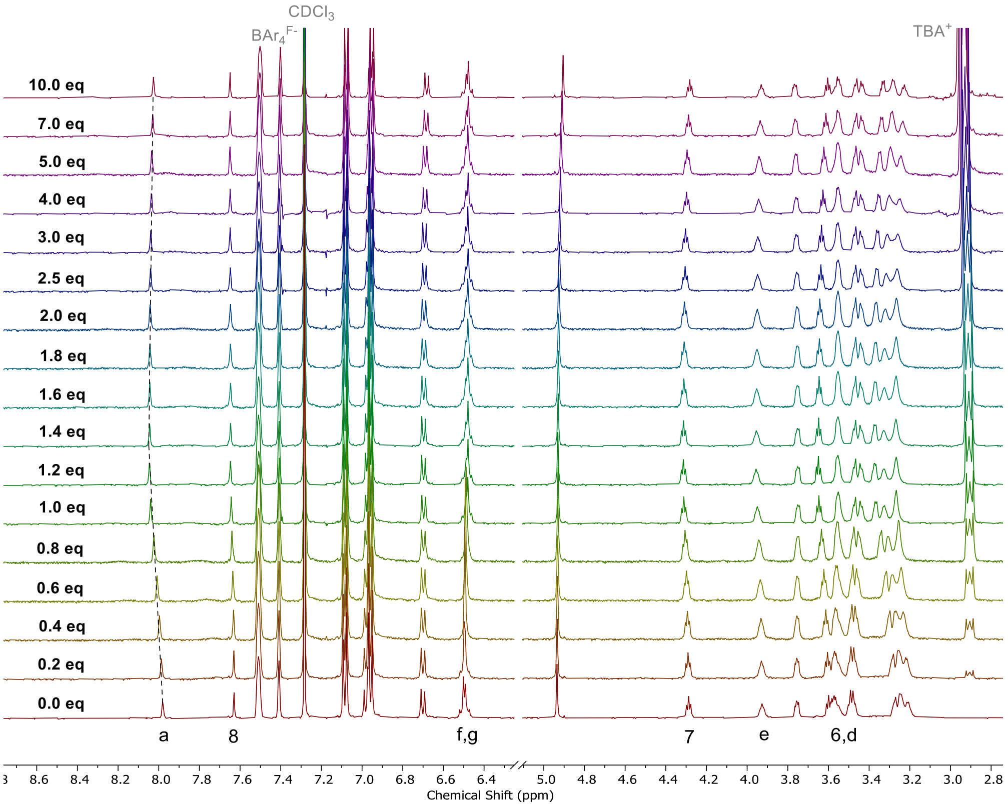

*Figure S44. Truncated ^{1}H NMR titration spectra of [2]rotaxane **7** upon progressive addition of 10 equivalents TBACl in the presence of 1 equivalent NaBAr$^{F}_{4}$ (500 MHz, 298 K, 7:3 $CDCl_3$/CD_3CN, [Receptor] = [NaBAr$_4^F$] = 1.0 mM).*

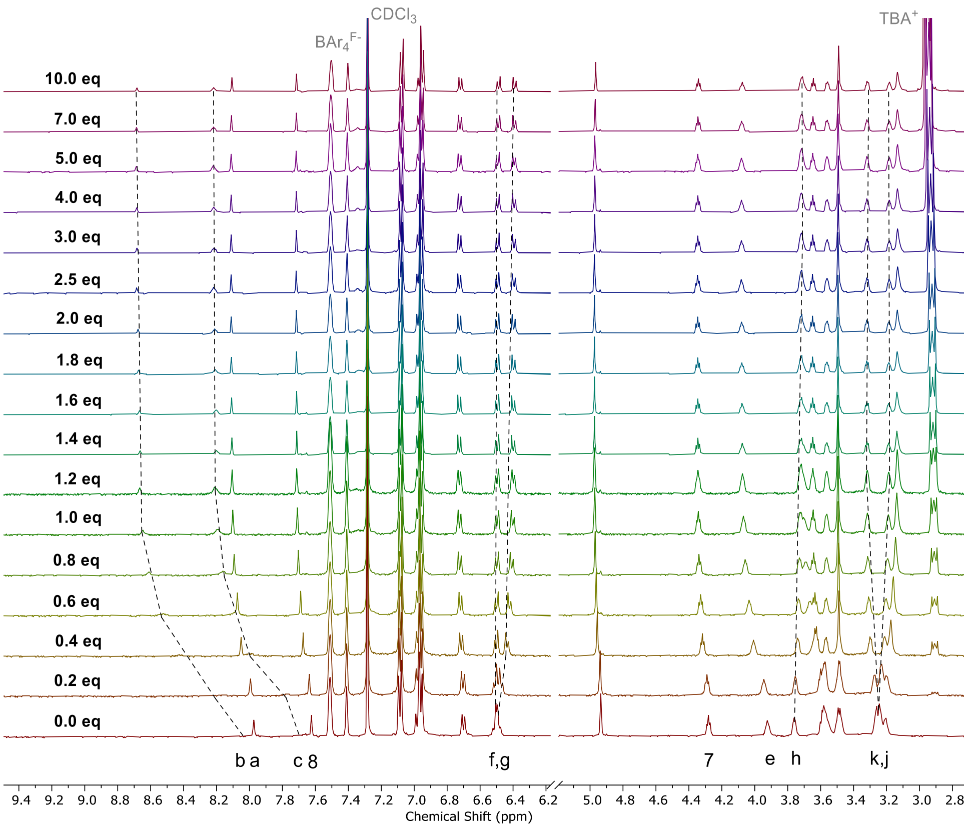

*Figure S45. Truncated ^{1}H NMR titration spectra of [2]rotaxane **7** upon progressive addition of 10 equivalents TBABr in the presence of 1 equivalent $NaBAr^{F}_{4}$ (500 MHz, 298 K, 7:3 $CDCl_3/CD_3CN$, [Receptor] = [$NaBAr_4^F$] = 1.0 mM).*

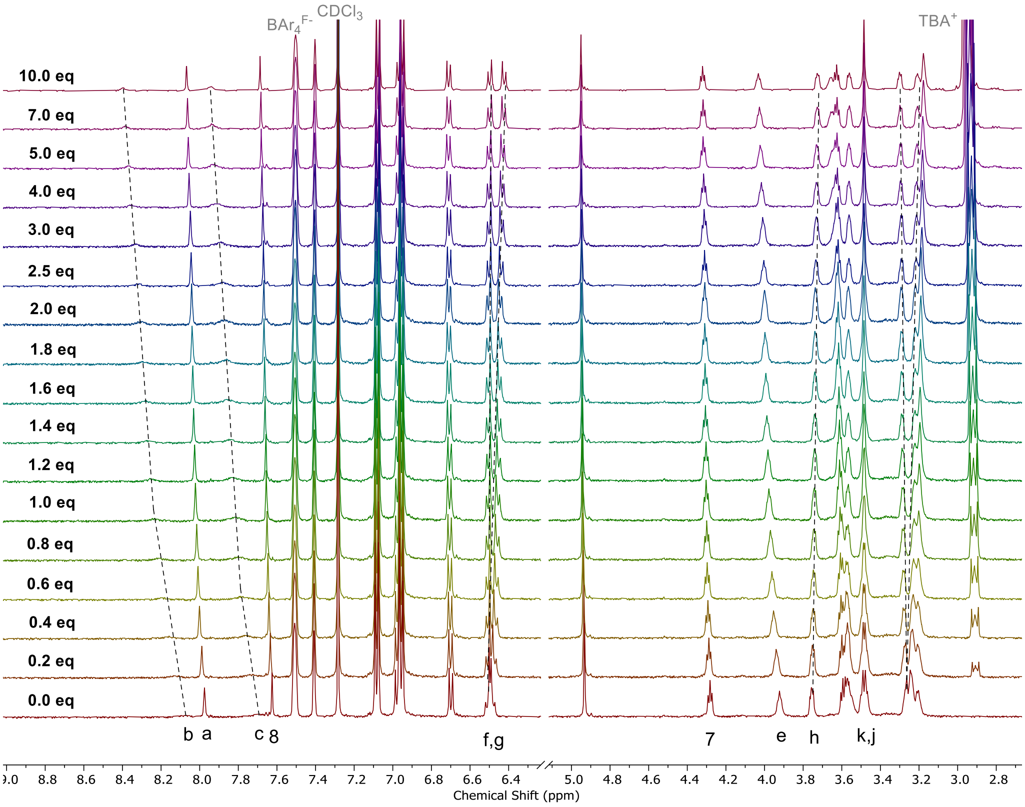

*Figure S46. Truncated ^{1}H NMR titration spectra of [2]rotaxane **7** upon progressive addition of 10 equivalents TBAI in the presence of 1 equivalent $NaBAr^{F}_{4}$ (500 MHz, 298 K, 7:3 $CDCl_3/CD_3CN$, [Receptor] = [$NaBAr_4^F$] = 1.0 mM).*

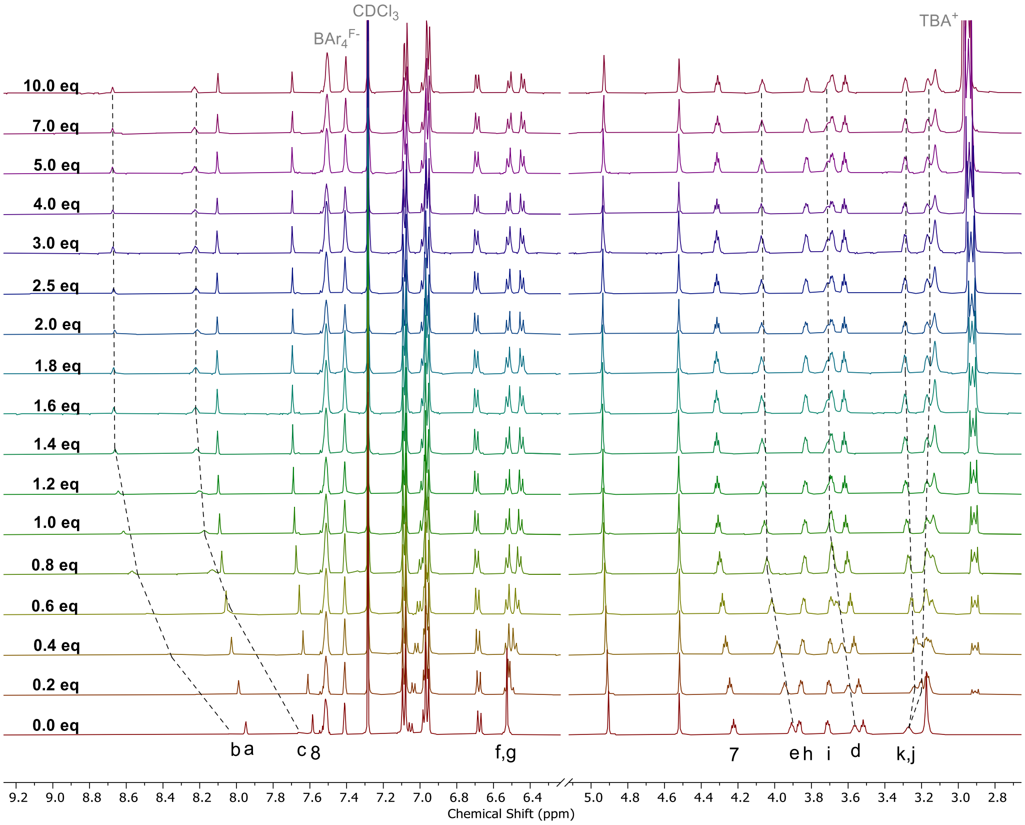

*Figure S47. Truncated ^{1}H NMR titration spectra of [2]rotaxane **8** upon progressive addition of 10 equivalents TBACl in the presence of 1 equivalent $NaBAr^{F}_{4}$ (500 MHz, 298 K, 7:3 $CDCl_3/CD_3CN$, [Receptor] = [$NaBAr_4^F$] = 1.0 mM).*

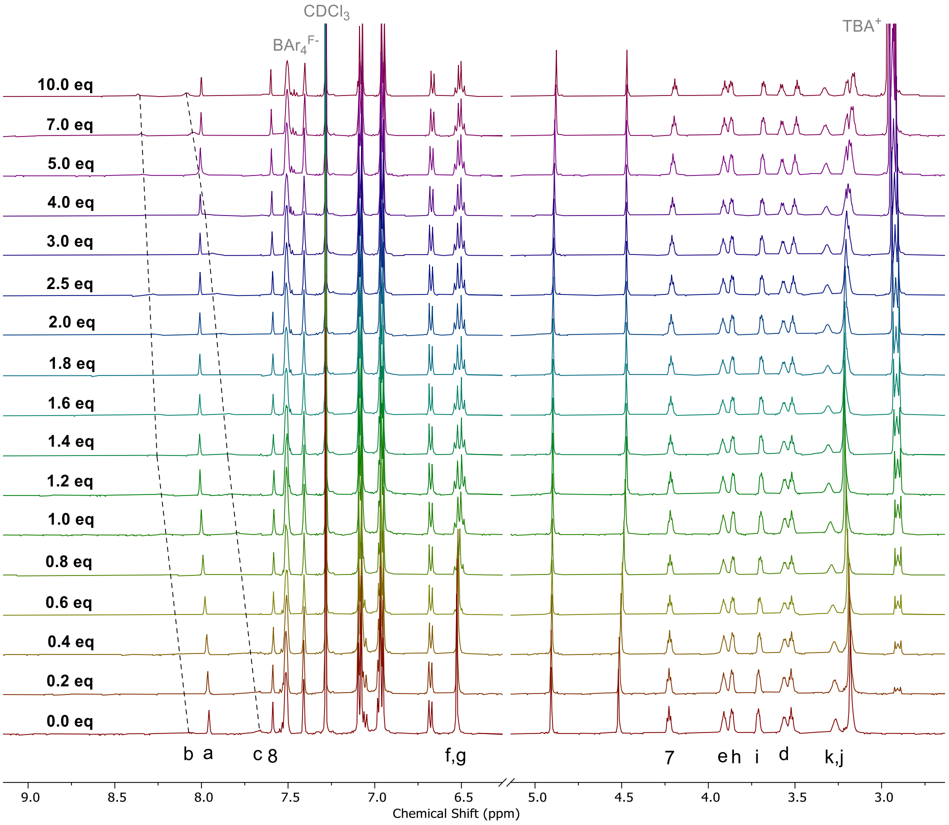

*Figure S48. Truncated ^{1}H NMR titration spectra of [2]rotaxane **8** upon progressive addition of 10 equivalents TBABr in the presence of 1 equivalent $NaBAr^{F}_{4}$ (500 MHz, 298 K, 7:3 $CDCl_3/CD_3CN$, [Receptor] = [$NaBAr_4^F$] = 1.0 mM).*

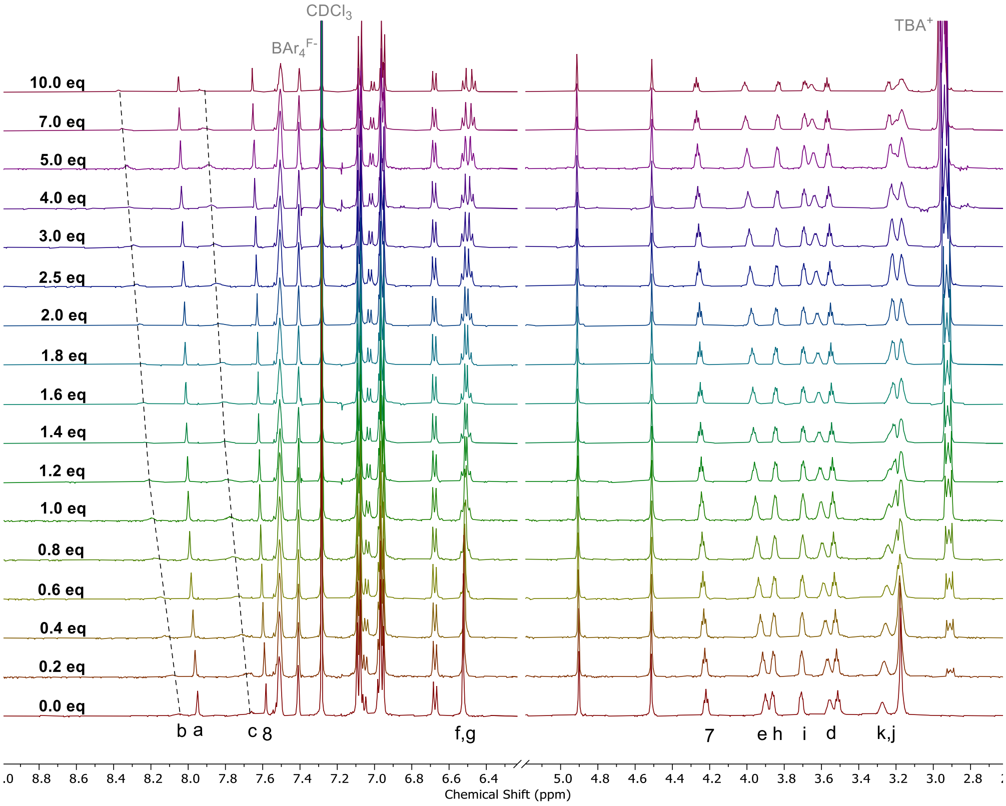

*Figure S49. Truncated ^{1}H NMR titration spectra of [2]rotaxane **8** upon progressive addition of 10 equivalents TBAI in the presence of 1 equivalent $NaBAr^{F}_{4}$ (500 MHz, 298 K, 7:3 $CDCl_3/CD_3CN$, [Receptor] = [$NaBAr_4^F$] = 1.0 mM).*

Binding isotherms

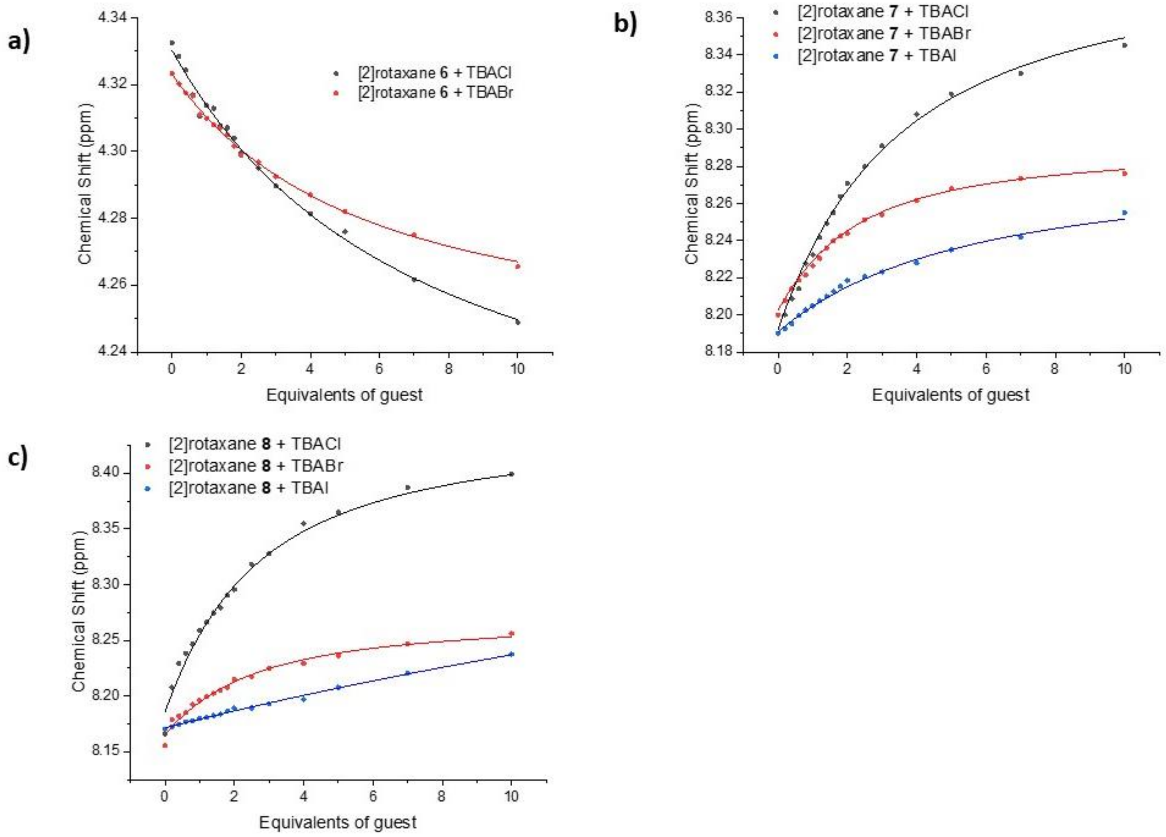

Figure S50. Binding isotherms of [2]rotaxanes (a) ***6*** *showing changes in chemical shift of protons H_7 and (b)* ***7*** *and (c)* ***8*** *showing changes in chemical shift of internal benzene proton H_b with increasing equivalents of TBAX salts (X = Cl^-, Br^-, I^-). ([Receptor] = 1.0 mM, 500 MHz, 298 K, 7:3 $CDCl_3$:CD_3CN)*

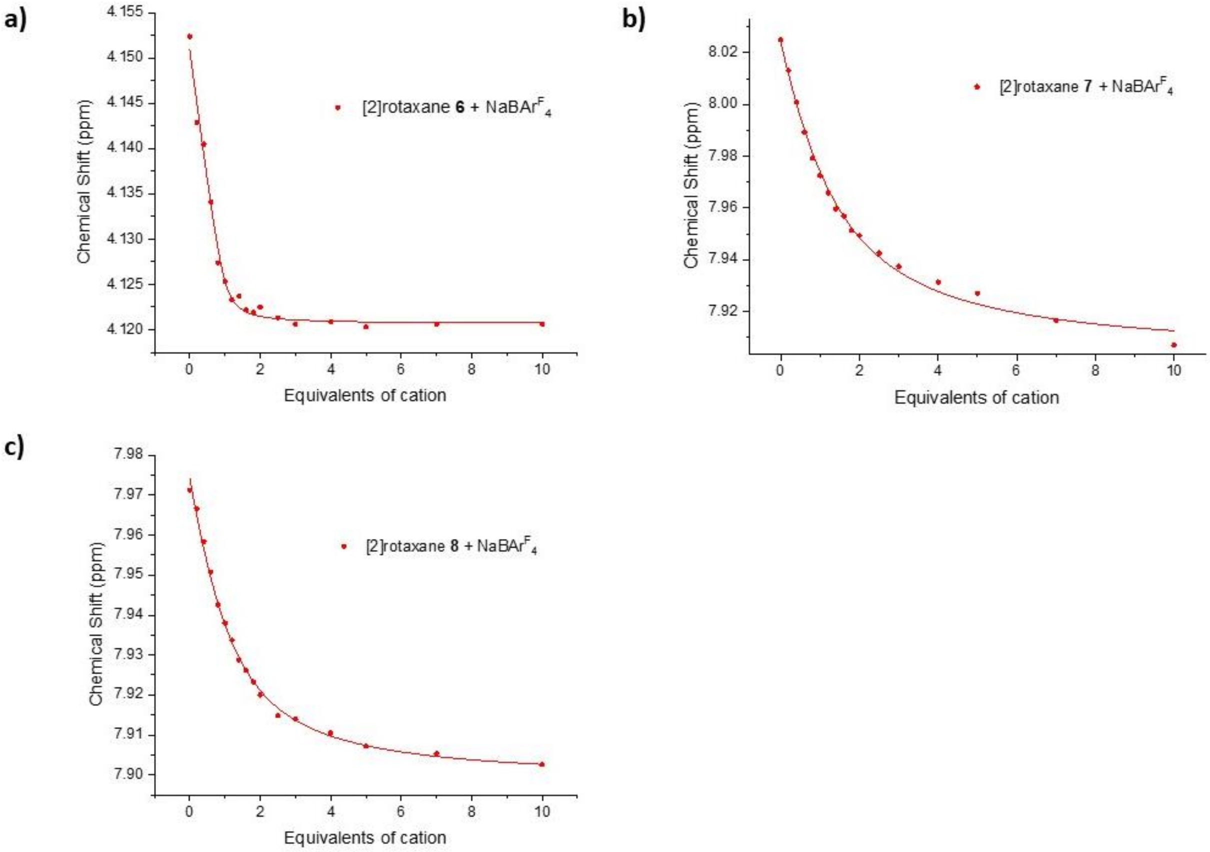

Figure S51. Binding isotherms of [2]rotaxanes (a) ***6*** *(b)* ***7*** *and (c)* ***8****, showing changes in chemical shift of H_b with increasing equivalents of NaBAr$^F{}_4$ salt. ([Receptor] = 1.0 mM, 500 MHz, 298 K, 7:3 $CDCl_3$:CD_3CN)*

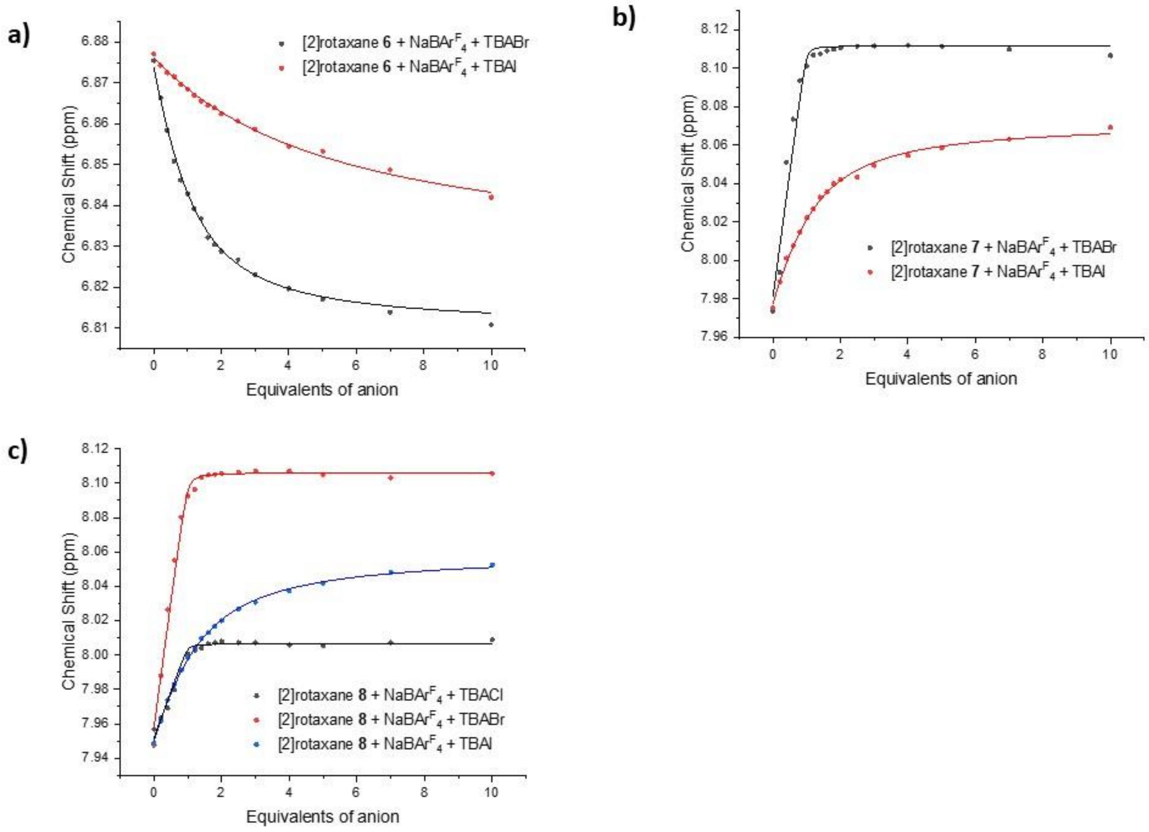

Figure S52. Binding isotherms of [2]rotaxanes (a) ***6*** *showing changes in chemical shift of xylene protons H_a and (b)* ***7*** *and (c)* ***8*** *showing changes in chemical shift of internal benzene proton H_b with increasing equivalents of TBAX salts (X = Br^-, I^-) in the presence of 1 eq. M^IBAr^F (M^I = Na^+, K^+). ([Receptor] = 1.0 mM, 500 MHz, 298 K, 7:3 $CDCl_3$:CD_3CN)*

Solid-liquid extraction experiments

The capability of the receptors to extract solid alkali metal salts into organic solvent was investigated through a series of solid-liquid extraction (SLE) experiments. In a typical experiment, an excess of a solid sodium salt (NaCl, NaBr, NaI) was added to 1.0 mM a solution of the receptor in $CDCl_3$ (700 µL) and the mixture was vigorously sonicated for 20 min. The excess salt was subsequently removed by filtration through a syringe filter. A ^1H NMR spectrum of the filtrate was collected using a Bruker AVIII 500 MHz spectrometer at 298 K.

^1H NMR spectra of the receptors before and after treatment with alkali halide salts

Treatment of [2]rotaxane **6** in the presence of excess NaX (X = Cl⁻, Br⁻, I⁻) led to no extraction of NaCl as evidenced from the imperceptible shifts in the peaks. A high degree of extraction was observed for NaBr whereas NaI, however, expectedly the perturbations were not as significant as the other two rotaxanes.

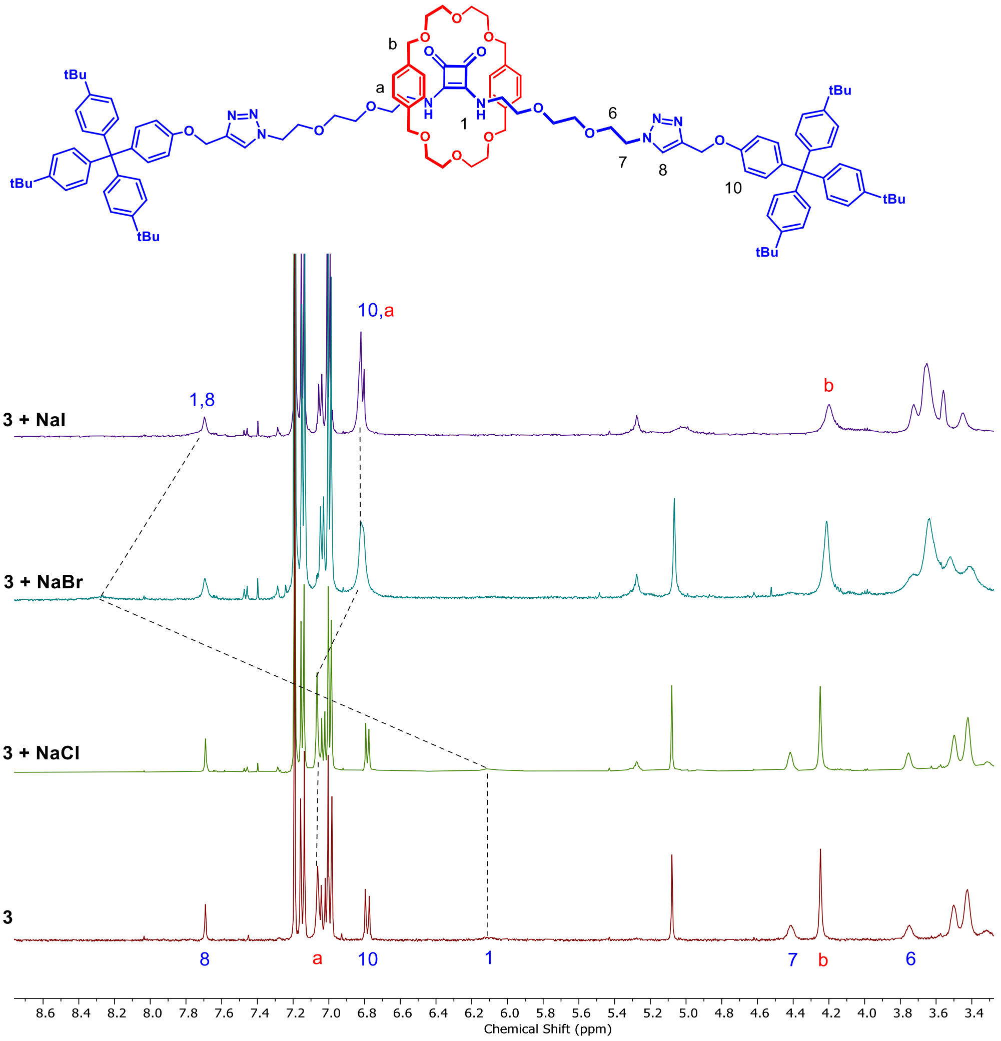

*Figure S53. Pre- and post-extraction ^1H NMR spectra of [2]rotaxane **6** with excess solid NaCl, NaBr and NaI (500 MHz, 298 K, $CDCl_3$).*

When [2]rotaxane **7** was treated with NaX (X = Cl^-, Br^-, I^-), no extraction of NaCl was observed; NaBr and NaI were both sufficiently extracted as evidenced by the significant splitting and shifting of the anion and cation cavity protons. As expected, the shifts in the peaks was greater in the case of NaBr owing to the [2]rotaxane being a better receptor of bromide in the presence of sodium.

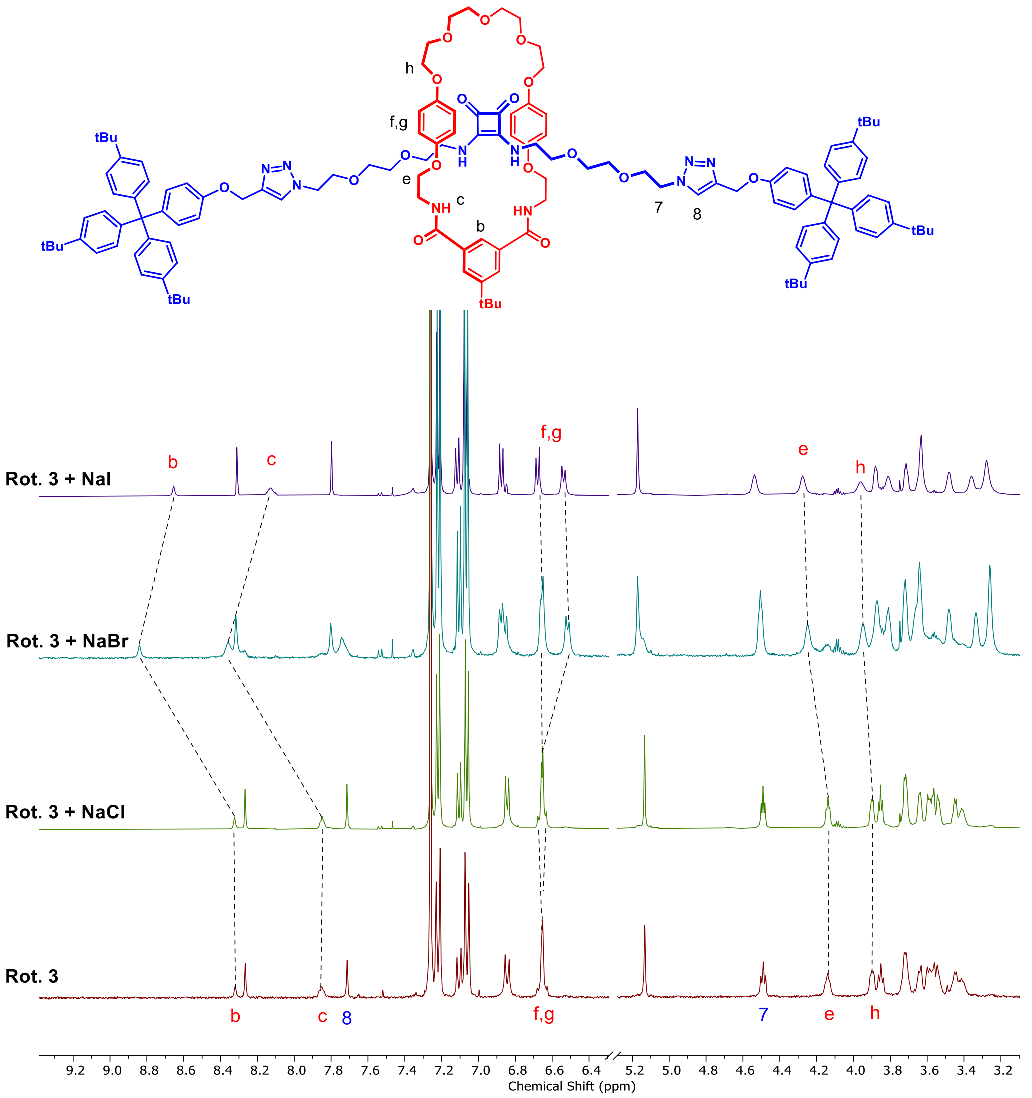

*Figure S54. Pre- and post-extraction ^{1}H NMR spectra of [2]rotaxane **7** with excess solid NaCl, NaBr and NaI (500 MHz, 298 K, $CDCl_3$).*

Analogous SLE experiments with [2]rotaxane **8** in the presence of excess NaX (X = Cl^-, Br^-, I^-) resulted in no extraction of NaCl. A high degree of extraction was observed for NaBr and NaI. As observed in the case of [2]rotaxane **7**, the shifts in the peaks was greater in the case of NaBr owing to the [2]rotaxane demonstrating a preference for bromide in the presence of sodium.

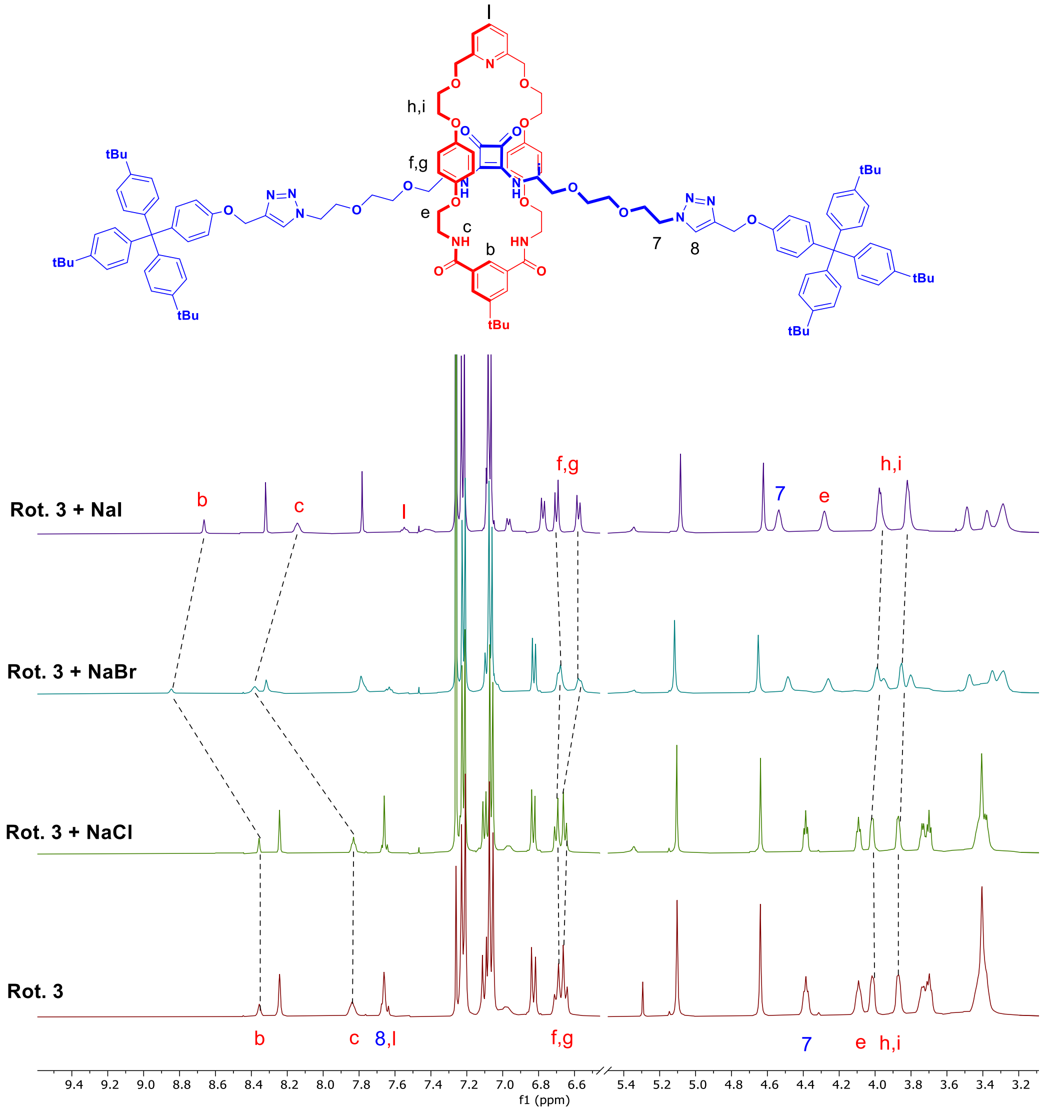

*Figure S55. Pre- and post-extraction ^{1}H NMR spectra of [2]rotaxane **8** with excess solid NaCl, NaBr and NaI (500 MHz, 298 K, $CDCl_3$).*

References

55 V. Aucagne, K. D. Hänni, D. A. Leigh, P. J. Lusby, D. B. Walker, *J. Am. Chem. Soc.* **2006**, *128*, 2186–2187.
56 T. R. Chan, R. Hilgraf, K. B. Sharpless, V. V. Fokin, *Org. Lett.* **2004**, *6*, 2853-2855.
57 R. Samudrala, X. Zhang, R. M. Wadkins and D. L. Mattern, *Bioorg. Med. Chem.* **2007**, *15*, 186-193.
58 P. N. Cheng, C. F. Lin, Y. H. Liu, C. C. Lai, S. M. Pen, S. H. Chiu, *Org. Lett.* **2006**, *8*, 435-438.
59 A. Brown, T. Lang, K. M. Mullen, P. D. Beer, *Org. Biomol. Chem.* **2017**, *15*, 4587-4594.
60 M. R. Sambrook, P. D. Beer, J. A. Wisner, R. L. Paul, A. R. Cowley, F. Szemes, M. G. Drew, *J. Am. Chem. Soc.* **2005**, *127*, 2292-2302.
